# Genomic landscape of liposarcoma

**DOI:** 10.18632/oncotarget.6464

**Published:** 2015-12-04

**Authors:** Deepika Kanojia, Yasunobu Nagata, Manoj Garg, Dhong Hyun Lee, Aiko Sato, Kenichi Yoshida, Yusuke Sato, Masashi Sanada, Anand Mayakonda, Christoph Bartenhagen, Hans-Ulrich Klein, Ngan B. Doan, Jonathan W. Said, S. Mohith, Swetha Gunasekar, Yuichi Shiraishi, Kenichi Chiba, Hiroko Tanaka, Satoru Miyano, Ola Myklebost, Henry Yang, Martin Dugas, Leonardo A. Meza-Zepeda, Allan W. Silberman, Charles Forscher, Jeffrey W. Tyner, Seishi Ogawa, H. Phillip Koeffler

**Affiliations:** ^1^ Cancer Science Institute of Singapore, National University of Singapore, Singapore; ^2^ Department of Pathology and Tumor Biology, Graduate School of Medicine, Kyoto University, Kyoto, Japan; ^3^ Division of Hematology/Oncology, Cedars-Sinai Medical Center, University of California, School of Medicine, Los Angeles, California, USA; ^4^ Department of Advanced Diagnosis, Clinical Research Center, Nagoya Medical Center, Nagoya, Japan; ^5^ Institute of Medical Informatics, University of Münster, Münster, Germany; ^6^ Department of Pathology and Laboratory Medicine, Santa Monica-University of California-Los Angeles Medical Center, Los Angeles, California, USA; ^7^ Laboratory of DNA Information Analysis, Human Genome Center, Institute of Medical Science, The University of Tokyo, Tokyo, Japan; ^8^ Laboratory of Sequence Analysis, Human Genome Center, Institute of Medical Science, The University of Tokyo, Tokyo, Japan; ^9^ Norwegian Cancer Genomics Consortium and Department of Tumor Biology, Institute of Cancer Research, Oslo University Hospital, The Norwegian Radium Hospital, Oslo, Norway; ^10^ Department of Molecular Bioscience, University of Oslo, Oslo, Norway; ^11^ Department of Surgery, Cedars Sinai Medical Center, Division of Surgical Oncology, Los Angeles, California, USA; ^12^ Knight Cancer Institute, Cell and Developmental Biology, Oregon Health and Science University, Portland, Oregon, USA; ^13^ National University Cancer Institute, National University Hospital, Singapore

**Keywords:** liposarcoma, exome sequencing, SNP array, intra-tumor heterogeneity, therapeutics

## Abstract

Liposarcoma (LPS) is the most common type of soft tissue sarcoma accounting for 20% of all adult sarcomas. Due to absence of clinically effective treatment options in inoperable situations and resistance to chemotherapeutics, a critical need exists to identify novel therapeutic targets. We analyzed LPS genomic landscape using SNP arrays, whole exome sequencing and targeted exome sequencing to uncover the genomic information for development of specific anti-cancer targets. SNP array analysis indicated known amplified genes (MDM2, CDK4, HMGA2) and important novel genes (UAP1, MIR557, LAMA4, CPM, IGF2, ERBB3, IGF1R). Carboxypeptidase M (CPM), recurrently amplified gene in well-differentiated/de-differentiated LPS was noted as a putative oncogene involved in the EGFR pathway. Notable deletions were found at chromosome 1p (RUNX3, ARID1A), chromosome 11q (ATM, CHEK1) and chromosome 13q14.2 (MIR15A, MIR16-1). Significantly and recurrently mutated genes (false discovery rate < 0.05) included PLEC (27%), MXRA5 (21%), FAT3 (24%), NF1 (20%), MDC1 (10%), TP53 (7%) and CHEK2 (6%). Further, *in vitro* and *in vivo* functional studies provided evidence for the tumor suppressor role for Neurofibromin 1 (NF1) gene in different subtypes of LPS. Pathway analysis of recurrent mutations demonstrated signaling through MAPK, JAK-STAT, Wnt, ErbB, axon guidance, apoptosis, DNA damage repair and cell cycle pathways were involved in liposarcomagenesis. Interestingly, we also found mutational and copy number heterogeneity within a primary LPS tumor signifying the importance of multi-region sequencing for cancer-genome guided therapy. In summary, these findings provide insight into the genomic complexity of LPS and highlight potential druggable pathways for targeted therapeutic approach.

## INTRODUCTION

Liposarcoma (LPS) is a rare cancer with a high recurrence rate and low response to existing therapies [1]. To date, surgery is the only therapeutic strategy for treating aggressive LPS, but most of these tumors recur and metastasize associated with a high mortality [2]. LPSs are classified according to World Health Organization into five types [3], 1) Atypical lipomatous tumors (ALT)/Well-differentiated LPS (WDLPS); 2) De-differentiated LPS (DDLPS); 3) Myxoid LPS (MLPS); 4) Pleomorphic LPS (PLPS) and 5) Mixed-type LPS. Among these, WDLPS and DDLPS are the most frequent types occurring in 40-50% of all LPS cases and are characterized by the presence of supernumerary ring and/or giant marker chromosomes [4]. The other common type, MLPS is characterized by the recurrent translocation t(12;16)(q13;p11) that results in the FUS-CHOP gene fusion present in over 95% of MLPS cases [5]. PLPS is a rare variety with limited molecular studies.

Studies characterizing the genetic alterations in LPS have focused on analysis of copy number, mRNA expression and DNA sequences of limited number of candidate genes. Several groups have discovered amplification on chromosome 12q in the majority of WDLPS and DDLPS patients including *HMGA2*, *CDK4* and *MDM2* oncogenes [6, 7]. A DNA sequencing study identified frequent mutations of *TP53* and *RB1* in PLPS, *NF1* in PLPS and *PIK3CA* and *KIT* in MLPS patients [7]. In addition, next generation sequencing approach revealed structural complexities in primary and locally recurrent DDLPS samples and discovered recurrent mutations of *HDAC1*, *MAPKAP1*, *PTPN9* and *DAZAP2* [8]. Despite these previous reported genetic studies in LPS, no single drug against any genomic target in this disease is yet approved; necessitating the drive to find and validate clinically relevant therapeutic targets in this disease. Since LPS remains relatively underserved by large sequencing groups, we pursued to define the genomic landscape using SNP Chip and next generation sequencing to identify the full spectrum of driver mutant genes and altered pathways in different types of LPS.

Here, we report a variety of genomic aberrations including mutations, and copy number changes in different types of LPS using SNP-CHIP array, whole exome sequencing (WES), and targeted exome sequencing (TES). The present study defines the genetic landscape of LPS highlighting a potentially druggable alteration of *Carboxypeptidase M* (*CPM*) gene. Integrative exome sequencing, SNP analysis and biological studies revealed tumor suppressor role of *Neurofibromin 1* (*NF1*) in LPS. Pathway analysis of frequently altered genes indicated involvement of various important pathways such as signalling through MAPK and Erb in LPS leading to identification of genes that can be potentially targeted using currently available drugs. Comprehensive genomic characterization efforts to catalog altered genes and pathways will improve our understanding of the molecular genetics of LPS for better management of the disease.

## RESULTS

We analyzed LPS samples of different types through a combination of next generation sequencing approach including SNP arrays of 86 patients and 13 cell lines, WES of 12 patients, and TES of 86 patients and 13 cell lines (Supplementary Table S1).

### Copy number analysis of LPS samples

**Figure 1:**
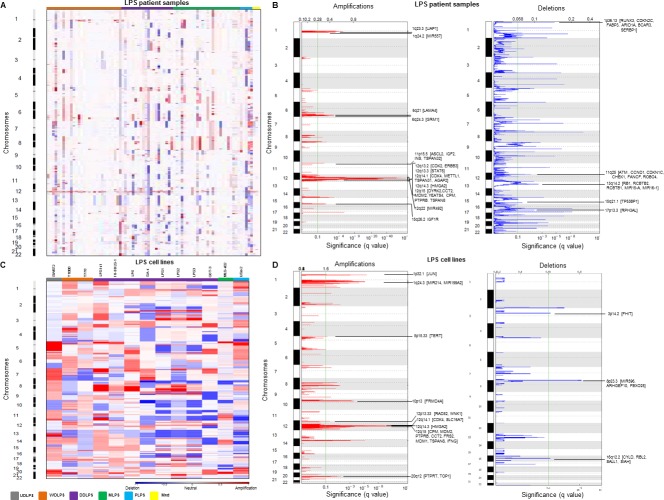
Copy number alterations (CNAs) in LPS samples and cell lines Heat map of the CNAs of **A.** 86 LPS patient samples and **C.** 13 LPS cell lines grouped by various histotypes. Red and blue represents copy gain and loss, respectively in units of log_2_(cancer/normal). GISTIC analysis demonstrating significant genomic amplifications (red) and deletions (blue) in the **B.** LPS patient samples and **D.** LPS cell lines genome with chromosome number along the y axis and statistical significance of the aberrations displayed as FDR q values along the x axis. Chromosomal peaks and the cancer-related genes within those peaks are shown.

SNP array analysis of 86 LPS patient samples revealed chromosomal regions most frequently aberrant as shown in the heat map (Figure 1A) grouped according to subtypes. Among the recurrent copy number amplifications, we found previously reported classical amplicon at chromosome 12q13-15 involving important well-known oncogenes such as [*HMGA2* [9], *CDK4* [7], *MDM2* [7] and miR-26-a2 [10] in WDLPS and DDLPS patient samples. In addition, GISTIC analysis indicated statistically significant, frequently observed broad and focal amplifications at chromosomes 1q, 6q, 8q, 11p, 12q, 14q and 15q (Figure 1B). Interestingly, we observed important but previously not reported genes: *UAP1*, *MIR557*, *LAMA4*, *GRM1*, *IGF2*, *CPM*, *IGF1R*, *ERBB3*, *STAT6*, and *MIR492* in the amplified regions. Significant deletions were observed at chromosomes 1p, 3p, 6p, 11q, 13q, 15q and 17p (Figure 1B) indicating important novel aberrated genes: *RUNX3*, *CDKN2C*, *ARIDIA*, *BCAR3*, *CDKN1C*, *CCND1*, *MIR15-A*, *MIR16-1*, *TP53BP1*. All the significantly altered regions and genes within the regions identified in LPS samples using GISTIC analysis are listed in Supplementary Table S2.

Of note, SNP array of 13 LPS cell lines (included all the histological types of LPS) displayed comparatively more copy number aberrations than the patient samples as seen in the heat map (Figure 1C). Heat map showed that unlike the other cell lines, SW872 did not have the common copy number alterations (CNAs) (gain at chromosomes 1, 2, 12 and loss at chromosomes 11, and 13). MLS402 cells had fewer CNAs compared to other LPS cell lines, thus more resembling the profiling pattern of the LPS patient samples. GISTIC analysis indicated important and significantly recurrent broad amplifications and deletions in LPS cell lines including amplification at chromosomes 1p (*JUN*, *MIR214*, *MIR1992A*), 5p15.33 (*TERT*), 10p13, 12q13-15, and 20q12 (Figure 1D). Important and novel deletions were identified at chromosomes 3p14.2 (*FHIT*), 8p23.3 (*MIR596, ARHGEF10, FBXO25*), and 16q12.2 (*CYLD, RBL2, SALL1*) (Figure 1D). List of regions and genes observed in GISTIC analysis is provided in Supplementary Table S3. The focal amplifications and deletions noted in the cell lines provide a suitable platform for biological studies (Table 1).

**Table 1:** Important and selected copy number alterations in LPS cell lines relevant to transformation

Cell Line(s)	Alteration	Chromosome	Selected Gene(s)
SW872	HD	10q23.31	*PTEN*
T1000, T778	AMP	4p15.2	*PPARGC1A*
LPS141	HD	9p23	*PTPRD*
MLS402	HD	3p14.2	*FHIT*
MLS402	UPD	13q14.2-14.3	*RB1, SETDB2, RCBTB1, DLEU2, MIR15-A, MIR16-1,*
T778, FU-DDLS-1, LP6, MLS402	AMP	5p15.33	*TERT*
FU-DDLS-1, MLS402, LPS141,	AMP	5p15.2	*CTNND2*
MLS402	AMP	11q22	*YAP1, BIRC2, BIRC3*
MLS402	AMP	14q22.2	*CDKN3*
LiSa-2	AMP	19q13	*PAK4, AKT2*
LiSa-2	HD	13q	*RB1, SETDB2, RCBTB1, DLEU2, MIR15-A, MIR16-1,*
LiSa-2	HD	3p24	*RBMS3*
GOT-3	HD	9q33.1	*DEC1*
LPS141, GOT-3	HD	16q23	*WWOX*
GOT-3	AMP	Xp22.31	*PNPLA4*
LPS1, LPS2, LPS3, SA4,	HD	9p21.3	*CDKN2A, CDKN2B*
SA4	HD	10p14	*GATA3*
SA4	HD	10p11.21	*PARD3*
SA4	HD	15q15.3	*TP53BP1*
LiSa-2	HD	16q12.2	*RBL2, CYLD, SALL1, SIAH*
FU-DDLS-1, LPS1, LPS2, LPS3, LP6	AMP	1p32.1	*JUN*

HD: homozygous deletion; AMP: amplification; UPD: uniparental disomy

### Frequent amplification of *CPM* gene in WDLPS/DDLPS

**Figure 2:**
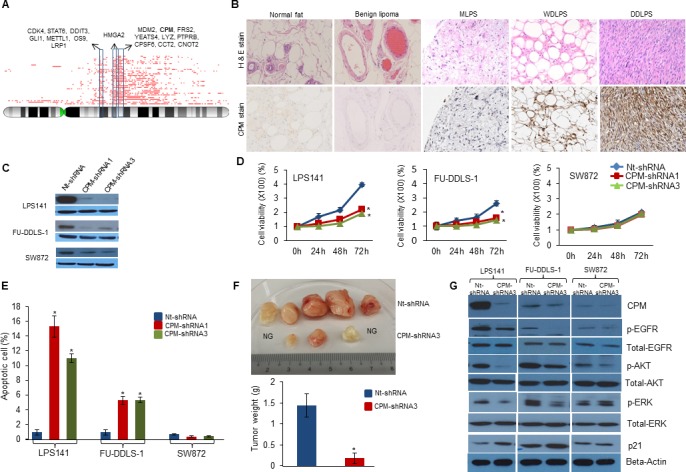
Role of *CPM* in liposarcomagenesis **A.** Recurrent genomic copy number gains (red lines) at chromosome 12q13-15 demonstrated as integral chromosome view (top) using CNAG SNP analysis of WDLPS and DDLPS patient samples. Red lines on top shows the recurrent amplified regions along with the important genes. **B.** Immunohistochemical staining (IHS) of CPM protein in LPS tissue microarray. H&E staining (top row) shows the morphology and CPM IHS (bottom row) of tissue of normal fat, benign lipoma, MLPS, WDLPS and DDLPS (x100). **C.** Stable silencing of *CPM* gene in LPS cell lines using shRNA confirmed by Western blot (GAPDH, internal control). **D.** Effect of *CPM* knockdown on cellular proliferation using MTT assay in LPS141, FU-DDLS-1 and SW872 cells compared to Non-target (Nt)-shRNA. **E.** Effect of *CPM* shRNA on apoptosis of LPS cells using Annexin-V-FITC and Propidium iodide staining followed by flow cytometry. **F.** Effect of *CPM* shRNA3 on LPS141 xenograft growth i*n vivo* in NSG mice. Top panel shows dissected tumors from the control (Nt-shRNA) and *CPM*-shRNA3 tumor containing mice. No growth (NG). Bottom panel: Tumor weight was significantly lower in *CPM* knockdown tumors compared to Nt-shRNA tumors. **G.** *CPM*-shRNA3 and Nt-shRNA expressing LPS141, FU-DDLS-1, and SW872 cells were analyzed using a variety of antibodies to detect changes in EGFR signaling. (Actin, internal loading control). Values of Panels **D.**-**F.** are presented as the mean ± SE (*n* = 3). * *P* < 0.05 compared with the control group. [Note: Value of S.E. are too small to be visible in Panel D]

In addition to the above mentioned recurrent copy number aberrations, we detected gain/amplification of the *CPM* gene at chromosome 12q15 in 78% (39/50) of WDLPS/DDLPS patient samples (Figure 2A) which was validated using genomic quantitative PCR (Supplementary Fig. S1A). CPM protein levels were significantly higher in WDLPS/DDLPS samples as shown by positive immunohistochemical staining, whereas the protein was not detectable in benign lipoma and normal fat tissue (Figure 2B and Supplementary Fig. S1B). In addition, *CPM* was amplified in T1000, T778, LPS141, FU-DDLS-1, SA-4, LPS1, LPS2 and LPS3 cells. Western blotting revealed high CPM expression in all CPM amplified cell lines compared with the non-amplified SW872 cells (Supplementary Fig. S1C). Flow cytometry showed CPM surface expression on these CPM amplified cell lines suggesting this enzyme may be an attractive therapeutic target (Supplementary Fig. S1D).

Functional role of *CPM* was characterized in LPS141 and FU-DDLS-1 cells (*CPM* amplification) compared to SW872 cells (without *CPM* amplification). *CPM* knockdown using siRNA1 and siRNA2 *versus* scramble siRNA inhibited cell proliferation of LPS141 and FU-DDLS-1, but not in SW872 (Supplementary Figs. S2A and S2B). To analyze long-term effects of *CPM* knockdown, lentivirus containing *CPM* shRNA was stably infected into these cells (Figure 2C) resulting in significant reduction in cell growth (Figure 2D), colony formation, migration and invasion (Supplementary Figs. S2C-E) in LPS141 and FU-DDLS-1 (not in SW872). Also LPS141 and FU-DDLS-1 CPM shRNA expressing cells had significantly increased apoptosis compared to SW872 cells (Figure 2E and Supplementary Fig. S2F). In addition, a significant decreased *in vivo* tumor growth of *CPM* knockdown LPS141 cells was observed compared to wild type LPS141 cells in NSG mice (Figure 2F). One important function of *CPM* is enzymatic cleavage of the C-terminal arginine of epidermal growth factor (EGF) in tissues suggesting *CPM* may be involved in activation of *EGF*/*EGFR* signalling [11]. We found that *CPM* knockdown decreased expression levels of phosphorylated EGFR, Akt, and ERK in LPS141 and FU-DDLS-1 cells but not in SW872 cells (Figure 2G). Levels of p21 protein increased upon *CPM* knockdown in LPS141 and FU-DDLS-1 cells compared to non-target shRNA (Nt-shRNA) control cells. Taken together, high levels of CPM in LPS cells stimulate the transformed features of LPS.

### Discovery of somatic mutations through WES

**Figure 3:**
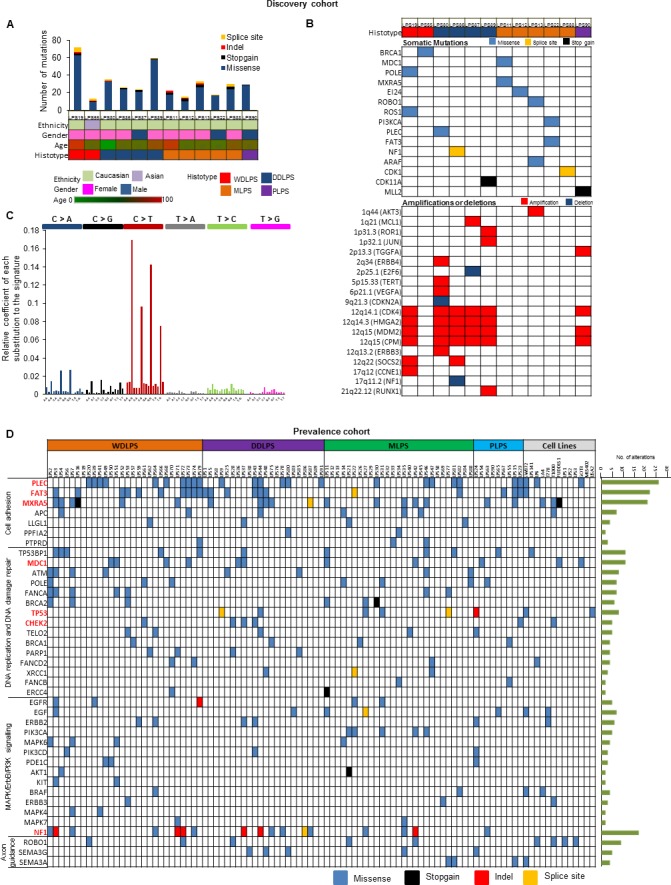
Genetic heterogeneity of LPS using WES and TES **A.** Total number of alterations observed by WES in patient samples in Discovery cohort (Top Panel). Patient characteristics are shown in second panel. **B.** Mutational profiling using WES and copy number profiling (SNP array) in Discovery cohort. **C.** Mutational signature found in LPS Discovery Cohort. X axis demonstrate mutational type; Y axis show relative coefficient of each substitution to the signature. Six types of substitutions are displayed in different color probability bars. **D.** Topography of validated genomic alterations in LPS Prevalence cohort and 13 LPS cell lines. Each column represents an individual patient grouped according to histotype and cell lines; and each row indicates a gene. Number of mutations detected for each gene across the cohort represented to the right of the heat map. Significantly altered genes are highlighted in bold and red color.

WES was performed on 12 LPS human samples of different types and their matched normal tissues as a Discovery Set. Average coverage was 185-fold and 80% of bases were covered efficiently for variant calls (≥20X coverage) (Supplementary Table S4, Supplementary Fig. S3). A total 377 potential somatic changes were identified and Sanger sequencing validated 91% of events (Figure 3A, Supplementary Table S5). Clinical characteristics of the patients along with some of the important mutated genes (*BRCA1*, *MXRA5*, *NF1*, *CDK11A* and *CDK1*) and copy number changes (*CPM*, *JUN*, *PIK3CA*, *CDKN2A*, *CDK4*, *HMGA2*, *MDM2*, *NF1*) are shown in Figure 3B. Cancers often have variable mutational spectra pointing to particular mutagenic stimuli [12]. Only mutational signature 1 (Figure 3C) was observed in the Discovery cohort in which majority of changes were C > T transitions and C > A transversions representing involvement of an endogenous mutational process due to deamination of 5-methyl-cytosine [12].

### Landscape of LPS mutations

Prevalence Set of an additional 86 LPS patient samples (Supplementary Table S1) and 13 cell lines were examined by TES. 248 genes (somatically mutated in the Discovery Set and well-known cancer-related genes) were evaluated (Supplementary Table S5). Average coverage was 121-fold, and 79% of bases were covered efficiently for variant calls (≥ 20X coverage) (Supplementary Table S6). After removing mapping errors and known SNPs, a total of 995 non-synonymous mutations including insertions/deletions were identified (Supplementary Fig. S4A, Supplementary Table S7) in patient samples. Mutational Significance in Cancer (MuSiC) tool was used to identify significantly altered genes; important mutated genes included *MXRA5*, *PLEC*, *NF1*, *FAT3, TP53*, *MDC1*, *CHEK2* and *MDC1* which were significantly enriched for mutations (q value < 0.05) (Supplementary Table S8). Recurrent mutations in previously unidentified genes were found in LPS samples associated with cell adhesion and cytoskeleton organization [51 of 86 (60%) patients] including mutant *PLEC*, *FAT3*, *MXRA5*, *APC*, *LLGL1*, and *PTPRD* (Figure 3D, Supplementary Fig. S4B). Altered genes in DNA replication, DNA damage checkpoint and repair pathways were detected in 65% of cases. Genetic alterations in the cell cycle pathway were found in G1/S transition control due to amplifications of *CDK4* and *MDM2* in 70% of samples, including those important mutations or deletions of *RB1*, *CDKN2A*, *CHEK2*, *TP53* and *ATM* genes. Mutations of genes critical for various tyrosine kinase pathways were also identified in 36% of samples (Figure 3D).

**Figure 4:**
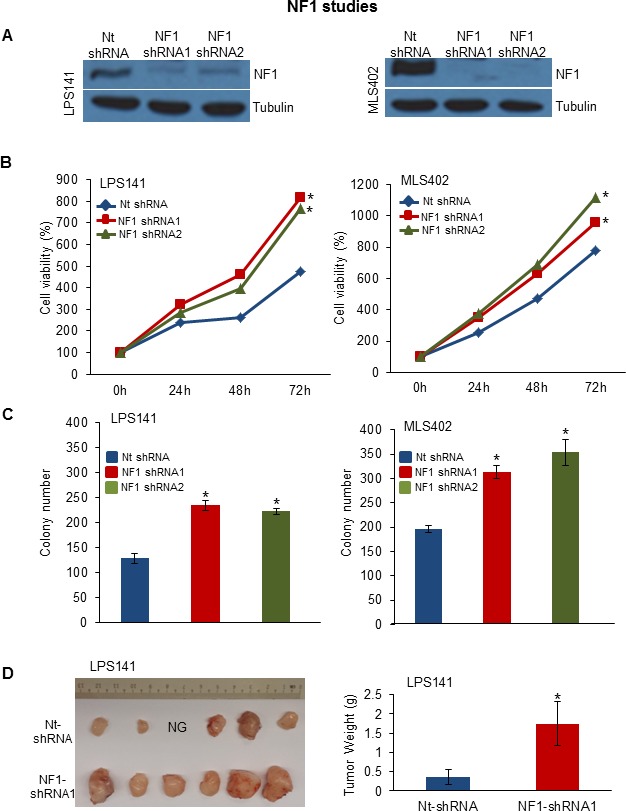
Functional analysis of altered *NF1* gene **A.** Western blotting analysis of LPS141 and MLS402 cells stably infected with *NF1* shRNA1, *NF1* shRNA2 and Nt-shRNA (control) after 72 h of transduction. **B.** Proliferation assay at 24, 48, and 72 hours using MTT. **C.** Soft agar clonogenic growth of NF1 knockdown cells compared to Nt-shRNA. **D.** *In vivo* xenograft assay of NF1-knockdown of LPS141 cells compared to Nt-shRNA infected cells (NG, no growth). Bar graph (right) indicate tumor weight of NF1 knockdown xenografts compared to Nt-shRNA. All assays are representative of three independent experiments (mean ± S.E.; *P < 0.05). Unpaired 2 tailed Student's *t* test was used to calculate all *P* values. [Note: Value of S.E. are too small to be visible in the panel B]

Interestingly, a class of genes involved in axon guidance pathway (*ROBO1*, *SEMA3G* and *SEMA3A*) were mutated in patient samples (Figure 3D). *ROBO1* was also mutated in 4 LPS cell lines [LPS1, LPS3, LP6 and T1000] (Supplementary Table S7). Genomic aberrations in these genes in LPS have not been reported. Using TES and copy number analysis, we identified *NF1* mutations (including 5 frameshift insertions) in 17 patient samples and loss of heterozygosity in 5 LPS patient samples (Supplementary Figs. S5A-B). *NF1* silencing using shRNA (Figure 4A) significantly increased cell proliferation (Figure 4B) and colony formation (Figure 4C) in LPS141 and MLS402 cells. *In vivo* stable *NF1* knockdown resulted in increased human LPS growth in a NSG xenograft model (Figure 4D) suggesting a tumor suppressor role of this gene that is frequently aberrant in different types of LPS.

**Figure 5:**
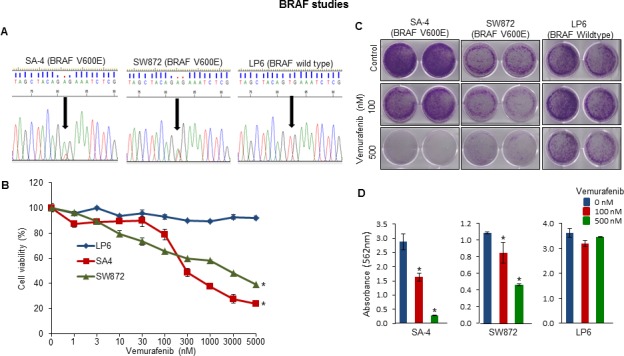
Functional analysis of altered *BRAF* gene in LPS cell lines **A.** Sanger sequencing analysis of oncogenic BRAF (V600E) mutation in the LPS cell lines. Arrow indicates the position of mutation which was only present in SA4 and SW872 cells. **B.** Cell viability by MTT assay; **C.** anchorage-dependent colony assay of SW872, SA4 and LP6 cells treated with either BRAF mutant inhibitor vemurafenib or DMSO. **D.** Absorbance readings of stained colonies. All assays are representative of three independent experiments (mean ± S.E.; *P < 0.05). Unpaired 2 tailed Student's *t* test was used to calculate all *P* values.

Screening of LPS cell lines with targeted capture baits revealed various important gene mutations (*TP53*, *ROBO1*, *FAT3*, *CDKN2A*, *MXRA5*, *PLEC* and *BRAF*) (Supplementary Table S7). SA-4 and SW872 had the most common *BRAF* oncogenic mutation (V600E) and also two LPS patient samples had *BRAF* missense mutations (Figure 5A and Supplementary Fig. S5C). In further studies, SA4 (BRAF V600E), SW872 (BRAF V600E) and LP6 (BRAF Wild-type) cells were cultured in the presence of a small molecule BRAF V600 mutant inhibitor vemurafenib. Only the BRAF mutant cells were sensitive to the growth inhibitory effects of the vemurafenib compared to the BRAF wild-type cells as measured by cell growth in liquid culture (Figure 5B) and clonogenic assay (Figures 5C and 5D).

### Pathways in liposarcoma

We performed pathway analyses of recurrently mutated genes of LPS samples using PathScan algorithm for improved and better understanding of potential mechanism of liposarcomagenesis (Supplementary Table S9). Pathways potentially involved in the disease include signaling through MAPK (*P* = 1.78 × 10^−14^), ErbB (*P* = 3.1 × 10^−11^), JAK-STAT (*P* = 2.5 × 10^−11^), and Wnt (*P* = 4.1 × 10^−7^) as well as apoptosis (*P* = 3.6 × 10^−13^), cell cycle (*P* = 0.0004), DNA replication (*P* = 0.009) and repair (*P* = 0.0003), and axon guidance pathway (*P* = 0.0002).

### Somatic intra-tumoral genetic heterogeneity analysis

**Figure 6:**
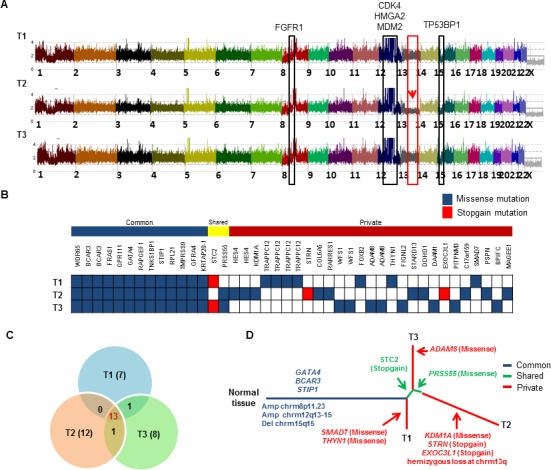
Landscape of intra-tumor heterogeneity **A.** CNAG copy number plot of three different tumor regions (T1, T2 and T3) of the same LPS tumor. Copy number gain and loss of several genes are highlighted. Red arrow indicates the hemizygous loss of chromosome 13q only in region T2. **B.** Distribution of somatic mutations found by WES. Each row indicates the tumor region, and each column represents validated somatic mutations. Mutations are grouped as “common” to all 3 tumors, “shared” are common mutations shared by only 2 of the 3 regions, and “private” found only in single tumor region. **C.** Venn diagram shows distribution of mutations across all three tumor regions. **D.** Phylogenetic relationships of three different tumor regions. Potential driver alterations are indicated in the branch (arrows).

WES and SNP arrays were performed on three different regions (T1, T2, and T3) of the patient's large DDLPS tumor along with germline normal control (N). Average coverage of WES data was 155-fold and 83% of bases were covered for variant calling (> 20X coverage). SNP array identified frequent shared copy number events in all three tumor regions including *FGFR1* amplification at chromosome 8p11.23 and classical *CDK4*, *HMGA2* and *MDM2* amplification at chromosome 12q13-15 region and deletion of TP53BP1 at chromosome 15q15 (Figure 6A). However, region T2 exclusively contained hemizygous loss at chromosome 13q including important genes [*RB1*, *CCNA1*, *CDC16*, *SETDB2*, *SOX1*, *MIR15A* and *MIR16-1*]. We identified 42 somatic mutations in total that were validated by Sanger sequencing and mapped according to their tumor regions (Figure 6B). These mutations were categorized into 13 common mutations, 2 shared mutations (present in 2 of the 3 regions), and 27 private mutations (specifically present in a single region). Ongoing regional clonal evolution was indicated by the presence of unique mutations in each region: T1 (7), T2 (12) and T3 (8) (Figure 6C). A phylogenetic tree of different tumor regions by clonal ordering shows branching tumor evolution (Figure 6D). Only 31% (13 out of 42) of the total mutations were detected in all three regions of this single tumor highlighting the inadequacy of mutational interrogation at only a single tumor site.

## DISCUSSION

LPS, although rare, is one of the most common soft tissue sarcomas with substantial morbidity and mortality. Previous studies have characterized genetic aberrations in LPS tumors identifying recurrent mutations of *TP53*, *PIK3CA*, *RB1*, and *PTEN* [7]. As a compliment to prior studies, we examined all histologic types of LPS by deep nucleotide sequencing and CNA analyses and focused on several aberrant genes.

*CPM* gene is present in one of the multiple-peaks of amplification on chromosome 12q. It belongs to the carboxypeptidases family and is a GPI anchored membrane-bound enzyme [13]. It specifically removes C-terminal basic residues (Arg or Lys) from peptides and proteins, and plays a role in the control of peptide hormone and growth factor activity at the cell surface [14]. Recent reports have suggested that *CPM* can be a potential cancer biomarker to discriminate WDLPS from lipomas [15] when coexpressed with *EGFR* with poor prognosis in adenocarcinoma of lung [16]. Assessment of copy number of both *MDM2* and *CPM* has been proposed as a complementary tool for better classification of LPS tumors [17]. We showed that CPM could modulate EGFR signaling, and silencing of CPM decreased tyrosine kinase activity of EGFR. These observations suggest that lowering the enzymatic activity of CPM may have a therapeutic effect in patients with CPM amplification. We also noted that deletion at chromosome 13q14.2 occurred in approximately 16 out of 86 (19%) of LPS samples and cell lines. This deletion very frequently occurs in chronic lymphoid leukemia (CLL) [18], mantle cell lymphomas [19], multiple myeloma [20] and prostate cancers [21]. This region contains important tumor suppressors *RB1*, *MIR15A* and *MIR16-1*. Studies in CLL have shown BCL2 as the target of miR15/16 [22] and that ABT-199 (Bcl2 inhibitor) is effective against these Bcl-2 elevated lymphoid malignancies [23]. Future studies need to measure levels of the anti-apoptotic proteins (Bcl-2) and determine if ABT-199 or similar drugs are effective in treatment of patients with LPS having a deletion of chromosome 13q14.2.

In the field of cancer genomics, differentiation between somatic drivers of tumorigenesis *versus* passenger mutations is difficult. Mutation significance algorithm MUSIC [24] was used to identify genes (*MXRA5*, *PLEC*, *NF1*, *FAT3* and *MDC1*) showing positive selection for mutation. This algorithm identifies genes that were mutated more often than expected by chance given the background mutation processes [24].

We identified recurrent mutations in previously unidentified genes in LPS, associated with cell adhesion, pathways involved in cytoskeleton organization, base excision repair, homologous recombination repair, nucleotide excision repair, as well as DNA replication process. Alteration of DNA damage repair genes can foster initiation of cancer by preventing senescence and apoptosis, and promoting cellular proliferation even in the presence of accumulation of DNA damage [25]. Another class of mutations includes semaphorins, slits, netrins and ephrins and these genes have important regulatory roles in axon guidance and in cancer cell growth, survival, invasion and angiogenesis (22). This pathway has been recently also found to be aberrated in pancreatic cancer [26]. Mutant genes of this class in LPS include *ROBO1*, *SEMA3G* and *SEMA3*A. Our study strongly suggest that dysregulation of axon guidance pathway may be associated with liposarcomagenesis which requires further investigation.

*NF1* gene encodes Neurofibromin which has significant homology to GTPase-activating proteins (GAPs) [27]. *NF1* negatively regulates the RAS proto-oncogene by catalyzing hydrolysis of RAS-GTP; the latter is important for cell proliferation, differentiation and migration [28]. Mutations of the *NF1* gene in other types of cancer are associated with a highly aggressive often fatal outcome [29]. We found that *NF1* gene was among the most recurrently aberrant gene in our LPS samples (20%); these included missense mutations, frameshift insertions and loss of heterozygosity. An earlier DNA sequencing study reported *NF1* mutations only in PLPS type [7] but in our cohort mutations occurred irrespective of subtype. In further studies, we provided evidence for a potential tumor suppressor role of *NF1 in vitro* and *in vivo*.

Mutational activation of *BRAF* gene is already known to occur in melanoma, glioblastoma, thyroid, lung, colon and hematological malignancies [30]. We identified an oncogenic *BRAF* (V600E) mutation in 2 LPS cell lines [SW872 [30] and SA4] and noted unique *BRAF* mutations in 2 LPS fresh samples. BRAF mutant (V600E) melanoma and thyroid cancers are sensitive, but colon cancers are resistant to therapy with vemurafenib (FDA approved drug) [31]. LPS can now be added to the list of vemurafenib-sensitive tumors. LPS cells carrying *BRAF* V600E mutations were sensitive to inhibition by this tyrosine kinase inhibitor while cells without the mutation were resistant to the drug.

This study for the first time reported multi-region WES and SNP Chip analysis of a LPS patient's tumor. Intra-tumor mutational and copy number heterogeneity was found including intra-tumor diversity at chromosome 13q and 51% somatic mutations defined as unique to one of the three regions of the tumor. This heterogeneity suggests that determining therapy of LPS (personalized medicine) based on a single tumor biopsy may not provide a complete mutational landscape and multi-regional tumor biopsies may be needed to assess the genomic complexity and its clinical impact on the patient.

Many of the genetic alterations that we identified will alter important signaling pathways (e.g., MAPK, ErbB, p53, DNA repair and replication, cell cycle and axon guidance) (Supplementary Table S9). Genes which are therapeutic targets include *MDM2*, *CDK4*, *NF1*, *BRCA1*, *EGFR*, *PI3K*, *AKT1*, *KIT*, *BRAF*, *ERBB2* and *ERBB3*. Also, several important unexplored targets were identified. This work represents a global genomic analysis of LPS cohort and provides insights into the development of novel therapeutic strategies based on molecular phenotype of the tumor.

## MATERIALS AND METHODS

### Patients' samples and cell lines

Primary human LPS tumor tissues and matched normal fat tissues of different histotypes were collected by collaborating with different hospitals [National University Hospital Tissue Repository (Singapore), University of California (Los Angeles, CA, USA), Tumor Repository at Yale University School of Medicine (New Haven, CT, USA), City of Hope Hospital (Duarte, CA, USA) and Oslo University Hospital Sarcoma Biobank (Oslo, Norway)]. Detailed clinical characteristics of all patients are provided in the Supplemental Table S1. Patients used for integrated genomic analyses: SNP array (86 tumors), Discovery cohort (12 tumor-normal pairs) for WES, and Prevalence cohort (14 tumor-normal pairs and 72 tumors) for TES.

Thirteen human LPS cell lines were used in the present study: SW872 (undifferentiated LPS) was purchased from American Tissue Type Culture Collection (ATCC, Rockville, MD, USA); LP6 cells were provided by Dr Christopher DM Fletcher; SA-4 (liposarcoma) cell was a kind gift from Ola Myklebost; LiSa-2 (metastatic poorly differentiated liposarcoma) [32] was kindly provided by Dr. Moller; FU-DDLS-1 [33] and LPS141 [34] (DDLPS) were gifts from Dr. Nishio and Dr. Fletcher, respectively. GOT-3 (recurrence of a myxoid variant of a WDLPS) [35] and MLS-402 (MLPS) [36] were generous gifts from Dr. Åman. T778 and T1000 (recurrent WDLPS) were kind gifts from Dr. Pedeutour. LPS1, LPS2, LPS3 (DDLPS) cell lines were provided by Dr Hong Wu [37]. All these LPS cell lines were maintained in RPMI medium supplemented with fetal bovine serum in a humidified incubator at 37°C with 5% CO_2_[38].

### Genomic DNA and RNA extraction

Genomic DNA (gDNA) was extracted from tumor tissues and cell lines using a QIAamp DNA Blood Mini Kit (Qiagen) according to manufacturer's instructions. The Qubit dsDNA BR Assay Kit (Life technologies) was used to quantify the concentration of gDNA and also 0.7% agarose gel was run for quality of each gDNA sample. Total RNA was isolated using RNeasy Mini Kit (Qiagen) following kit's instructions and quality and concentration of RNA was checked on Bioanalyzer 2100 (version A.02 S1292, Agilent Technologies).

### SNP array analysis

SNP array analysis was performed using GeneChip human mapping SNP array (Affymetrix, Santa Clara, CA, USA). gDNA from 86 LPS tumor samples and 13 cell lines were hybridized to Affymetrix 250K Nsp SNP array and CytoScan SNP array according to instructions provided by array manufacturers [39]. GeneChip Fluidics Station 400 and GeneChip scanner 3000 were used to produce raw data, which were processed and analyzed by copy number analyzer for Affymetrix GeneChip (CNAG 3.0) using copy number analysis as described previously [40, 41]. Significantly recurrent copy number changes were identified using the GISTIC2.0 algorithm [42].

### WES and TES sequencing

For WES, gDNA libraries were prepared using the SureSelect^®^ Human All Exon 50M Kit V4 (Agilent Technologies) as described previously [43]. Captured DNA libraries were subjected to high through-put sequencing using Hiseq2000 Illumina platform with 75 to 100 bp paired-end reads. TES included 248 genes in the customized target enrichment kit and targeted gene libraries were prepared with SureSelectXT2 Target Enrichment System for Illumina Multiplexed Sequencing (Agilent Technologies) according to manufacturer's protocols. Captured libraries were subjected to massively parallel sequencing using HiSeq2000 platform (Illumina).

### Next-generation sequencing data processing and mutation calling

Somatic mutations and short insertions and deletions (Indels) from WES were detected using in-house analysis pipeline as described previously [43-46]. Massively parallel sequencing reads were first aligned to hg19 using Burrows-Wheeler Aligner (V 0.5.8) with default parameters. Low quality reads were filtered, including more than 5 mismatches to the reference sequences or whose mapping quality was less than 30 for summarizing base call data. The significance of each candidate single nucleotide variant (SNV) was evaluated by Fisher's exact test by enumerating the number of the reference bases and the candidate SNV. Finally, a list of candidate somatic mutations was generated by excluding synonymous SNVs and other variants registered in dbSNP131, 1000 genomes, or our in-house SNP database constructed from 180 samples [43, 44]. All the somatic mutations were validated by Sanger sequencing. To detect probable somatic mutations of non-­paired LPS patient samples (no germ line control DNA) and LPS cell lines, besides all the above ­mentioned criteria applied, we further removed copy number-neutral variants with allele frequency between 45% and 55% unless they were registered in the COSMIC data base.

### Validation by sanger sequencing

All mutations and Indels found by next generation sequencing were validated with Sanger sequencing. PCR primers were designed for the putative variants using Primer3 and were used to amplify the source DNA from the tumors and germline control. Products were sequenced; and sequences were analyzed with Sequencing Analysis Software Version 5.2 (Applied Biosystems).

### Analysis of significantly mutated genes and pathways

Validated list of SNVs and Indels across the cohort were used to identify significantly altered genes. We performed ‘Significantly Mutated Genes tests (SMG-test) from ‘Mutational Significance in Cancer’ (MuSiC) tool to identify such genes [24]. Each mutation is categorized into seven categories, AT transitions, AT transversions, CpG transitions, CpG transversions, CG transitions/transversions and Indels. Final Background Mutation Rate (BMR) is estimated by dividing number of mutations found in each category and total number of available bases. A gene is identified as significantly mutated, if its mutation rate is significantly higher than the estimated BMR. The refseq annotated mutations (genes) were used for pathway analysis. Pathway analysis was done using PathScan algorithm integrated into MuSic pipeline [47].

### Analysis of mutational signature

We used EMu, a probabilistic method which uses Expectation Maximization (EM) algorithm to identify Mutational Signatures. EMu also measures the tumor specific “opportunity” from CNAs to measure the “opportunity” for a given variant to occur in that region thus increasing the accuracy of inferred Signature. Along with the validated variants, we also used all high quality tier1 and tier2 variants from WES samples for Signature Analysis [48]. Before running Emu, mutation opportunity was calculated using CNAs in tumor. Briefly, read depths for every 100 base pair segments were estimated for tumor and matched normal samples, and depth ratio was calculated using VarScan2 copy number programme [49]. These segments were later delineated by Circular Binary Segmentation algorithm to detect potential copy number altered regions [50]. This segmented data was fed into Emu along with the mutations classified into 96 trinucleotide channels based on the bases immediate to the 5′ and 3′ of the mutated site.

### RT-PCR and quantitative real-time PCR analysis

Total RNA was reverse transcribed to cDNA with RevertAid First Strand cDNA Synthesis Kit (Thermo Scientific). Quantitative gene expression levels were detected using KAPA-SYBR Green RT-PCR with the ABI PRISM 7500 Fast Sequence Detection System (Applied Biosystems). Expression levels of target genes were normal­ized to *GAPDH* mRNA levels. Primer sequences are provided in Supplementary Table 10.

### Genomic quantitative PCR

gDNA of LPS patient samples was quantified and diluted at a concentration of 25 ng/ml. For reaction, 2X SYBR Green PCR master mix, forward and reverse primers specific for *CPM* were added and pipetted into respective wells of a PCR plate. gDNA (1 ul) was added in each well and run in ABI PRISM 7500 Fast Sequence Detection System (Applied Biosystems) at following conditions; 50°C for 2 min, denaturation at 95°C for 10 min, followed by 40 cycles of denaturing at 95°C for 15 s and combined annealing and extension at 60°C for 60 s. *GAPDH* specific primers were used as control.

### Immunohistochemistry

Tissue microarray slides were purchased from Super Bio Chips which were deparaffinized and hydrated in xylene and graded ethanol to distilled water. A blocking step to quench endogeneous peroxidase was performed in 0.3% H_2_O_2_ in 95% ethanol for 5 min and antigen retrieval was done with a retrieval buffer (pH 6). Slides were blocked and incubated with CPM antibody for 30 min followed by washing. Secondary rabbit-horse radish peroxidase labelled antibody was added, followed by washing and developed with DAB solution. Counterstaining was done with Mayers hematoxylin for 5 min and slides were mounted and was scanned using the automated scanning system Aperio XT (Aperio Technologies).

### Flow cytometry

For surface staining, cells were harvested, washed (single cell suspension) and resuspended (1-5 × 10^6^ cells/ml) in ice cold FACS Buffer (PBS, 2% FBS). Cells were blocked for 20 min and incubated with CPM antibody (1:100 dilution) for 30 min on ice. After washing with ice-cold FACS buffer, cells were subjected to secondary labeled antibody (1:500 dilution), incubated for 30 min in the dark on ice, washed three times and analyzed on flow cytometer.

For cell cycle analysis, cells were harvested, washed with ice-cold PBS and fixed using ice-cold 70% ethanol. Propidium iodide/RNase solution was added to cells on ice and analyzed by flow cytometer. Apoptosis was detected by flow cytometry using the Annexin V-FITC Apoptosis Detection kit (BD Pharmingen) according to the manufacturer's instructions.

### Cell proliferation assay

Cells were seeded (5000 cells/well) in 96-well plates and MTT (3-(4, 5-dimethylthiazol-2-yl)-2,5-diphenyl tetrazolium bromide) assay was performed at 24 h, 48 h, 72 h and 96 h according to standard protocol. Cells were incubated with MTT (0.5 mg/ml) for 2-4 h in a 37°C CO_2_ incubator. Formazan crystals were dissolved in 100 ul of MTT stop solution (SDS-HCl). Absorbance was measured at 570 nm using a Tecan Infinite 200 PRO spectrophotometer (Mannedorf, Switzerland).

### Colony formation assay

For anchorage-dependent colony assay, cells were seeded in 6-well plates (500 cells/well) and allowed to grow for 10-15 days to form colonies. Colonies were washed with PBS, fixed with methanol and stained with crystal violet. For anchorage-independent colony assays, cells were plated in 0.5% agarose on top of 1% agarose in 24-well plates. Cells were allowed to grow for 2-3 weeks to form colonies; and colonies were counted under an inverted microscope.

### Migration and invasion assay

Cells in serum-free medium were seeded in Transwell inserts (Corning) for migration assay and in Matrigel coated Transwell inserts for invasion assay in 24-well plates. Cells were incubated with serum containing medium in the lower chamber. After 24 h, inserts were washed and non-migrating/non-invading cells were removed and migrated/invaded cells were fixed with methanol and stained with crystal violet.

### Immunoblot analysis

Cell lysates were prepared using Protein Extraction reagent (Thermo Scientific) containing protease inhibitor cocktail and subjected to SDS-PAGE. Proteins were transferred to polyvinyidene difluoride membranes which were further incubated with the indicated antibodies and detection was performed using Chemiluminescent HRP Substrate (Thermo Scientific). List of antibodies used are provided in Supplementary Table 11.

### Xenograft assay

Male NOD SCID gamma mice (5-6 weeks old) were used for xenograft assay in compliance with ethical regulations of Institutional Animal Care and Use Committee of National University of Singapore. *CPM* shRNA3 knockdown LPS141 cells and non-target shRNA LPS141 cells (2 × 10^6^cells per mice) mixed with matrigel were injected subcutaneously in the right and left flank of the mice, respectively. After 4-6 weeks of injection, mice were eutha­nized to weigh and analyze the dissected tumors.

### RNA interference

Human *CPM* gene specific siRNA duplexes including control scramble siRNA duplexes were purchased from Integrated DNA Technologies (Coralville, USA). LPS141, FU-DDLS-1 and SW872 cells were transfected with 100nM siRNA duplex using Nucleofector Kit (Lonza, Cologne, Germany) according to the manufacturer's protocol. 2 ug of pmaxGFP vector was used to ensure transfection efficiency. The average transfection efficiency was approximately 60-70%. Cells were incubated for at least 48 h after nucleofection before performing experiments. Each experiment was performed at least in triplicate on three different occasions.

To obtain stable knockdown of *CPM* and *NF1* in LPS cells for *in vitro* and *in vivo* studies, human *CPM* and *NF1* gene specific shRNAs and a non-targeting shRNA were cloned in a lentiviral vector. For stable knockdown, lentiviral particles were generated according to the manufacturer's protocol. Cells were infected with lentivirus particles at a MOI of 25 with 5 ug/ml polybrene (Sigma-Aldrich) for 24 h and stable cells were selected using 0.5-1 ug/ml puromycin for 2 weeks.

### Statistical analyses

Two-tailed Student's *t*-test was used for the following assays: quantitative real-time PCR, cell proliferation assay, colony formation assay, cell cycle analysis, apoptosis assay, migration assay, invasion assay and xenograft growth assay.

### Study approval

Informed written consent was obtained from all the patients for the sample collection and their use in research and projects were approved by Ethical Review Boards.

## SUPPLEMENTARY MATERIAL FIGURES AND TABLES


