# Supplementary material for: Genomic landscape of liposarcoma
Source: Oncotarget. 2015 Dec 4;6(40):42429–44. doi: 10.18632/oncotarget.6464 (PMC4767443; doi:10.18632/oncotarget.6464)
Supplement: Supplementary file 1 [file oncotarget-06-42429-s001.pdf]

Genomic landscape of liposarcoma

Supplementary Material

Supplementary Figure S1. Profiling of *CPM* expression in LPS patient samples and LPS cell lines.

Supplementary Figure S2. Role of *CPM* in LPS.

Supplementary Figure S3. Mean coverage of WES in 12 LPS patient tumor samples with the matching control normal samples in Discovery cohort at ≥ 20X.

Supplementary Figure S4. Mutation frequencies and significantly mutated genes in Prevalence Cohort.

Supplementary Figure S5. Alterations of frequently mutated genes *NF1* and *BRAF*

Supplementary Table S1: Patient sample's clinical details and analyses performed.

Supplementary Table S2: Regions of significant amplifications and deletions identified using GISTIC analysis in LPS patient samples.

Supplementary Table S3: Regions of significant amplifications and deletions identified using GISTIC analysis in LPS cell lines.

Supplementary Table S4: Coverage analysis of whole exome sequencing of 12 LPSs and matched adjacent normal tissues in Discovery Cohort.

Supplementary Table S5: Summary of somatic mutations in the Discovery Cohort.

Supplementary Table S6: List of genes for targeted capture in Prevalence cohort study.

Supplementary Table S7: Coverage analysis of targeted exome sequencing of 90 LPSs in Prevalence cohort.

Supplementary Table S8: Summary of mutations in the LPS Prevalence Cohort and LPS cell lines.

Supplementary Table S9: List of significantly mutated genes identified by MuSiC analysis.

Supplementary Table S10: KEGG pathway analysis.

Supplementary Table S11: List of primer sequences.

Supplementary Table S12: List of antibodies used in the study.

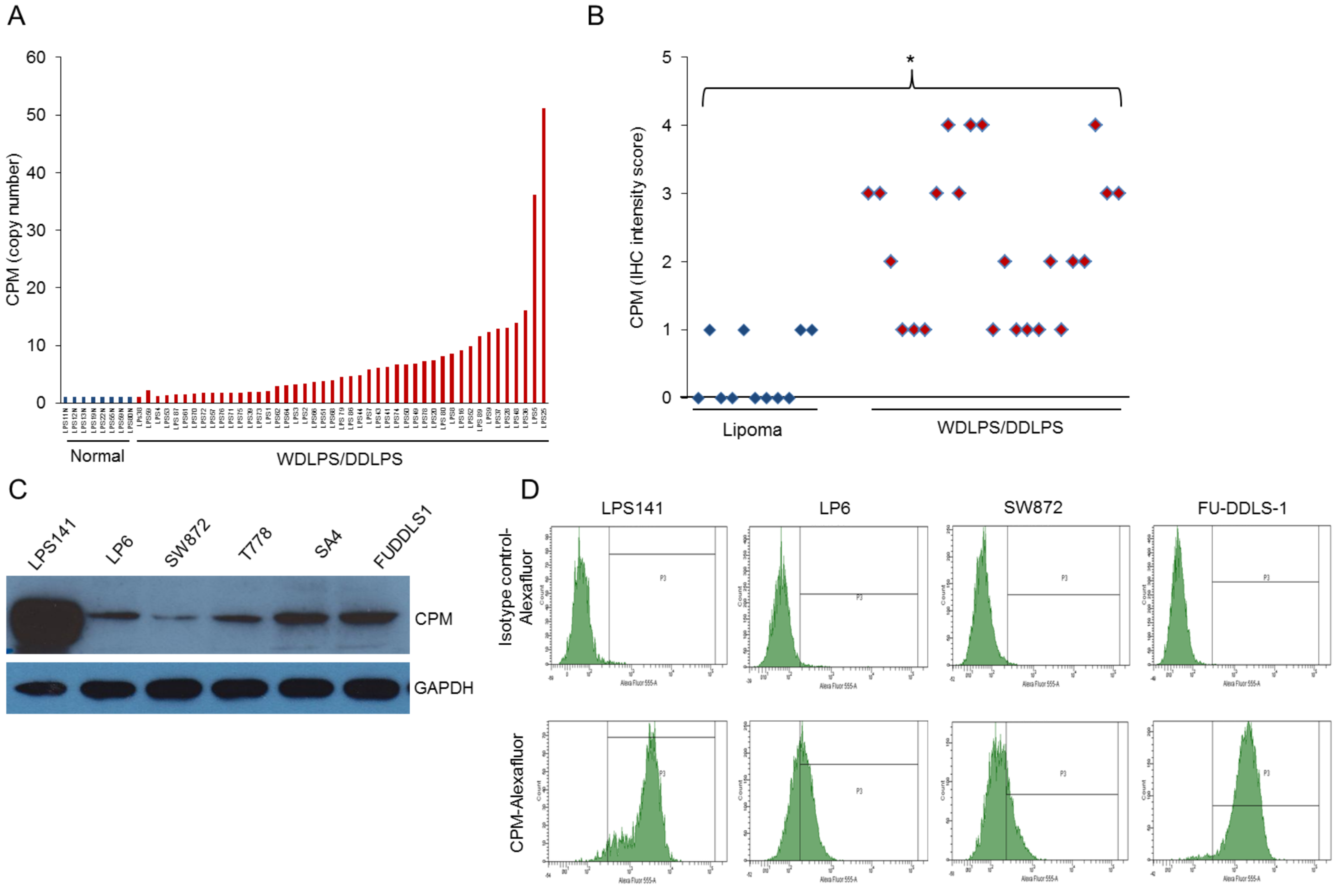

Supplementary Figure S1: Profiling of *CPM* expression in LPS patient samples and LPS cell lines. A. Copy number of *CPM* gene in LPS patient samples determined by genomic quantitative PCR. Copy number change compared with the normal control samples shown in WDLPS/DDLPS cases. **B.** Distribution of CPM IHC staining scores in lipoma and LPS tissues. CPM is expressed higher in LPS tissues than in benign lipoma. **C.** Western blotting analysis of CPM protein expression in LPS cell lines [GAPDH, internal loading control]. **D.** Flow cytometric analysis of CPM surface expression in LPS cell lines.

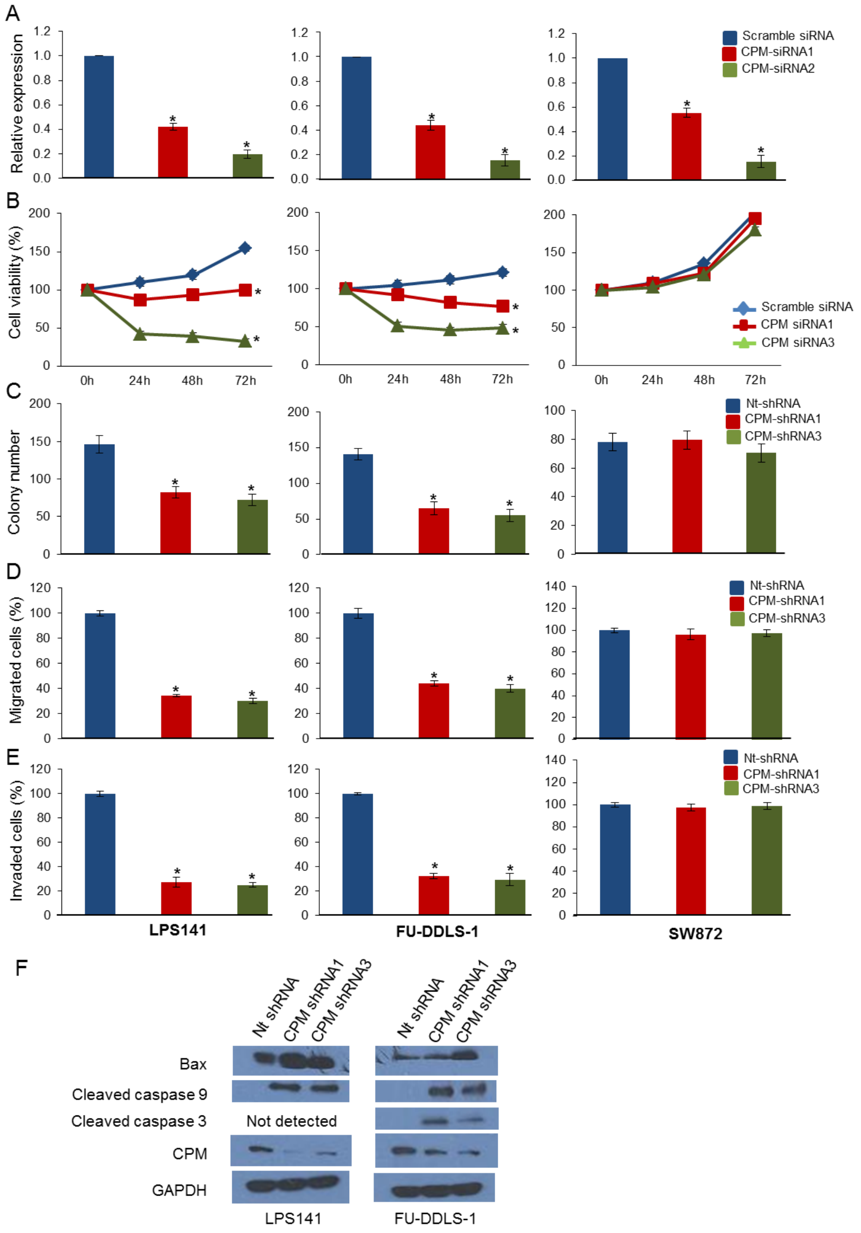

Supplementary Figure S2: Role of *CPM* in LPS. A. Quantitative real time PCR analysis: *CPM* expression levels in LPS141, FU-DDLS-1 and SW872 cells with *CPM* knockdown using siRNA1 and siRNA2 relative to scramble siRNA. **B**. Cell viability MTT assay: Effect of *CPM* siRNA on cell viability at 24 h, 48 h and 72 h, relative to scramble siRNA. Cells were transiently transfected with *CPM* siRNA1 and siRNA2 vs. control scramble siRNA, seeded in 96-well plates (5×10^3 per well) and grown for 3 days followed by MTT assay. **C.** Soft agar colony assay: Effect on clonogenic property of stable CPM knockdown cells using shRNA1 and shRNA3 compared to control Nt-shRNA transduced cells. (**D**) Migration assay: Number of migrated cells measured using Transwell assay of CPM shRNA and Nt-shRNA infected LPS cells. CPM knockdown significantly decreases the migration ability of LPS141 and FU-DDLS-1 cells. **E.** Invasion assay: Quantification of the numbers of CPM shRNA and

Nt-shRNA LPS cells migrating through Matrigel in Transwell invasion assay. **F.** Western blot analysis for the expression of apoptosis associated proteins in CPM knockdown LPS141 and FU-DDLS-1 cells. Data represents mean ± S.E.; n = 3. *$P < 0.05$ [Note: Value of S.E. are too small to be visible in the panel **B**]

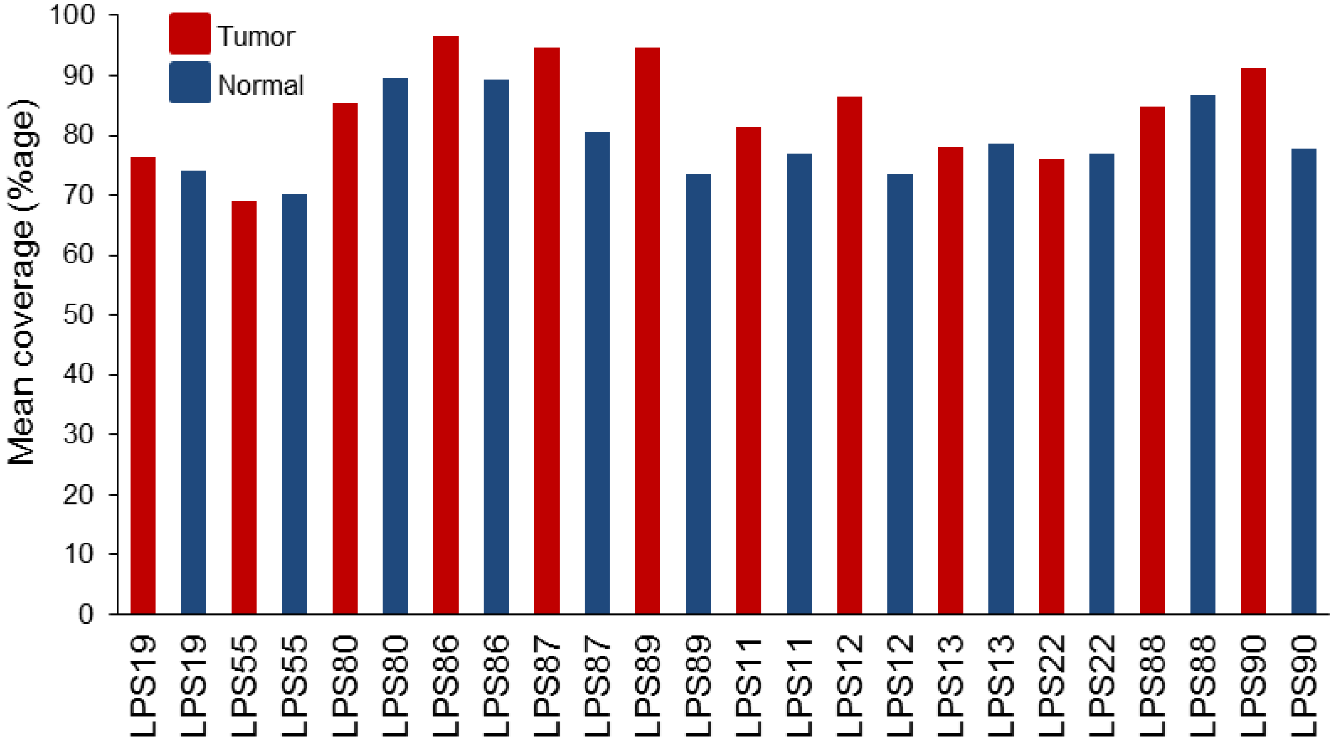

Supplementary Figure S3: Mean coverage of WES in 12 LPS patient tumor samples with the matching normal control samples in Discovery cohort at ≥ 20X. Coverage of the coding regions is plotted for each pair of LPS patient sample.

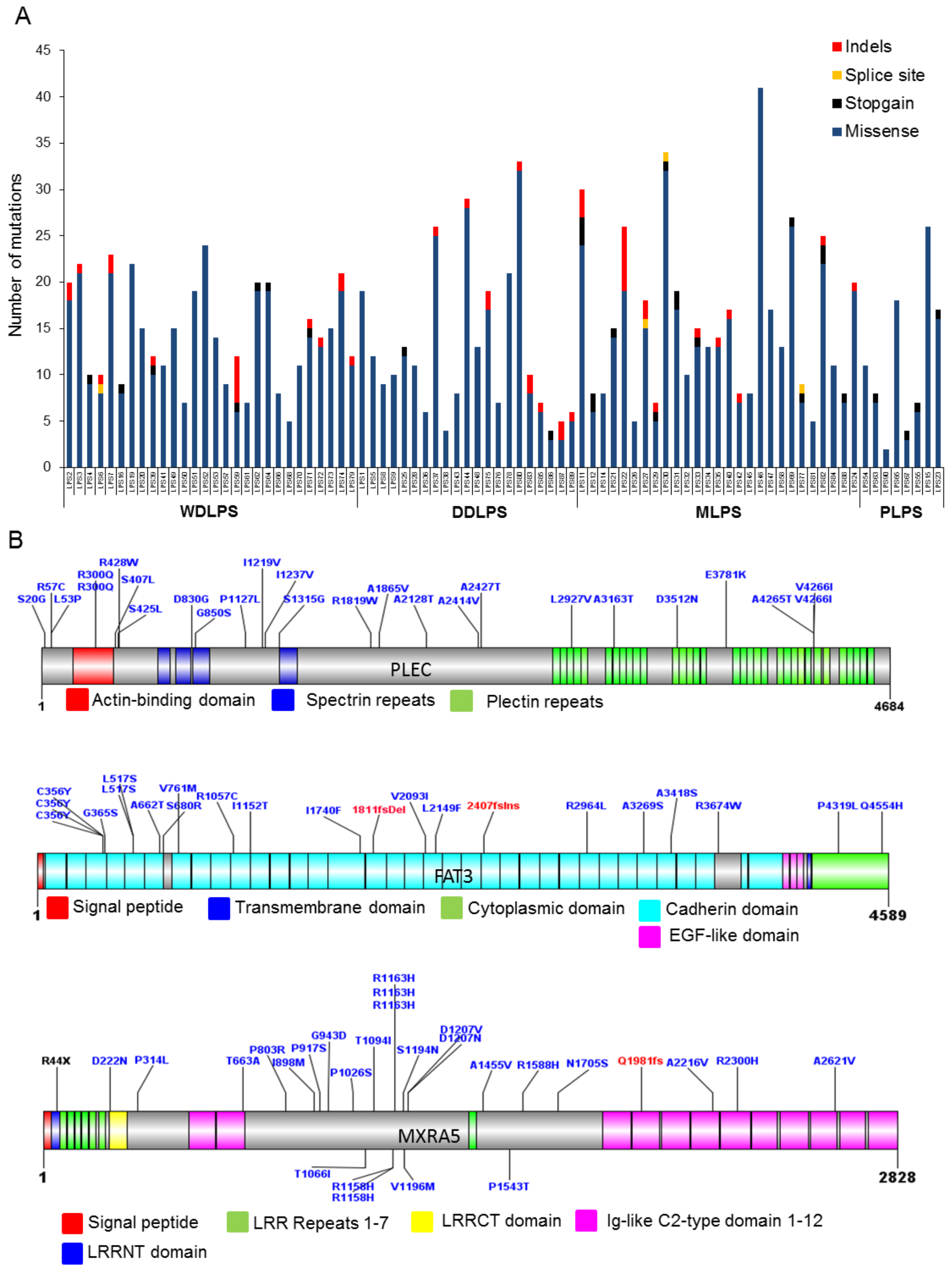

Supplementary Figure S4: Mutation frequencies and significantly mutated genes in Prevalence Cohort. A. Number and distribution of mutations identified in each patient sample of the Prevalence cohort. **B.** Distribution of validated mutations found in significantly mutated recurrent genes; *PLEC*, a cytoskeletal-linker protein which plays an important role in maintaining the integrity of tissue structure found to be most highly recurrently mutated gene in 27% of LPS patients; *FAT3*, a member of FAT family, cadherins involved in tumor suppression and planar cell polarity and is the second most frequently (24%) mutated. *MXRA5*, a matrix-remodeling associated protein involved in ECM remodeling and cell-cell adhesion was mutated in 27% of patients.

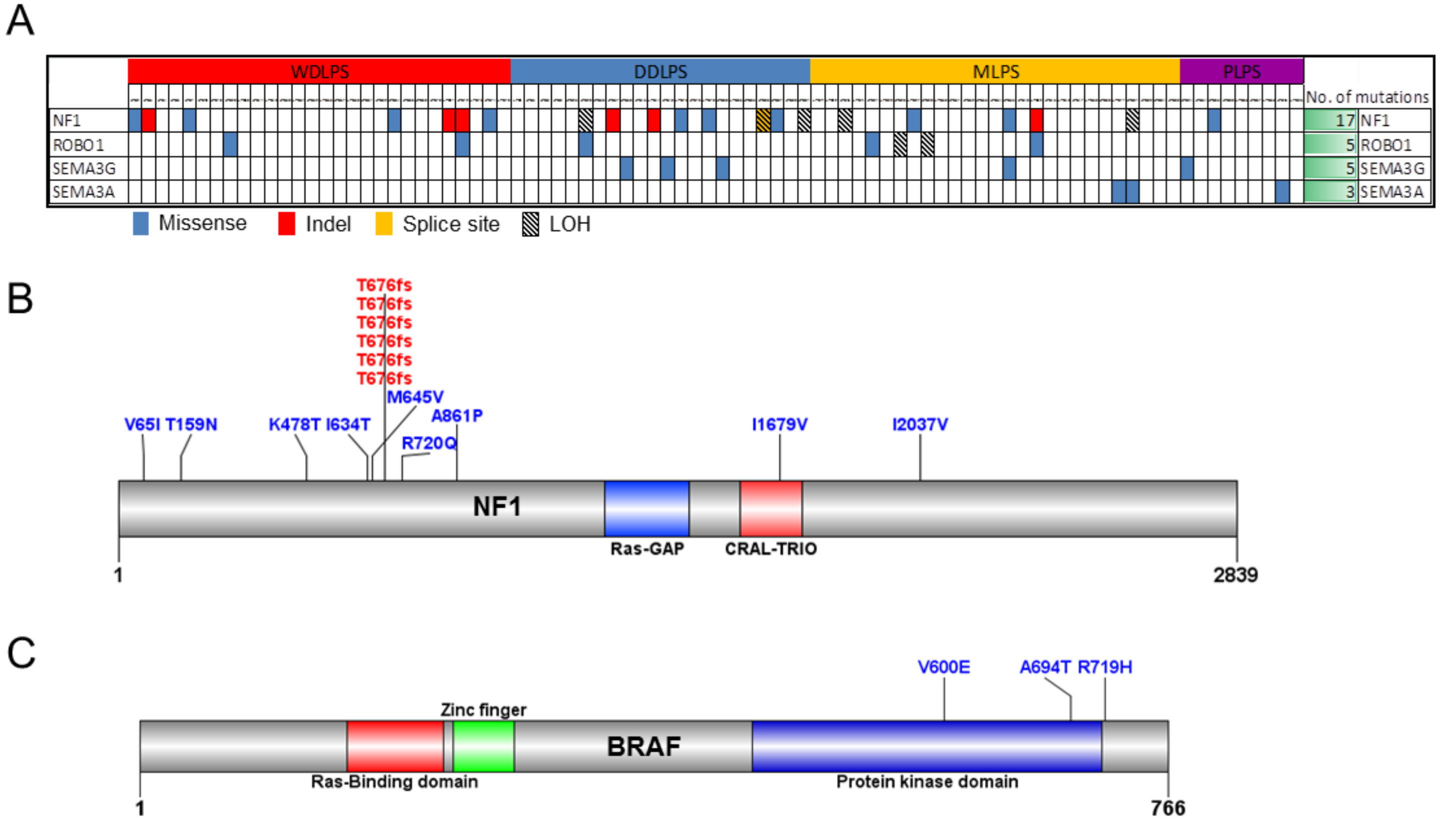

Supplementary Figure S5. Alterations of frequently mutated genes *NF1* and *BRAF*. A. Number and type of alterations in class of genes with regulatory role in axon guidance and cancer cell growth in LPS Prevalence cohort. **B.** Distribution of validated mutations along the length of *NF1* gene. **C.** Distribution of validated mutations found in the *BRAF* gene in patient samples and cell lines.

Supplementary Table S1: Patient sample's clinical details and analyses performed

	Sample ID	Race<br>W:Caucasian<br>B: African<br>A: Asian	Gender<br>M: Male<br>F: Female	Age	LPS<br>DD: de-differentiated LPS<br>WD: well-differentiated LPS<br>M: myxoid LPS<br>P: Pleomorphic LPS	Site	Survival		SNP Chips tumor	Whole Exome Sequencing	Targeted-Exome Sequencing
							Follow-up	Vital Status			
Discovery Cohort	LPS11	W	F	80	M	NA	128	alive	Affymetrix 250K Nsp	SureSelect 50M	SureSelect customized beads
	LPS12	W	F	50	M	NA	125	dead	Affymetrix 250K Nsp	SureSelect 50M	SureSelect customized beads
	LPS13	W	F	52	M	NA	93	alive	Affymetrix 250K Nsp	SureSelect 50M	X
	LPS19	W	F	84	WD	NA	64	dead	Affymetrix 250K Nsp	SureSelect 50M	SureSelect customized beads
	LPS22	W	M	48	M	NA	51	dead	Affymetrix 250K Nsp	SureSelect 50M	SureSelect customized beads
	LPS55	A	F	54	WD	Soft tissue	73	dead	Affymetrix 250K Nsp	SureSelect 50M	SureSelect customized beads
	LPS80	W	F	56	DD	Retroperitoneal space	NA	Dead	Affymetrix 250K Nsp	SureSelect 50M	SureSelect customized beads
	LPS86	W	F	53	DD	NA	NA	NA	Affymetrix CytoScan	SureSelect 50M	SureSelect customized beads
	LPS87	W	M	51	DD	NA	NA	NA	Affymetrix CytoScan	SureSelect 50M	SureSelect customized beads
	LPS88	W	F	60	M	NA	NA	NA	Affymetrix CytoScan	SureSelect 50M	SureSelect customized beads
	LPS89	W	F	67	DD	NA	NA	NA	Affymetrix CytoScan	SureSelect 50M	SureSelect customized beads
	LPS90	W	M	55	P	NA	NA	NA	Affymetrix CytoScan	SureSelect 50M	SureSelect customized beads
Prevalence Cohort	LPS1	NA	M	75	DD	NA	NA	NA	Affymetrix 250K Nsp	X	SureSelect customized beads
	LPS2	NA	M	72	WD	NA	NA	NA	Affymetrix 250K Nsp	X	SureSelect customized beads
	LPS3	NA	NA	NA	WD	NA	NA	NA	Affymetrix 250K Nsp	X	SureSelect customized beads
	LPS4	NA	F	53	WD	NA	NA	NA	Affymetrix 250K Nsp	X	SureSelect customized beads
	LPS5	NA	F	67	DD	NA	NA	NA	Affymetrix 250K Nsp	X	SureSelect customized beads
	LPS6	NA	NA	NA	WD	NA	NA	NA	Affymetrix 250K Nsp	X	SureSelect customized beads
	LPS7	NA	NA	NA	WD	NA	NA	NA	Affymetrix 250K Nsp	X	SureSelect customized beads
	LPS8	NA	M	43	DD	NA	NA	NA	Affymetrix 250K Nsp	X	SureSelect customized beads
	LPS9	NA	NA	NA	DD	NA	NA	NA	Affymetrix 250K Nsp	X	SureSelect customized beads
	LPS14	W	M	35	M	NA	125	alive	Affymetrix 250K Nsp	X	SureSelect customized beads
	LPS15	W	M	68	NOS	NA	15	alive	Affymetrix 250K Nsp	X	SureSelect customized beads
	LPS16	W	M	75	WD	NA	21	alive	Affymetrix 250K Nsp	X	SureSelect customized beads
	LPS20	W	F	78	WD	NA	69	alive	Affymetrix 250K Nsp	X	SureSelect customized beads
	LPS21	W	F	47	M	NA	100	dead	Affymetrix 250K Nsp	X	SureSelect customized beads
	LPS23	W	M	71	NOS	NA	63	dead	Affymetrix 250K Nsp	X	SureSelect customized beads
	LPS24	W	M	76	P	NA	19	dead	Affymetrix 250K Nsp	X	SureSelect customized beads
	LPS25	W	M	72	DD	Thigh	41	dead	Affymetrix 250K Nsp	X	SureSelect customized beads
	LPS26	W	M	47	M	Soft tissue	76	dead	Affymetrix 250K Nsp	X	SureSelect customized beads
	LPS27	W	F	32	M	Retroperitoneum	13	dead	Affymetrix 250K Nsp	X	SureSelect customized beads
	LPS28	W	F	33	DD	Pelvis	1	dead	Affymetrix 250K Nsp	X	SureSelect customized beads
	LPS29	W	F	14	M	Thigh	135	alive	Affymetrix 250K Nsp	X	SureSelect customized beads
	LPS30	W	F	46	M	Pelvis	36	dead	Affymetrix 250K Nsp	X	SureSelect customized beads
	LPS31	A	F	45	M	Thigh	10	dead	Affymetrix 250K Nsp	X	SureSelect customized beads
	LPS32	W	M	74	M	Thigh	125	alive	Affymetrix 250K Nsp	X	SureSelect customized beads
	LPS33	W	M	57	M	Soft tissue	64	dead	Affymetrix 250K Nsp	X	SureSelect customized beads
	LPS34	W	M	66	M	Retroperitoneum	42	dead	Affymetrix 250K Nsp	X	SureSelect customized beads
	LPS35	W	F	50	M	Thigh	60	alive	Affymetrix 250K Nsp	X	SureSelect customized beads
	LPS36	W	M	83	DD	Testicle	64	dead	Affymetrix 250K Nsp	X	SureSelect customized beads
	LPS37	W	M	57	DD	Retroperitoneum	71	alive	Affymetrix 250K Nsp	X	SureSelect customized beads
	LPS38	W	M	70	DD	Retroperitoneum	9	dead	Affymetrix 250K Nsp	X	SureSelect customized beads
	LPS39	W	F	57	WD	Retroperitoneum	NA	alive	Affymetrix 250K Nsp	X	SureSelect customized beads
	LPS40	W	M	46	M	Soft tissue	1	alive	Affymetrix 250K Nsp	X	SureSelect customized beads
	LPS41	W	F	52	WD	Thigh	34	alive	Affymetrix 250K Nsp	X	SureSelect customized beads
	LPS42	W	M	85	M	Groin	31	alive	Affymetrix 250K Nsp	X	SureSelect customized beads
	LPS43	W	F	49	DD	Pelvis	1	alive	Affymetrix 250K Nsp	X	SureSelect customized beads
	LPS44	W	M	68	DD	Shoulder	19	alive	Affymetrix 250K Nsp	X	SureSelect customized beads
	LPS45	W	F	69	M	Thigh	30	alive	Affymetrix 250K Nsp	X	SureSelect customized beads
	LPS46	B	M	36	M	Foot	2	alive	Affymetrix 250K Nsp	X	SureSelect customized beads
	LPS47	A	F	69	M	Pelvis	1	alive	Affymetrix 250K Nsp	X	SureSelect customized beads
	LPS48	W	M	51	DD	Retroperitoneum	17	alive	Affymetrix 250K Nsp	X	SureSelect customized beads
	LPS49	W	M	55	WD	Shoulder	1	alive	Affymetrix 250K Nsp	X	SureSelect customized beads
	LPS50	W	M	74	WD	Thigh	1	alive	Affymetrix 250K Nsp	X	SureSelect customized beads
	LPS51	W	F	60	WD	Retroperitoneum	4	alive	Affymetrix 250K Nsp	X	SureSelect customized beads
	LPS52	W	M	55	WD	Leg	1	alive	Affymetrix 250K Nsp	X	SureSelect customized beads
	LPS53	A	M	47	WD	Soft tissue	1	alive	Affymetrix 250K Nsp	X	SureSelect customized beads
	LPS54	A	F	50	P	Soft tissue	62	dead	Affymetrix 250K Nsp	X	SureSelect customized beads

	LPS57	A	M	57	WD	Pelvis	119	alive	Affymetrix 250K Nsp	X	SureSelect customized beads
	LPS58	A	F	33	M	Thigh	61	alive	Affymetrix 250K Nsp	X	SureSelect customized beads
	LPS59	A	F	45	WD	Thigh	51	alive	Affymetrix 250K Nsp	X	SureSelect customized beads
	LPS61	A	M	63	WD	Thigh	42	alive	Affymetrix 250K Nsp	X	SureSelect customized beads
	LPS62	A	F	29	WD	Shoulder	47	alive	Affymetrix 250K Nsp	X	SureSelect customized beads
	LPS63	A	F	62	P	Peritoneum	2	alive	Affymetrix 250K Nsp	X	SureSelect customized beads
	LPS64	A	M	76	WD	Thigh	36	alive	Affymetrix 250K Nsp	X	SureSelect customized beads
	LPS65	A	M	61	Mxd	Retroperitoneum	38	alive	Affymetrix 250K Nsp	X	SureSelect customized beads
	LPS66	A	F	72	WD	Thigh	30	alive	Affymetrix 250K Nsp	X	SureSelect customized beads
	LPS67	A	M	45	Mxd	Thigh	35	alive	Affymetrix 250K Nsp	X	SureSelect customized beads
	LPS68	A	M	41	WD	Thigh	27	alive	Affymetrix 250K Nsp	X	SureSelect customized beads
	LPS69	A	M	57	M	Retroperitoneum	46	alive	Affymetrix 250K Nsp	X	SureSelect customized beads
	LPS70	A	M	86	WD	Retroperitoneum	14	alive	Affymetrix 250K Nsp	X	SureSelect customized beads
	LPS71	A	F	41	WD	Thigh	19	alive	Affymetrix 250K Nsp	X	SureSelect customized beads
	LPS72	A	F	73	WD	Retroperitoneum	21	alive	Affymetrix 250K Nsp	X	SureSelect customized beads
	LPS73	A	M	63	WD	Thigh	0	alive	Affymetrix 250K Nsp	X	SureSelect customized beads
	LPS74	A	M	57	WD	Retroperitoneum	119	alive	Affymetrix 250K Nsp	X	SureSelect customized beads
	LPS75	A	M	52	DD	Retroperitoneum	4	alive	Affymetrix 250K Nsp	X	SureSelect customized beads
	LPS76	W	NA	NA	DD	NA	NA	NA	Not Done	X	SureSelect customized beads
	LPS77	W	NA	NA	M	NA	NA	NA	Affymetrix 250K Nsp	X	SureSelect customized beads
	LPS78	W	NA	NA	DD	NA	NA	NA	Affymetrix 250K Nsp	X	SureSelect customized beads
	LPS79	W	NA	NA	WD	NA	NA	NA	Affymetrix 250K Nsp	X	SureSelect customized beads
	LPS81	A	M	63	M	Retroperitoneal space	NA	NA	Affymetrix 250K Nsp	X	SureSelect customized beads
	LPS82	A	M	29	M	Right thigh	NA	NA	Affymetrix 250K Nsp	X	SureSelect customized beads
	LPS83	W	NA	NA	DD	NA	NA	NA	Affymetrix 250K Nsp	X	SureSelect customized beads
	LPS84	W	NA	NA	M	NA	NA	NA	Affymetrix 250K Nsp	X	SureSelect customized beads
	LPS85	W	NA	NA	DD	NA	NA	NA	Affymetrix 250K Nsp	X	SureSelect customized beads
ITH	LPS91-T1	W	NA	NA	DD	NA	NA	NA	Affymetrix CytoScan	SureSelect 50M	SureSelect customized beads
	LPS91-T2	W	NA	NA	DD	NA	NA	NA	Affymetrix CytoScan	SureSelect 50M	SureSelect customized beads
	LPS91-T3	W	NA	NA	DD	NA	NA	NA	Affymetrix CytoScan	SureSelect 50M	SureSelect customized beads
Cell Lines	FU-DDLS-1		M		DD				Affymetrix CytoScan	X	SureSelect customized beads
	GOT-3		F		M				Affymetrix CytoScan	X	SureSelect customized beads
	LiSa-2		M		P				Affymetrix CytoScan	X	SureSelect customized beads
	LPS141		M		DD				Affymetrix CytoScan	X	SureSelect customized beads
	LP1		M		DD				Affymetrix CytoScan	X	SureSelect customized beads
	LP2		F		DD				Affymetrix CytoScan	X	SureSelect customized beads
	LP3		M		DD				Affymetrix CytoScan	X	SureSelect customized beads
	LP6		M		DD				Affymetrix CytoScan	X	SureSelect customized beads
	SA4		M		DD				Affymetrix CytoScan	X	SureSelect customized beads
	MLS402		M		M				Affymetrix CytoScan	X	SureSelect customized beads
	T1000		M		WD				Affymetrix CytoScan	X	SureSelect customized beads
	T778		F		WD				Affymetrix CytoScan	X	SureSelect customized beads
	SW872		M		WD				Affymetrix CytoScan	X	SureSelect customized beads

Supplementary Table S2: Regions of significant amplifications and deletions identified in LPS patients using GISTIC analysis

Amplifications

Cytoband	q value	Residual q value	Wide peak boundaries	Genes in wide peak
12q14.2	4.99E-76	5.89E-61	chr12:64498285-64542661	SRGAP1
12q13.2	7.90E-63	7.57E-49	chr12:56306404-56586248	CDK2, DGKA, ERBB3, MYL6, PA2G4, RAB5B, RPL41, RPS26, PMEL, SMARCC2, SUOX, ESYT1, IKZF4, WIBG, ZC3H10, MYL6B
12q14.3	3.84E-97	7.14E-47	chr12:67443968-67499750	[CAND1]
12q15	1.02E-93	2.12E-36	chr12:68215288-68328705	[DYRK2]
12q14.3	2.25E-41	3.52E-27	chr12:66195618-66243501	HMGA2, RPSAP52
12q21.33	6.81E-32	2.12E-21	chr12:90113349-90171437	[LOC338758]
12q22	1.17E-27	6.73E-21	chr12:95119817-95206921	[MIR492]
12q14.1	3.63E-31	3.35E-19	chr12:58129741-58203033	CDK4, CYP27B1, METTL1, TSPAN31, TSFM, AVIL, METTL21B, AGAP2
12q14.3	3.40E-33	7.70E-18	chr12:67042639-67165808	GRIP1
12q15	4.70E-58	1.38E-16	chr12:69987495-70082408	CCT2, BEST3, LRRC10
12q21.31	4.34E-35	1.03E-15	chr12:81704065-81810830	PPFIA2
12q21.2	4.85E-27	2.20E-15	chr12:75954692-76007828	[KRR1]
12q21.33	5.53E-22	4.79E-15	chr12:91069106-91155406	[C12orf37]
12q14.1	1.88E-17	7.51E-14	chr12:60536258-60620625	[SLC16A7]
12q21.31	3.04E-26	1.07E-13	chr12:82249068-82330394	[PPFIA2]
12q15	3.83E-59	1.11E-13	chr12:68771885-68847873	[MDM1]
12q21.1	2.54E-24	1.20E-13	chr12:74331424-74351098	[LOC100507377]
12q21.1	2.54E-30	1.63E-13	chr12:72263831-72298334	TBC1D15
12q15	1.96E-60	3.24E-13	chr12:69745544-69815757	LYZ, YEATS4
12q21.31	4.17E-29	1.89E-12	chr12:81298480-81456745	hsa-mir-618, LIN7A, MIR618
12q14.2	3.00E-27	4.44E-12	chr12:64291912-64346945	SRGAP1
12q21.1	3.42E-26	1.63E-11	chr12:73671021-73737493	[TRHDE]
12q21.31	4.00E-22	2.26E-11	chr12:83698399-83717706	[TMTC2]
12q15	1.31E-52	3.09E-11	chr12:69459348-69486990	[CPM]
12q14.1	5.43E-23	5.93E-11	chr12:58817775-58843458	[LRIG3]
12q15	2.49E-31	9.48E-11	chr12:70943933-70965478	PTPRB
12q21.2	2.73E-25	1.13E-10	chr12:79736117-79762917	SYT1
12q21.32	1.15E-25	3.26E-10	chr12:88393803-88413151	C12orf50
12q21.1	1.96E-27	3.92E-10	chr12:72878716-73012572	TRHDE
12q22	7.91E-12	5.52E-10	chr12:92566742-92683169	[BTG1]
12q21.31	1.08E-21	7.05E-10	chr12:82982760-83080678	[TMTC2]
12q15	1.33E-26	2.96E-09	chr12:71378198-71529490	TSPAN8
12q21.32	2.10E-23	3.14E-09	chr12:88713514-88773481	[KITLG]
12q21.1	3.42E-26	6.65E-09	chr12:73287594-73429578	[TRHDE]
12q14.3	5.89E-22	9.81E-09	chr12:65463648-65504923	WIF1
12q21.33	7.20E-18	5.05E-08	chr12:89535971-89631048	[DUSP6]
12q14.1	9.81E-09	6.43E-08	chr12:62600958-62702550	USP15
12q21.33	1.49E-18	8.12E-08	chr12:90539888-90610080	[LOC338758]
12q21.2	4.49E-21	8.50E-08	chr12:80217967-80294653	PPP1R12A
12q14.3	4.17E-25	1.44E-07	chr12:65075639-65128692	GNS, RASSF3
12q21.31	1.53E-13	1.64E-07	chr12:85145696-85218696	[SLC6A15]
12q14.1	8.80E-09	1.71E-07	chr12:62948831-63172217	hsa-let-7i, MON2, PPM1H, MIRLET7I
12q21.31	8.12E-15	2.03E-07	chr12:86012641-86128296	[RASSF9]
12q13.13	5.95E-09	2.52E-07	chr12:53927486-53984011	ATF7
12q14.2	7.87E-09	2.70E-07	chr12:63666497-63819353	[AVPR1A]
12q21.32	9.43E-22	5.09E-07	chr12:87707563-87827675	[MKRN9P]
12q13.3	1.33E-25	6.04E-07	chr12:57453204-57522995	LRP1, NAB2, STAT6, TMEM194A
12q21.2	2.01E-17	6.36E-07	chr12:76992296-77019877	[OSBPL8]
12q13.2	1.06E-11	6.64E-07	chr12:55574269-55759877	OR10A7, OR6C74, OR6C3, OR6C6, OR6C1, OR6C75
12q13.13	2.11E-09	6.65E-07	chr12:53343710-53424773	EIF4B, KRT18
12q21.2	5.93E-20	7.50E-07	chr12:77318912-77389164	[E2F7]
12q21.31	7.35E-19	7.78E-07	chr12:82698998-82738458	[CCDC59]
12q21.2	1.12E-16	8.38E-07	chr12:78728204-78807277	[NAV3]
12q13.12	7.54E-07	9.30E-07	chr12:49608262-49667984	TUBA1C

12q14.3	6.64E-25	1.24E-06	chr12:66600289-66776259	IRAK3, GRIP1, HELB
12q21.2	7.22E-16	1.55E-06	chr12:77541499-77566243	[E2F7]
12q21.2	6.27E-22	1.59E-06	chr12:79523530-79606748	SYT1
12q21.31	2.38E-15	1.79E-06	chr12:85678997-85746111	ALX1
12q21.32	6.64E-14	2.99E-06	chr12:86789648-86861565	MGAT4C
12q14.1	1.61E-19	3.82E-06	chr12:58410811-58599712	[XRCC6BP1]
17q21.31	4.24E-06	4.24E-06	chr17:41609142-42144467	DUSP3, ETV4, MEOX1, MPP2, MPP3, PPY, PYY, FAM215A, SOST, TMEM101, LSM12, CD300LG, NAGS,
12q21.1	1.46E-17	5.35E-06	chr12:75546360-75575307	KCNC2
12q13.3	1.90E-36	6.94E-06	chr12:56924834-56989741	RBMS2, BAZ2A
12q21.1	1.49E-24	7.56E-06	chr12:71968779-72017798	LGR5, ZFC3H1
12q21.31	1.80E-21	1.87E-05	chr12:80462880-80582644	[OTOGL]
12q22	1.88E-09	2.12E-05	chr12:96068601-96096555	NTN4
12q14.1	4.36E-10	2.51E-05	chr12:59843862-59929430	[SLC16A7]
12q21.31	1.02E-18	3.25E-05	chr12:80847423-80888666	PTPRQ
12q21.1	1.77E-17	4.80E-05	chr12:75295648-75394252	[KCNC2]
12q13.11	2.23E-05	4.97E-05	chr12:46526458-46567887	[SLC38A1]
12q14.1	1.30E-08	5.52E-05	chr12:61348386-61480085	[FAM19A2]
12q23.1	2.68E-06	6.02E-05	chr12:98207978-98300149	[MIR4303]
12q21.2	2.36E-13	6.05E-05	chr12:77774632-77813991	[E2F7]
1q23.3	8.32E-06	6.24E-05	chr1:160751106-160809002	LY9, CD244
12q23.1	3.72E-08	7.21E-05	chr12:97013606-97053901	[CDK17]
12q14.1	1.92E-10	8.64E-05	chr12:60184771-60276564	[SLC16A7]
12q21.31	1.50E-09	9.43E-05	chr12:84710125-84744437	[SLC6A15]
12q12	3.84E-06	0.000154	chr12:45450858-45494764	RACGAP1P
12q22	4.95E-13	0.00017672	chr12:95444835-95546016	NR2C1, FGD6
12q21.33	1.46E-16	0.00017759	chr12:89916382-89933762	GALNT4, POC1B, POC1B-GALNT4
12q13.13	8.37E-07	0.00020654	chr12:52810539-52879947	KRT6B, KRT75, KRT6C
12q14.1	3.52E-08	0.00022705	chr12:59417689-59639968	[LRIG3]
12q23.1	4.40E-07	0.00023904	chr12:100547500-100572161	GOLGA2P5
12q21.2	3.01E-14	0.00025516	chr12:78418420-78456598	NAV3
12q21.1	8.69E-16	0.00027506	chr12:74182932-74208602	[LOC100507377]
12q21.1	3.21E-13	0.00029192	chr12:75051434-75164406	[ATXN7L3B]
12q22	1.44E-13	0.00030197	chr12:94654987-94782779	PLXNC1, CCDC41
12q14.2	9.26E-06	0.00041503	chr12:63220748-63334198	PPM1H
12q23.1	2.98E-06	0.00062566	chr12:100332104-100419161	ANKS1B
1q23.1	0.00016128	0.00082054	chr1:158000741-158278474	CD1A, CD1C, CD1D, KIRREL, LOC646268
12q23.1	0.00065456	0.00086273	chr12:98834126-98923755	TMPO, SLC9A7P1
12q13.13	6.14E-06	0.00086273	chr12:52483534-52532817	[C12orf44]
12q23.1	4.75E-05	0.00089164	chr12:99848181-99874575	ANKS1B
12q21.31	4.99E-15	0.00094693	chr12:84019009-84190812	[TMTC2]
12q15	5.78E-25	0.00099307	chr12:70607668-70645684	CNOT2
12q12	2.12E-05	0.0010225	chr12:45169682-45313419	NELL2
12q21.2	1.77E-16	0.0010312	chr12:79075438-79135050	[SYT1]
12q21.1	8.57E-21	0.001125	chr12:72643879-72779721	TRHDE
12q12	0.0012404	0.0012923	chr12:43939624-44001553	ADAMTS20
14q32.33	0.00078322	0.0013402	chr14:106215124-106294282	[ELK2AP]
6q23.3	0.00078322	0.0014818	chr6:138520936-138745177	HEBP2, KIAA1244, NHSL1, PBOV1
12q24.11	0.00019713	0.0015205	chr12:111245630-111426789	MYL2, CCDC63
12q21.33	1.08E-09	0.0015443	chr12:91504664-91565342	DCN, LUM
12q23.3	0.0012404	0.0015495	chr12:104724261-104746325	TXNRD1
12q21.32	9.02E-14	0.0019875	chr12:87280697-87342208	[MGAT4C]
12q23.2	9.52E-05	0.0020604	chr12:102605576-102619502	[PMCH]
1q24.2	0.00045132	0.0022604	chr1:168141891-169238557	hsa-mir-557, ATP1B1, DPT, XCL1, XCL2,TBX19, NME7, MGC4473, ANKRD36BP1, TIPRL, SFT2D2, MIR557
12q23.1	4.24E-06	0.0023509	chr12:97650249-97672157	[RMST]
12q14.1	1.67E-12	0.0028211	chr12:59021743-59046844	[LRIG3]
12q14.2	4.08E-05	0.0029291	chr12:63422361-63436792	[PPM1H]
12q24.11	0.00094693	0.0030408	chr12:110382490-110466069	GIT2, ANKRD13A
12q14.1	2.70E-07	0.0035065	chr12:60759438-61003195	[SLC16A7]
12q14.1	9.07E-05	0.0040852	chr12:62324725-62380941	FAM19A2
12q24.12	0.0011377	0.0042559	chr12:111892189-111919644	ATXN2
12q14.3	8.12E-15	0.0044775	chr12:65850823-65917357	MSRB3
12q23.1	8.32E-06	0.0049121	chr12:101334651-101368023	ANO4
12q23.1	2.70E-07	0.0051776	chr12:96238300-96391333	HAL, SNRPF, CCDC38, AMDHD1
12q21.2	3.21E-13	0.0053944	chr12:76392166-76419227	PHLDA1

1q21.2	0.00037251	0.0063047	chr1:148649898-148693459	[PPIAL4E]
6q24.3	0.00094693	0.006865	chr6:147713527-148943173	SASH1, SAMD5
12q23.2	8.64E-05	0.009675	chr12:101772603-101864792	ARL1, UTP20
14q32.31	0.0057773	0.011014	chr14:101652467-101955221	[DIO3OS]
15q26.2	0.012629	0.012629	chr15:96650942-102531392	hsa-mir-1302-10, hsa-mir-1469, ALDH1A3, IGF1R, MEF2A, PCSK6, SNRPA1, NR2F2, CHSY1, SYNM, OR4F4, LINS, SELS, TTC23, LRRK1, TM2D3, ARRDC4, LOC91948, TARSL2, LRRC28, ASB7, LYSMD4, PGPEP1L, SPATA8, ADAMTS17, DNM1P46, CERS3, FAM169B, WASH3P, FLJ42289, OR4F6, OR4F15, FAM138E, GPCRLTM7, DDX11L1, DDX11L9, MIR1469, MIR4714
12q24.21	0.011528	0.014651	chr12:114176778-114476804	RBM19
8q24.23	0.010125	0.015158	chr8:136923254-137150156	[KHDRBS3]
1q25.1	0.0062593	0.017244	chr1:173327552-173541183	PRDX6, SLC9A11
12q12	0.018583	0.018583	chr12:39656895-39939197	KIF21A
12q14.1	0.00030592	0.020125	chr12:62000764-62188217	FAM19A2
12q11	0.021359	0.021359	chr12:37942623-38105137	[ALG10B]
12q13.12	0.021359	0.021359	chr12:51141410-51188406	ATF1, DIP2B
11p15.5	0.021922	0.021922	chr11:1493610-2440988	hsa-mir-483, hsa-mir-675, hsa-mir-4298, ASCL2, CD81, CTSD, DUSP8, IGF2, INS, LSP1, MRPL23, TH, TNNI2, TNNT3, TSPAN32, TSSC4, C11orf21, TRPM5, IGF2-AS1, MOB2, SYT8, H19, LOC338651, KRTAP5-1, KRTAP5-3, KRTAP5-4, FAM99A, IFITM10, KRTAP5-5, KRTAP5-2, KRTAP5-6, MIR483, INS-IGF2, MIR675, FAM99B, MRPL23-AS1, MIR4298, MIR4686
12q22	6.10E-05	0.024886	chr12:93624248-93755874	LOC643339
12q24.21	0.021922	0.027663	chr12:115698857-115834550	[MED13L]
12q13.12	0.025983	0.030714	chr12:50202854-50277128	FAIM2, NCKAP5L, BCDIN3D
12q24.11	0.010594	0.031354	chr12:109683852-109877180	ACACB, FOXN4, MYO1H
1q21.2	0.0017956	0.034349	chr1:147288502-148224707	GJA8, NBPF14, GPR89B, PPIAL4A, NBPF11, FLJ39739, PPIAL4D, PPIAL4B, NBPF24, GPR89C, PDZK1P1, PPIAL4F
8q21.11	0.032181	0.037383	chr8:75644440-75764714	PI15, FLJ39080
6q21	0.023015	0.039083	chr6:112232987-112817910	LAMA4, WISP3, TUBE1, RFPL4B,
12q24.22	0.037602	0.046497	chr12:117359191-117632025	FBXO21, FBXW8, TESC
6q24.2	0.029688	0.053518	chr6:143047908-143589459	HIVEP2, AIG1
12q13.11	0.026853	0.054779	chr12:48013967-48354605	VDR, ENDOU, RAPGEF3, HDAC7, SLC48A1, RPAP3
12p12.3	0.061223	0.061223	chr12:18149710-18301820	RERGL
6q24.3	0.0085516	0.064043	chr6:146662835-146767661	GRM1
8q24.21	0.045206	0.066102	chr8:130528750-130601284	[GSDMC]
1q23.3	0.014125	0.078537	chr1:162124288-162857080	hsa-mir-556, DDR2, UAP1, NOS1AP, HSD17B7, SH2D1B, UHMK1, MIR556, MIR4654

Deletions

Cytoband	q value	Residual q value	Wide peak boundaries	Genes in wide peak
8p23.1	7.42E-13	8.31E-13	chr8:12285821-12604205	MIR3926-2, MIR3926-1
16q23.1	3.07E-09	3.07E-09	chr16:74429966-74662596	GLG1, CLEC18B
17q11.2	3.07E-09	3.07E-09	chr17:31334560-31938864	ACCN1, AA06
1q31.3	3.81E-08	3.81E-08	chr1:196709224-196859198	CFHR1, CFHR3
4q31.21	2.43E-07	2.43E-07	chr4:144806610-145030545	GYPB
18p11.21	2.43E-07	2.43E-07	chr18:14467007-18603221	hsa-mir-3156-2, ANKRD30B, POTEC, CXADRP3, MIR3156-2
7p11.2	4.51E-07	4.51E-07	chr7:56559513-57188361	hsa-mir-4283-1, DKFZp434L192, MIR4283-2, MIR4283-1
9q22.33	8.80E-07	8.80E-07	chr9:99796460-100005889	LOC340508
3q29	1.18E-06	1.18E-06	chr3:196707953-196923274	DLG1, MFI2, MFI2-AS1
3p12.3	7.30E-06	7.30E-06	chr3:74567976-75476954	MIR4444-1
4q13.3	7.25E-06	7.30E-06	chr4:75305801-75675840	AREG
10q11.23	1.72E-05	1.72E-05	chr10:51564018-51785816	NCOA4, AGAP6, TIMM23
1p36.13	1.82E-05	1.82E-05	chr1:16573098-148764767	hsa-mir-3118-3, RUNX3, CDKN2C, FABP3, ARID1A, BCAR3, SERBP1
1p22.2	4.44E-05	4.44E-05	chr1:89850349-89992530	GBP1P1
11p11.12	9.43E-05	0.00015905	chr11:48507373-49981191	FOLH1, TRIM64C
20p11.1	0.00018061	0.00018061	chr20:26028883-29695949	hsa-mir-663, MLLT10P1, NCOR1P1, FAM182A, FRG1B, MIR663A
1q43	0.00023648	0.00023648	chr1:242153019-243224174	PLD5, MAP1LC3C

19q13.33	0.00015905	0.00028244	chr19:48029946-48223862	GLTSCR1
5q35.2	0.00034081	0.00034081	chr5:175346791-175690321	THOC3, FAM153B
18q22.1	0.00038108	0.00038108	chr18:61877778-63418257	LOC400654
15q11.2	0.00064488	0.00064488	chr15:23033804-23807870	NIPA1, GOLGA8IP, WHAMMP3, GOLGA8E, HERC2P2, HERC2P7, MIR4508
9p24.3	0.0014571	0.0014571	chr9:1-844430	hsa-mir-1302-9, FOXD4, KANK1, CBWD1, DOCK8, FAM138C, WASH1
7p15.3	0.0017031	0.0017031	chr7:22393825-22603428	STEAP1B
21q11.2	0.0036862	0.0036862	chr21:1-14780335	hsa-mir-3156-3, BAGE, TPTE, BAGE5, BAGE4, BAGE3, BAGE2, ANKRD30BP2, TEKT4P2, MIR3156-3, MIR3687, MIR3648
9p13.1	0.0042201	0.0042201	chr9:38616310-68440447	ZNF658, FAM75A7, CNTNAP3, ANKRD20A1, FAM201A, FOXD4L4, AQP7P1, KGFLP1, FAM75A6, MGC21881, FAM74A1, FAM74A4, ZNF658B, ANKRD20A3, ANKRD20A2, AQP7P3, LOC442421, FAM27A, FAM75A2, FAM75A4, FAM75A1, FAM74A2 , KGFLP2, FAM75A3, FAM75A5, FAM74A3, CNTNAP3B, FOXD4L2, FAM27C, FAM95B1, FAM27B
13q14.2	0.0023299	0.0042201	chr13:47469941-50237254	RCBTB2, MLNR, RB1, SUCLA2, ITM2B, LPAR6, FNDC3A, MED4, PHF11, RCBTB1, NUDT15, CYSLTR2, CDADC1, CAB39L , SETDB2, ARL11
19q13.43	0.0029344	0.0042201	chr19:56623344-56743680	ZNF444, ZSCAN5A, GALP, ZSCAN5B
10p15.3	0.0056753	0.0056753	chr10:715352-1034808	LARP4B
5p15.33	0.0063622	0.0063622	chr5:681303-936064	TRIP13, BRD9, ZDHHC11
2q37.3	0.0078844	0.0078844	chr2:242275552-242717658	hsa-mir-3133, BOK, DTYMK, FARP2, STK25, ATG4B, THAP4, ING5, D2HGDH,
8p23.1	0.0062489	0.018584	chr8:6909101-8178099	hsa-mir-548i-3, DEFA5, DEFB4A, SPAG11B, DEFB103B, DEFB104A, DEFB105A, DEFB106A, DEFB107A, FLJ10661, LOC349196, DEFB103A, FAM90A13, FAM90A5, FAM90A7, FAM90A8, FAM90A18, FAM90A9, FAM90A10, DEFB107B, DEFB104B, DEFB106B, DEFB105B, DEFB109P1B, FAM90A14, SPAG11A, FAM90A20, FAM90A19, FAM66B, ZNF705G, FAM66E, DEFB4B, MIR548I3
13q11	0.0065013	0.018584	chr13:1-19586753	ANKRD20A9P
7q36.3	0.034436	0.034436	chr7:158347883-158539282	NCAPG2
18p11.32	0.035056	0.035056	chr18:1-3496489	ADCYAP1, CETN1, TGIF1, TYMS, YES1, MYOM1, USP14, LPIN2, THOC1, NDC80, MYL12A, SMCHD1, CLUL1, ENOSF1, LINC00470, METTL4, COLEC12, EMILIN2, MYL12B, CBX3P2, ROCK1P1
2q21.3	0.040194	0.041272	chr2:135777505-136298696	RAB3GAP1, ZRANB3
15q25.3	0.053856	0.055507	chr15:85486065-85932248	PDE8A
15q21.1	0.091597	0.094435	chr15:29855302-57535954	ACTC1, B2M, BUB1B, CAPN3, CHRM5, CHRNA7, CKMT1B, CYP19A1, DUT, EPB42, FBN1, FGF7, GABPB1, GALK2, GANC, GATM, GCHFR, PDIA3, HDC, ONECUT1, ITPKA, IVD, LTK, MAP1A, MEIS2, MFAP1, TRPM1, MYO5A, NEDD4, PLCB2, MAPK6, RAB27A, RAD51, RYR3, SCG5, SLC12A1, SORD, SPINT1, SRP14, THBS1, TJP1, TP53BP1, TYRO3, SLC30A4, EIF3J, JMJD7-PLA2G4B, SNAP23, USP8, SLC28A2 CCPG1, COPS2, TGM5, PIGB, PPIP5K1, AQR, SECISBP2L, ARHGAP11A, LCMT2, SLC12A6, BCL2L10, RASGRP1, SERF2, GNB5, ARPP19, SLC27A2, GPR176, CHP, OIP5, BAHD1, FAN1, CEP152, MAPKBP1, GOLGA8A, RTF1, MGA, DMXL2, VPS39, AP4E1, CCNDBP1, EID1, TMEM87A, RPAP1, DKFZP434L187, PYGO1, PLDN, GREM1, RPUSD2, TUBGCP4, SCG3, TMOD3, TMOD2, EHD4, DUOX2, MYEF2, NDUFAF1, RSL24D1, NUSAP1, TMEM85, SPTBN5, CTDSPL2, KLF13, DUOX1, DLL4, INO80, ZNF280D, TRPM7, PPP1R14D, MTMR10, ZNF770, FLJ10038, HAUS2, FAM82A2, DNAJC17, MNS1, NOP10, MYO5C, FAM214A, PAK6, DTWD1, CASC5, AVEN, GJD2, STARD9, VPS18, SQRDL, ZFP106, RFX7, SPATA5L1, CHAC1, ATP8B4, WDR76, TMEM62, SEMA6D, SPG11, ELL3, SPPL2A, ZFYVE19, FRMD5, DISP2, CHRFAM7A, ULK4P3, ULK4P1, ARHGAP11B, ATPBD4, BMF, SHF, DUOXA1, CHST14, CASC4, TGM7, CATSPER2, LEO1, PLA2G4E, TRIM69, FLJ27352, TMCO5A, ZSCAN29, TTBK2, CDAN1, STRC, DYX1C1, CSNK1A1P1, OTUD7A, SPRED1, PGBD4, ADAL, EXD1, FSIP1, RHOV, UBR1, PATL2, LPCAT4, PLA2G4F, LRRC57, LYSMD2, WDR72, HMGN2P46, SLC24A5, PRTG, FAM98B, PLA2G4D, GLDN, FMN1, MRPL42P5, USP50, TEX9, TNFAIP8L3, SHC4, CTXN2, DUOXA2, MIR211, GOLGA8B, EIF2AK4, CATSPER2P1, UNC13C, CKMT1A, SERINC4, PHGR1, MIR626, MIR627, MIR628, ANP32AP1, MIR147B, ANKRD63, JMJD7, PLA2G4B, ULK4P2, MIR1233-1, MIR1266, MIR1282, MIR1233-2, MIR4310, MIR3942,

				DYX1C1-CCPG1, MIR4510, MIR4716, MIR4713, MIR4712, TMCO5B
11q25	0.095602	0.099253	chr11:1-135006516	hsa-mir-3167, ACAT1, ACP2, ACRV1, ACTN3, ADM, ADRBK1, AP2A2, APLNR, ALDH3B1, ALDH3B2, AMPD3, APBB1, BIRC2, BIRC3, APLP2, APOA1, APOA4, APOC3, ARCN1, RHOG, ARHGAP1, PHOX2A, ARL2, ARNTL, ARRB1, ART1, ASCL2, ATM, FXYD2, BAD, BBS1, CCND1, BDNF, CXCR5, SERPING1, CAPN5, MRPL49, ZNHIT2, MPPED2, DAGLA, CALCA, CALCB, CAPN1, CARS, CASP1, CASP4, CASP5, CAT, CBL, SERPINH1, CCKBR, CD3D, CD3E, CD3G, CD5, CD6, MS4A1, MS4A3, CD44, CD59, CD81, CD151, CDKN1C, CFL1, CTSC, CHEK1, CHKA, TPP1, CLNS1A, CNGA4, CNTF, COPB1, COX8A, CPT1A, CRY2, CRYAB, CST6, CSTF3, CTNND1, CTSD, CTSW, DDB1, DDB2, DDX6, DDX10, DHCR7, DLAT, DLG2, DPAGT1, DRD2, DRD4, DUSP8, EEF1G, EIF4G2, ELF5, MARK2, CTTN, ESRRA, ETS1, EXT2, F2, FANCF, FAU, MS4A2, FDX1, FEN1, FGF3, FGF4, FKBP2, FLI1, FOLH1, FOLR1, FOLR2, FOLR3, FSHB, FTH1, FUT4, SLC37A4, LRRC32, GAS2, GIF, GNG3, GRIA4, GRIK4, GRM5, GSTP1, GTF2H1, GUCY1A2, H2AFX, HBB, HBBP1, HBD, HBE1, HBG1, HBG2, HMBS, SLC29A2, HPX, HRAS, HSPA8, HSPB2, DNAJC4, HTR3A, WNT11, WT1, SF1, EI24, PITPNM1, SF3B2, ROBO4 ,KDM4D, FAT3
17p13.3	0.095602	0.099253	chr17:19980-1187942	hsa-mir-3183, ABR, RPH3AL, TIMM22, GEMIN4, GLOD4, RNMTL1, VPS53, NXN, FAM57A, FAM101B, BHLHA9, DBIL5P, MIR3183
20p13	0.09994	0.10058	chr20:1-126855	DEFB126, DEFB125

Supplementary Table S3: Regions of significant amplifications and deletions identified in LPS cell lines using GISTIC analysis

Amplifications

Cytoband	q value	Residual q value	Wide peak boundaries	Genes in wide peak
10p12.31	0.00029997	0.00039097	chr10:19655595-19710331	[PLXDC2]
10p12.31	0.00040427	0.00051685	chr10:20896775-20987467	[MIR4675]
10p12.31	0.012372	0.016107	chr10:19012914-19027695	[ARL5B]
10p13	0.00064375	0.00068836	chr10:14079937-14252604	FRMD4A
12p13.33	0.055886	0.055886	chr12:1015470-1051585	RAD52, WNK1
12q14.1	1.09E-12	7.54E-12	chr12:58138328-58156509	CDK4, CYP27B1,TSPAN31
12q14.1	6.86E-06	0.00061649	chr12:59800582-59842484	[SLC16A7]
12q14.1	0.00014803	0.014673	chr12:59520401-59634484	[LRIG3]
12q14.1	0.022007	0.023472	chr12:60724462-60791213	[SLC16A7]
12q14.2	0.0095225	0.0098647	chr12:63602438-63682897	[AVPR1A]
12q14.3	1.23E-10	3.74E-08	chr12:66876205-66905404	GRIP1
12q14.3	4.28E-08	1.57E-06	chr12:65700498-65737763	MSRB3
12q14.3	5.36E-06	0.00012014	chr12:66201353-66244291	HMGA2, RPSAP52
12q14.3	5.71E-06	0.0016073	chr12:67136167-67159718	[GRIP1]
12q14.3	2.14E-05	0.0078464	chr12:67583637-67626635	[CAND1]
12q15	1.09E-12	5.58E-08	chr12:69120642-69497994	CPM, MDM2, SLC35E3, NUP107
12q15	1.09E-12	3.26E-07	chr12:70588640-70948244	CNOT2, PTPRB, KCNMB4
12q15	1.19E-06	5.35E-05	chr12:67976530-68034946	[DYRK2]
12q15	1.09E-12	6.31E-05	chr12:69833857-70341113	CCT2, FRS2, RAB3IP, BEST3, LRRC10, MIR3913-2, MIR3913-1
12q15	2.09E-06	0.00023026	chr12:71424403-71499138	[TSPAN8]
12q15	4.33E-05	0.00085156	chr12:68698087-68743910	MDM1
12q15	0.00029997	0.0032901	chr12:68399872-68418829	[IFNG]
12q21.2	0.00074323	0.00074323	chr12:79736856-79779416	SYT1
12q21.2	0.00064375	0.00090708	chr12:77440582-77537764	E2F7
12q21.2	0.00025681	0.0023199	chr12:76088474-76190013	[KRR1]
12q21.2	0.0003293	0.0031978	chr12:76599133-76769208	BBS10, OSBPL8
12q21.2	0.010334	0.01255	chr12:77873500-77886821	[NAV3]
12q21.31	0.0027937	0.0027937	chr12:83169264-83293247	TMTC2
12q21.32	0.0011898	0.0033447	chr12:87150442-87214973	MGAT4C
12q21.32	0.013448	0.039649	chr12:87386836-87455866	[MGAT4C]
12q21.32	0.052864	0.052864	chr12:87964219-88033785	[MKRN9P]
12q22	0.035362	0.035362	chr12:92840475-93121119	C12orf74
12q22	0.064697	0.084633	chr12:95307796-95324036	[NDUFA12]
12q23.1	0.0095225	0.011727	chr12:99770587-99870491	ANKS1B

12q23.1	0.011128	0.014593	chr12:99660833-99728573	ANKS1B
12q23.1	0.032263	0.041997	chr12:99542009-99660098	ANKS1B
12q24.12	0.010566	0.010566	chr12:111793040-112146554	ATXN2, BRAP, SH2B3, ACAD10, FAM109A
14q12	0.00085156	0.00085156	chr14:31993088-32312099	NUBPL
14q12	0.0027937	0.0027937	chr14:30500354-30701040	[PRKD1]
14q13.1	6.81E-06	6.81E-06	chr14:33548253-33594963	NPAS3
14q13.2	0.080244	0.080244	chr14:35998387-36066502	INSM2, RALGAPA1
14q13.3	0.037354	0.037354	chr14:37045099-37078408	NKX2-8
1p32.1	3.43E-06	3.43E-06	chr1:58820186-60059134	JUN, TACSTD2, FGGY, MYSM1, OMA1, HSD52,
1q21.3	0.020233	0.020233	chr1:151666369-151893162	RORC, TDRKH, CELF3, OAZ3, MRPL9, SNX27, THEM4
1q23.2	0.00066944	0.00066944	chr1:159922630-160112740	ATP1A2, KCNJ9, KCNJ10, SLAMF9, PIGM, IGSF8
1q23.3	0.00039165	0.00039165	chr1:163511426-163581060	[NUF2]
1q23.3	0.097285	0.097285	chr1:164876566-165562499	LMX1A, RXRG, LRRC52
1q24.1	0.0038701	0.0038701	chr1:166155636-166594959	FMO9P
1q24.2	6.86E-06	0.00059582	chr1:169410487-169536239	F5, SLC19A2
1q24.2	7.25E-05	0.0041101	chr1:168235649-168389916	hsa-mir-557, TBX19, MIR557
1q24.3	7.55E-06	2.75E-05	chr1:172071716-172342198	hsa-mir-199a-2, DNM3, MIR199A2, MIR214, MIR3120, DNM3OS
1q25.1	0.014763	0.014763	chr1:174414490-174592589	GPR52, RABGAP1L
1q31.1	6.51E-05	8.69E-05	chr1:189552872-189581461	[FAM5C]
1q31.1	0.00078526	0.00094477	chr1:189035126-189098832	[FAM5C]
1q31.1	0.061413	0.084633	chr1:185741998-186722112	PDC, PTGS2, TPR, PRG4, OCLM, HMCN1
1q31.3	0.010334	0.010334	chr1:197081823-197302689	CRB1, ASPM, ZBTB41
1q32.1	0.033975	0.037354	chr1:204968544-204993934	NFASC
1q42.12	0.018093	0.018876	chr1:226929445-227019072	[ITPKB]
1q43	0.0095225	0.010115	chr1:241173768-241236902	RGS7
20q11.23	0.017277	0.01974	chr20:35826684-35909293	GHRH, RPN2
20q12	0.008263	0.0095225	chr20:39491982-40266417	PLCG1, TOP1, ZHX3, LPIN3, CHD6, EMILIN3
20q12	0.044717	0.055886	chr20:40587652-40719391	PTPRT
20q12	0.052864	0.06576	chr20:38717801-38799749	[MAFB]
2p16.1	0.044717	0.044717	chr2:61262135-61318279	PEX13, KIAA1841
2q33.2	0.035362	0.042767	chr2:202882898-205441669	BMPR2. CD28, CTLA4, SUMO1, FZD7, ABI2, ICOS
2q36.1	0.0093573	0.0099329	chr2:222686400-223195453	PAX3, CCDC140
5p15.33	0.080244	0.080244	chr5:1236691-1308551	TERT, SLC6A18
5q35.2	0.099933	0.099933	chr5:105573854-180915260	hsa-mir-340, hsa-mir-1229, hsa-mir-4281, hsa-mir-1271, hsa-mir-585, hsa-mir-218-2, hsa-mir-103-1-as
7p15.3	0.044717	0.044717	chr7:202180-38711293	hsa-mir-1200, hsa-mir-548n, hsa-mir-550-2, hsa-mir-550-1, hsa-mir-196b, hsa-mir-148a, hsa-mir-1183
8q11.21	0.017277	0.017277	chr8:51015523-51230278	SNTG1
8q13.1	0.052864	0.07074	chr8:67451231-68167535	MYBL1, ARFGEF1, COPS5, SGK3, PTTG3P, CSPP1
8q21.12	0.032263	0.099933	chr8:79737017-79998536	[IL7]

8q21.3	0.0095225	0.0198	chr8:88440494-88528651	[CNBD1]
8q21.3	0.01123	0.025604	chr8:93245260-93391781	[RUNX1T1]
8q21.3	0.015285	0.034967	chr8:90164240-90308656	[RIPK2]
8q24.13	0.021134	0.037354	chr8:126914647-126970139	LOC100130231
9q31.2	0.048319	0.048319	chr9:110506875-110910873	[KLF4]

Deletions

Cytoband	q value	Residual q value	Wide peak boundaries	Genes in wide peak
8p23.3	0.008658	0.008658	chr8:1-2793552	hsa-mir-596, CLN8, MYOM2, DLGAP2, ARHGEF10, KBTBD11, FBXO25, ERICH1, ZNF596, RPL23AP53, MIR596
2q37.2	0.034867	0.034867	chr2:235962488-237091316	GBX2, AGAP1
3p14.2	0.088806	0.088806	chr3:59034499-61552091	FHIT
16q12.2	0.09201	0.088806	chr16:32954256-54321374	hsa-mir-3181, hsa-mir-1826, ADCY7, CBLN1, CYLD, PHKB, RBL2, SALL1, SIAH1, N4BP1, DNAJA2, ZNF423, RPGRIP1L, ORC6, TP53TG3, TOX3, BRD7, HEATR3, VPS35, NOD2, PAPD5, AKTIP, FTO, IRX3, SHCBP1, CHD9, ITFG1, NETO2, LONP2, GPT2, ABCC11, NKD1, MYLK3, ABCC12, ANKRD26P1, SNX20, CNEP1R1, FLJ26245, UBE2MP1, LINC00273, TP53TG3C, TP53TG3B, MIR548AE2

Supplementary Table S4. Coverage analysis of whole exome sequencing of 12 LPSs and matched adjacent normal tissues in Discovery Cohort

Sample ID	Sample type	Bait set	Captured region	Genome size	Bait size	Mean target coverage	Target coverage >20x
LPS11	Germline	SureSelect50M	Whole Exome	3095693983	51756122	208.6891781	73.088
LPS11	Tumor	SureSelect50M	Whole Exome	3095693983	51756122	298.7831602	77.97
LPS12	Germline	SureSelect50M	Whole Exome	3095693983	51756122	132.295527	69.97
LPS12	Tumor	SureSelect50M	Whole Exome	3095693983	51756122	293.2495018	83.21
LPS13	Germline	SureSelect50M	Whole Exome	3095693983	51756122	176.8267962	75.12
LPS13	Tumor	SureSelect50M	Whole Exome	3095693983	51756122	165.5077471	74.23
LPS19	Germline	SureSelect50M	Whole Exome	3095693983	51756122	135.7619357	70.38
LPS19	Tumor	SureSelect50M	Whole Exome	3095693983	51756122	183.9084983	72.92
LPS22	Germline	SureSelect50M	Whole Exome	3095693983	51756122	185.807918	73.33
LPS22	Tumor	SureSelect50M	Whole Exome	3095693983	51756122	144.8161724	71.85
LPS55	Germline	SureSelect50M	Whole Exome	3095693983	51756122	128.8789802	66.26
LPS55	Tumor	SureSelect50M	Whole Exome	3095693983	51756122	101.7150395	64.26
LPS80	Germline	SureSelect50M	Whole Exome	3095693983	51756122	205.7742496	84.97
LPS80	Tumor	SureSelect50M	Whole Exome	3095693983	51756122	170.7522847	80.06
LPS86	Germline	SureSelect50M	Whole Exome	3095693983	51756122	198.348464	89.19
LPS86	Tumor	SureSelect50M	Whole Exome	3095693983	51756122	256.59176	96.51
LPS87	Germline	SureSelect50M	Whole Exome	3095693983	51756122	181.441421	80.51
LPS87	Tumor	SureSelect50M	Whole Exome	3095693983	51756122	213.924536	94.61
LPS88	Germline	SureSelect50M	Whole Exome	3095693983	51756122	174.0940952	86.76
LPS88	Tumor	SureSelect50M	Whole Exome	3095693983	51756122	237.6752802	84.71
LPS89	Germline	SureSelect50M	Whole Exome	3095693983	51756122	150.6893255	73.43
LPS89	Tumor	SureSelect50M	Whole Exome	3095693983	51756122	191.6607602	94.6
LPS90	Germline	SureSelect50M	Whole Exome	3095693983	51756122	154.8696351	77.87
LPS90	Tumor	SureSelect50M	Whole Exome	3095693983	51756122	156.0966313	91.19

Supplementary Table S5. Summary of somatic mutations in the Discovery Cohort

Sample ID	Gene Symbol	Chromosome	Genomic position	Nucleotide change (reference|variant)	cDNA position	Amino acid position	Amino acid change	Mutation type
LPS11	*INTS2*	chr17	59955334	C|G	2394	798	S>R	missense
LPS11	*MDC1*	chr6	30673497	G|C	3463	1155	V>L	missense
LPS11	*DNAJC14*	chr12	56221343	C|G	1100	367	S>C	missense
LPS11	*VEPH1*	chr3	157178123	delA	376	126	M>fs	frameshift deletion
LPS11	*OR6B1*	chr7	143701831	A|G	742	248	I>V	missense
LPS11	*MUC12*	chr7	100647851	C|G	14007	4669	H>Q	missense
LPS11	*WDR96*	chr10	105923977	G|A	3121	1041	V>M	missense
LPS11	*ZBTB38*	chr3	141164779	G|T	3549	1183	K>N	missense
LPS11	*MYH3*	chr17	10552985	G|A	551	184	G>E	missense
LPS11	*PLBD1*	chr12	14706266	G|A	196	66	A>T	missense
LPS11	*PADI2*	chr1	17409049	G|T	1149	383	K>N	missense
LPS11	*PRKD3*	chr2	37543624	T|G	44	15	L>X	stopgain
LPS11	*SPHKAP*	chr2	228890137	T|A	414	138	N>K	missense
LPS11	*RPUSD2*	chr15	40863818	C|T	622	208	R>W	missense
LPS11	*STXBP3*	chr1	109350028	G|A	1541	514	R>H	missense
LPS11	*GPR109A*	chr12	123187530	C|T	301	101	R>W	missense
LPS11	*MYST2*	chr17	47893243	C|G	841	281	R>G	missense
LPS11	*FOLR2*	chr11	71932639	delG	601	201	G>fs	frameshift deletion
LPS11	*INTS6*	chr13	51957533	G|T	1112	371	S>I	missense
LPS11	*PCDH15*	chr10	56128994	C|A	360	120	C>X	stopgain
LPS11	*EBF3*	chr10	131676052	616	206	206	R>G	missense
LPS11	*MXRA5*	chrX	3242785	C|T	941	314	P>L	missense
LPS11	*DNAH17*	chr17	76497874	A|C	5272	1758	T>P	missense
LPS11	*C8orf80*	chr8	27880848	insC	2377-2378	793	G>fs	frameshift deletion
LPS11	*OTOP3*	chr17	72937649	C|T	235	79	R>W	missense
LPS11	*PVRL4*	chr1	161043089	C|A	1234	412	P>T	missense
LPS11	*DEFB126*	chr20	126310	del	313-314			frameshift deletion
LPS12	*C6orf138*	chr6	47846301	T|A	2279	760	L>X	stopgain
LPS12	*SEC63*	chr6	108214765	T|A	1595	532	L>X	stopgain
LPS12	*C6orf138*	chr6	47846300	G|T	2280	760	L>F	missense
LPS12	*THSD7A*	chr7	11581104	C|A	1764	588	N>K	missense
LPS12	*DARS2*	chr1	173826737	A|G	1832	611	H>R	missense
LPS12	*MTDH*	chr8	98712005	C|T	1072	358	P>Q	stopgain
LPS12	*USP34*	chr2	61472389	G|T	6583	2195	D>Y	missense
LPS12	*UBQLNL*	chr11	5537428	C|T	244	82	H>Y	missense
LPS12	*EI24*	chr11	125448147	G|T	435	145	W>C	missense
LPS12	*CHD6*	chr20	40102131	G|A	2495	832	R>Q	missense
LPS12	*PUM1*	chr1	31418331					splice site
LPS12	*ABCC3*	chr17	48746831	C|A	2183	728	A>D	missense
LPS12	*FER1L6*	chr8	124978508	insA	362-363	121	T.fs	frameshift insertion
LPS12	*CDH11*	chr16	65025731	A|G	751	251	T>A	missense
LPS12	*HTR1F*	chr3	88040257	G|A	358	120	D>N	missense
LPS13	*ZNF611*	chr19	53209796	A|C	512	171	Q>P	missense
LPS13	*SLITRK2*	chrX	144904038	G|C	95	32	R>P	missense
LPS13	*MAGEB6*	chrX	26212444	G|A	481	161	V>I	missense
LPS13	*LY75*	chr2	160697407	C|G	3340	1114	L>V	missense
LPS13	*KIAA1370*	chr15	52906055					splice site

LPS13	*WDSUB1*	chr2	160112728	C|G	1111	371	L>V	missense
LPS13	*GOLIM4*	chr3	167750520	C|T	964	322	Q>X	stopgain
LPS13	*CAMKK2*	chr12	121706440					splice site
LPS13	*FAN1*	chr15	31196935	delG	69	23	K>fs	frameshift deletion
LPS13	*GOLIM4*	chr3	167750423	C|T	1061	354	A>V	missense
LPS13	*ADH6*	chr4	100137360	G|T	78	26	E>D	missense
LPS13	*SEMA3A*	chr7	83631303	G|A	1420	474	E>K	missense
LPS13	*ARAF*	chrX	47424531	C|T	451	151	R>C	missense
LPS13	*RNF32*	chr7	156437272	G|A	95	32	R>Q	missense
LPS13	*NBPF14*	chr1	148016431	G|C	811	271	D>H	missense
LPS13	*PDE1C*	chr7	31912990	C|A	524	175	S>Y	missense
LPS13	*PRPS1L1*	chr7	18066969	A|C	437	146	Y>S	missense
LPS13	*PRPS1L1*	chr7	18066972	T|G	434	145	L>W	missense
LPS13	*GOLIM4*	chr3	167747683	C|T	1318	440	Q>X	stopgain
LPS13	*CADM1*	chr11	115085349	G|C	973	325	D>H	missense
LPS13	*TYW1*	chr7	66489956	A|C	931	311	I>L	missense
LPS13	*CADM1*	chr11	115085466	G|A	856	286	E>K	missense
LPS13	*TYR*	chr11	88924547	G|A	997	333	D>N	missense
LPS13	*PRPS1L1*	chr7	18066970	T|A	436	146	Y>N	missense
LPS13	*CCDC88C*	chr14	91780245	C|T	1915	639	R>X	stopgain
LPS13	*MGAT3*	chr22	39884328	G|A	976	326	G>S	missense
LPS13	*ROBO1*	chr3	79174663	G|A	115	39	D>N	missense
LPS13	*PRUNE2*	chr9	79251444	T|C	8993	2998	I>T	missense
LPS13	*SPATA3*	chr2	231861092	G|T	144	48	Q>H	missense
LPS13	*TP53TG5*	chr20	44004156	T|A	291	97	N>K	missense
LPS13	*SLC38A10*	chr17	79225074	insA	2284-2285	762	H>fs	frameshift insertion
LPS13	*CGNL1*	chr15	57731031	C|A	834	278	F>L	missense
LPS13	*PCMTD1*	chr8	52732981	G|C	1004	335	R>T	missense
LPS19	*OR8H2*	chr11	55872750	G|A	232	78	V>I	missense
LPS19	*TTI1*	chr20	36640690	A|G	1529	510	E>G	missense
LPS19	*KIAA2026*	chr9	5923195	C|T	2801	934	S>F	missense
LPS19	*ZMYM4*	chr1	35846897	delT	1219	407	F>fs	frameshift deletion
LPS19	*DRGX*	chr10	50599241	G|A	116	39	R>H	missense
LPS19	*OR2A14*	chr7	143826338	T|G	133	45	F>V	missense
LPS19	*SEZ6L*	chr22	26709785	G|C	1932	644	W>C	missense
LPS19	*COG5*	chr7	107188579	A|G	487	163	T>A	missense
LPS19	*CCDC93*	chr2	118743603	G|A	562	188	A>T	missense
LPS19	*RNF182*	chr6	13977564	T|A	214	72	C>S	missense
LPS19	*DPP6*	chr7	154645537	A|C	1714	572	K>Q	splice site
LPS19	*USP48*	chr1	22055167	A|T	1346	449	E>V	missense
LPS19	*HEPHL1*	chr11	93778983	G|T	315	105	R>S	missense
LPS19	*CACNA1D*	chr3	53835356	G|T	5312	1771	R>L	missense
LPS19	*DOCK4*	chr7	111407174	delT	3802	1268	C>fs	frameshift deletion
LPS19	*ZNF75D*	chrX	134421124	T|G	1478	493	L>R	missense
LPS19	*DST*	chr6	56504124	T|C	1286	429	I>T	missense
LPS19	*MYO3A*	chr10	26385530	A|T	1695	565	Q>H	missense
LPS19	*RYR2*	chr1	237811806	G|T	7405	2469	V>F	missense
LPS19	*PHKA1*	chrX	71838560	A|C	2369	790	K>T	splice site
LPS19	*ADAMTSL3*	chr15	84659942	G|A	3949	1317	V>M	missense
LPS19	*CYP2C19*	chr10	96541590	T|C	655	219	F>L	missense
LPS19	*ZPBP*	chr7	50057893	A|C	723	241	E>D	missense
LPS19	*FLG*	chr1	152276236	C|T	11126	3709	S>F	missense
LPS19	*NPFFR2*	chr4	72994349	A|T	41	14	H>L	missense

LPS19	*C4orf41*	chr4	184614297	C|T	2234	745	T>I	missense
LPS19	*WFS1*	chr4	6302889	G|T	1367	456	R>L	missense
LPS19	*OR51B4*	chr11	5323119	G|A	58	20	A>T	missense
LPS19	*NEUROD4*	chr12	55420831	A|C	608	203	N>T	missense
LPS19	*ROS1*	chr6	117683862	C|G	3285	1095	N>K	missense
LPS19	*TNC*	chr9	117810785	A|C	4606	1536	T>P	missense
LPS19	*PTPRK*	chr6	128298118	G|T	3793	1265	D>Y	missense
LPS19	*TECTB*	chr10	114053589	T|G	577	193	L>V	missense
LPS19	*F8*	chrX	154133245	T|G	5427	1809	I>M	missense
LPS19	*CSMD2*	chr1	34166152	A|G	3578	1193	E>G	missense
LPS19	*STXBP5L*	chr3	121126206	G|A	2776	926	E>K	missense
LPS19	*APBA2*	chr15	29393960	G|T	1497	499	M>I	missense
LPS19	*NCKAP1L*	chr12	54905623	C|T	622	208	R>C	missense
LPS19	*OR4C3*	chr11	48347099	G|T	607	203	A>S	missense
LPS19	*GPSM2*	chr1	109465168	T|A	1570	524	S>T	missense
LPS19	*OR6N1*	chr1	158736021	G|T	452	151	G>V	missense
LPS19	*CPA3*	chr3	148601596	G|T	975	325	E>D	missense
LPS19	*OR51I1*	chr11	5462147	T|A	598	200	Y>N	missense
LPS19	*ZNF169*	chr9	97062120	A|C	280	944	S>R	missense
LPS19	*DLG4*	chr17	7099860	A|T	1247	416	E>V	missense
LPS19	*FRG1*	chr4	190876305	A|T	4311	144	N>I	splice site
LPS19	*DLG2*	chr11	83170874	A|T	2600	867	K>M	missense
LPS19	*MYF5*	chr12	81112787	C|T	725	242	T>I	missense
LPS19	*HSD17B2*	chr16	82104644	C|A	576	192	N>K	missense
LPS19	*ADAMTS4*	chr1	161167964	G|A	454	152	V>M	missense
LPS19	*SCN3A*	chr2	166032778	A|G	127	43	N>D	missense
LPS19	*TSR2*	chrX	54469838	T|G	178	60	L>V	missense
LPS19	*MIPOL1*	chr14	37754605	A|C	576	192	R>S	missense
LPS19	*CADM1*	chr11	115111057	G|A	208	70	D>N	missense
LPS19	*TBX19*	chr1	168250438	C|T	110	37	T>M	missense
LPS19	*DBX2*	chr12	45410076	C|T	1013	338	A>V	missense
LPS19	*DUSP27*	chr1	167096065	G|A	1697	566	S>N	missense
LPS19	*MCF2*	chrX	138689933	A|C	1587	529	E>D	missense
LPS19	*TBC1D8B*	chrX	106083284	G|A	1360	454	E>K	missense
LPS19	*TRPC3*	chr4	122833094	T|C	1496	499	I>T	missense
LPS19	*SOX5*	chr12	23687304	G|A	2102	701	G>D	missense
LPS19	*NOX3*	chr6	155732371	C|T	1432	478	L>F	missense
LPS19	*WDR16*	chr17	9532040	G|A	873	291	W>X	stopgain
LPS19	*POLE*	chr12	133210961	G|T	5815	1939	D>Y	missense
LPS19	*NEFL*	chr8	24811149	A|C	1330	444	T>P	missense
LPS19	*HEATR4*	chr14	73989616	C|T	100	34	R>X	stopgain
LPS19	*BTBD7*	chr14	93714934	T|C	2009	670	V>A	missense
LPS19	*POLN*	chr4	2172860	C|T	1423	475	R>W	missense
LPS19	*LCP1*	chr13	46721106	T|A	1111	371	F>I	missense
LPS19	*DCHS2*	chr4	155249343	T|G	3920	1307	V>G	splice site
LPS19	*ADAMDEC1*	chr8	24251599	A|C	65	22	D>A	missense
LPS22	*ZNF135*	chr19	58578553	G|A	737	246	R>Q	missense
LPS22	*STOX1*	chr10	70645656	G|T	2102	702	D>Y	missense
LPS22	*FAT3*	chr11	92532456	G|A	6277	2093	V>I	missense
LPS22	*DEFB136*	chr8	11831558	T|A	125	42	F>Y	missense
LPS22	*ST8SIA3*	chr18	55024338	G|A	497	166	G>E	missense
LPS22	*DOPEY1*	chr6	83847067	G|T	3279	1093	Q>H	missense
LPS22	*HEG1*	chr3	124728625	G|A	3117	1039	M>I	missense

LPS22	*OR5M3*	chr11	56237775	G|T	199	67	V>F	missense
LPS22	*PIK3CA*	chr3	178952072	A|G	3127	1043	M>V	missense
LPS22	*MIA2*	chr14	39716604	C|T	826	276	H>Y	missense
LPS22	*CSMD3*	chr8	113299314	G|A	8803	2935	G>R	missense
LPS22	*SPOCK3*	chr4	167921546	G|A	304	102	D>N	missense
LPS22	*KIAA1033*	chr12	105508205	G|C	321	107	E>D	splice site
LPS22	*ITSN2*	chr2	24426580	G|C	5009	1670	R>P	missense
LPS22	*ANKS3*	chr16	4777074	G|A	275	92	G>E	missense
LPS22	*STAC2*	chr17	37369376	G|C	1003	335	G>R	missense
LPS22	*MCM6*	chr2	136616885	A|C	1348	450	M>L	missense
LPS22	*SLC5A3*	chr21	35468125	T|G	628	210	F>V	missense
LPS55	*PRIM2*	chr6	57183385	G|C	142	48	D>H	missense
LPS55	*PRIM2*	chr6	57183289	G|A	46	16	D>N	missense
LPS55	*XIRP2*	chr2	168106624	C|A	8722	2908	Q>K	missense
LPS55	*FANCB*	chrX	14871193	G|A	1294	432	G>R	missense
LPS55	*PLCB4*	chr20	9434025	C|T	2876	959	T>M	missense
LPS55	*BRCA1*	chr17	41244253	C|T	32295	1099	P>S	missense
LPS55	*DEFB125*	chr20	76699	C|T	112	38	R>X	stopgain
LPS55	*TRIM32*	chr9	119461673	C|T	1652	551	S>F	missense
LPS55	*SFTPA2*	chr10	81318642	G|T	292	98	G>W	splice site
LPS55	*ZDHHC4*	chr7	6628405	G|A	899	300	R>H	missense
LPS55	*SCN9A*	chr2	167151134	delC	940	314	L>fs	frameshift deletion
LPS55	*TEP1*	chr14	20869301					splice site
LPS55	*NHSL1*	chr6	138754647	A|T	847	283	I>F	missense
LPS55	*PDE3A*	chr12	20807134	C|T	3179	1060	S>F	missense
LPS55	*XPO1*	chr2	61729141	T|A	398	133	I>N	missense
LPS55	*ZC3HC1*	chr7	129680877	T|G	323	108	V>G	missense
LPS80	*FMR1NB*	chrX	147084825	T|G	382	128	F>V	missense
LPS80	*TSN*	chr2	122522834	A|G	578	193	K>R	missense
LPS80	*DOCK7*	chr1	63099204	C|T	1081	361	P>S	missense
LPS80	*TPP2*	chr13	103301494	G|C	2866	956	D>H	missense
LPS80	*KCNK10*	chr14	88658663	T|C	758	253	V>A	missense
LPS80	*MCTS1*	chrX	119740017	G|C	251	84	R>T	missense
LPS80	*CYTIP*	chr2	158300390	C|T	143	48	T>M	missense
LPS80	*KRTAP10-11*	chr21	46066767	G|T	392	132	S>I	missense
LPS80	*ASB9*	chrX	15268665	C|T	455	152	S>F	missense
LPS80	*FAM47C*	chrX	37027222	C|T	739	247	R>C	missense
LPS80	*MGAM*	chr7	141727465	G|A	1151	384	R>H	missense
LPS80	*ZNF280C*	chrX	129370191	G|A	768	256	M>I	missense
LPS80	*HDGFRP3*	chr15	83820013	A|G	560	187	N>S	missense
LPS80	*WDR66*	chr12	122439498	G|A	3331	1111	V>I	missense
LPS80	*PLCB3*	chr11	64022867	G|T	468			splice site
LPS80	*CDH12*	chr5	21752012	C|T	2219	740	A>V	missense
LPS80	*NDUFA9*	chr12	4778952	G|A	769	257	D>N	missense
LPS80	*COG1*	chr17	71193530	C|G	908	303	P>R	missense
LPS80	*B2M*	chr15	45003745	A|G	1	1	M>V	missense
LPS80	*DNAAF1*	chr16	84182654	C|T	1677	56	T>I	missense
LPS80	*DNAH14*	chr1	225452889	C|T	7210	2404	P>S	missense
LPS80	*PLEC*	chr8	144995168	C|G	8779	2927	L>V	missense
LPS80	*SEMA3G*	chr3	52474021	C|T	1237	413	L>F	missense
LPS80	*IQGAP1*	chr15	91026781	A|C	3744	1248	L>F	missense
LPS80	*RYR2*	chr1	237617829	delC	1431	477	N>fs	frameshift deletion
LPS80	*STARD3*	chr17	37815053	C|T	572	191	S>L	missense

LPS80	*SVIL*	chr10	29762864	G|A	4154	1385	C>Y	missense
LPS80	*DNAJB3*	chr2	234652378	G|A	185	62	R>H	missense
LPS80	*PPFIA2*	chr12	81693145	G|A	1360	454	E>K	missense
LPS80	*SYT6*	chr1	114680443	C|G	745	249	R>G	missense
LPS80	*MUC4*	chr3	195509353	C|T	9098	3033	A>V	missense
LPS80	*COL2A1*	chr12	48372514	G|A	2554	852	G>S	missense
LPS80	*MUC4*	chr3	195518119	T|A	332	111	M>K	missense
LPS80	*FRG1*	chr4	190876263	A|T	389	130	D>V	missense
LPS80	*MUC4*	chr3	195509354	G|A	9097	3033	A>T	missense
LPS86	*XDH*	chr2	31587076	A|G	2579	860	L>P	missense
LPS86	*RBM43*	chr2	152107541	G|A	953	318	S>L	missense
LPS86	*PLXND1*	chr3	129324372	C|T	1111	371	V>I	missense
LPS86	*QRFPR*	chr4	122250816	C|T	949	317	V>M	missense
LPS86	*DMGDH*	chr5	78329170	C|T	1255	419	E>K	missense
LPS86	*SLC17A4*	chr6	25777129	G|T	1210	404	A>S	missense
LPS86	*CHD7*	chr8	61654751	C|T	760	254	Q>*	stopgain
LPS86	*ZNF34*	chr8	145999151	C|G	1120	374	E>Q	missense
LPS86	*ZNF34*	chr8	145999235	C|G	1036	346	D>H	missense
LPS86	*ZNF34*	chr8	145999270	C|G	1001	334	R>T	missense
LPS86	*ZIC5*	chr13	100617887	G|A	1736	579	P>L	missense
LPS86	*MYH7*	chr14	23884969	G|A	5026	1676	R>W	missense
LPS86	*AKAP6*	chr14	33292249	T|G	5230	1744	S>A	missense
LPS86	*MAP1A*	chr15	43814392	G|A	1435	479	V>M	missense
LPS86	*HERC1*	chr15	64067071	A|G	752	251	L>P	missense
LPS86	*ZNF597*	chr16	3486428	G|A	1271	424	T>M	missense
LPS86	*DNAH9*	chr17	11593254	C|T	4115	1372	A>V	missense
LPS86	*ATPAF2*	chr17	17921916	T|C	817	273	I>V	missense
LPS86	*WTIP*	chr19	34991160	G|A	1279	427	V>I	missense
LPS86	*DSCR3*	chr21	38600081	C|T	685	229	A>T	missense
LPS86	*DMD*	chrX	31187650	G|T	10463	3488	P>H	missense
LPS86	*ARMCX4*	chrX	100748329	T|A	4753	1585	F>I	missense
LPS86	*LRCH2*	chrX	114391189	T|G	1505	502	K>T	missense
LPS86	*PLXNB3*	chrX	153032985	G|A	703	235	V>I	missense
LPS86	*NF1*	chr17	29664385	G|A	6428-1			splice site
LPS87	*ZNF362*	chr1	33745956	G|A	581	194	R>H	missense
LPS87	*SLAMF1*	chr1	160604670	C|A	433	145	E>*	stopgain
LPS87	*FAM129A*	chr1	184772762	G|T	1511	504	T>K	missense
LPS87	*LYPD6B*	chr2	150069590	A|C	413	138	E>A	missense
LPS87	*USP40*	chr2	234418712	T|C	2593	865	T>A	missense
LPS87	*WDR19*	chr4	39230232	A|G	1904	635	H>R	missense
LPS87	*CPEB4*	chr5	173317104	C|A	368	123	S>Y	missense
LPS87	*B3GAT2*	chr6	71603912	G|A	655	219	R>C	missense
LPS87	*RP1*	chr8	55539267	C|T	2825	942	T>M	missense
LPS87	*RBM4*	chr11	66433414	C|T	500	167	S>F	missense
LPS87	*ODZ4*	chr11	78565191	C|T	1639	547	G>R	missense
LPS87	*KCNA6*	chr12	4919736	G|A	529	177	A>T	missense
LPS87	*LUM*	chr12	91502656	G|A	101	34	S>L	missense
LPS87	*RNF113B*	chr13	98828894	G|A	597	199	Y>X	stopgain
LPS87	*VWA3A*	chr16	22137515	G|A	1549	517	D>N	missense
LPS87	*DNAH9*	chr17	11666901	G|C	7140	2380	W>C	missense
LPS87	*LYZL6*	chr17	34266356	G|A	5	2	T>I	missense
LPS87	*SLC14A2*	chr18	43258987	G|T	2560	854	V>L	missense
LPS87	*TIMM44*	chr19	8002971	G|A	253	85	R>C	missense

LPS87	*ZNF836*	chr19	52660018	T|A	918	306	E>D	missense
LPS87	*NECAB3*	chr20	32248155	A|C	434	145	L>R	missense
LPS87	*IFNAR2*	chr21	34635371	G|A	1114	372	E>K	missense
LPS87	*CLIC6*	chr21	36042475	G|A	788	263	G>E	missense
LPS87	*CCR6*	chr6	167549632	G|A	9+1			splice site
LPS88	*RNF220*	chr1	45088446	C|T	791	264	P>L	missense
LPS88	*EPS8L3*	chr1	110293960	C|G	1580	527	R>P	missense
LPS88	*TRIM46*	chr1	155152336	G|A	1445	482	R>H	missense
LPS88	*SFTPB*	chr2	85892818	G|A	529	177	R>W	missense
LPS88	*SEL1L3*	chr4	25792119	C|A	2034	678	M>I	missense
LPS88	*CCDC125*	chr5	68606983	T|A	412	138	R>X	stopgain
LPS88	*N4BP3*	chr5	177548902	T|G	1535	512	M>R	missense
LPS88	*ZFHX4*	chr8	77775818	G|A	9868	3290	V>I	missense
LPS88	*ZFAT*	chr8	135521897	C|T	3421	1141	A>T	missense
LPS88	*CRTAM*	chr11	122726507	C|T	595	199	R>X	stopgain
LPS88	*INHBE*	chr12	57849952	G|A	374	125	R>H	missense
LPS88	*CUX2*	chr12	111748247	A|G	1661	554	D>G	missense
LPS88	*FBRSL1*	chr12	133151016	A|G	1696	566	S>G	missense
LPS88	*RPS6KL1*	chr14	75376641	T|G	875	292	E>A	missense
LPS88	*DISP2*	chr15	40660483	A|T	2170	724	S>C	missense
LPS88	*DISP2*	chr15	40660486	C|G	2173	725	P>A	missense
LPS88	*DISP2*	chr15	40660873	G|C	2560	854	G>R	missense
LPS88	*DISP2*	chr15	40660883	T|A	2570	857	L>H	missense
LPS88	*DISP2*	chr15	40660891	G|C	2578	860	G>R	missense
LPS88	*DISP2*	chr15	40660897	T|C	2584	862	S>P	missense
LPS88	*UBE2I*	chr16	1370177	A|G	226	76	K>E	missense
LPS88	*RGL3*	chr19	11527669	G|A	212	71	A>V	missense
LPS88	*WDR62*	chr19	36592211	C|G	2963	988	P>R	missense
LPS88	*MAGEE1*	chrX	75649198	C|T	875	292	S>L	missense
LPS88	*MAGEE1*	chrX	75649512	C|T	1189	397	P>S	missense
LPS88	*MAGEE1*	chrX	75649522	C|A	1199	400	P>Q	missense
LPS88	*CDK1*	chr10	62545423	C|T	196	66	L>F	splice site
LPS88	*MIER3*	chr5	56247920	C|G	9+1			splice site
LPS88	*RFNG*	chr17	80006685	T|C	915-2			splice site
LPS89	*CDK11A*	chr1	1635511	C|T	1659	553	W>*	stopgain
LPS89	*DPT*	chr1	168698228	A|G	185	62	I>T	missense
LPS89	*CDK18*	chr1	205498506	C|A	1127	376	T>N	missense
LPS89	*POTEF*	chr2	130832515	T|C	2530	844	T>A	missense
LPS89	*FN1*	chr2	216245643	T|G	5325	1775	E>D	missense
LPS89	*FN1*	chr2	216245673	A|C	5295	1765	H>Q	missense
LPS89	*FN1*	chr2	216245674	T|C	5294	1765	H>R	missense
LPS89	*DES*	chr2	220283491	A|G	307	103	T>A	missense
LPS89	*VGLL3*	chr3	87027733	G|A	346	116	L>F	missense
LPS89	*VGLL3*	chr3	87027738	A|T	341	114	I>N	missense
LPS89	*EIF4G1*	chr3	184035250	C|T	28	10	P>S	missense
LPS89	*PRRC2A*	chr6	31602629	G|T	5035	1679	A>S	missense
LPS89	*ZFAND3*	chr6	38029473	A|G	217	73	I>V	missense
LPS89	*CITED2*	chr6	139694661	C|T	421	141	A>T	missense
LPS89	*CITED2*	chr6	139695028	A|G	54	18	N>N	missense
LPS89	*HNRNPA2B1*	chr7	26232975	T|C	896	299	N>S	missense
LPS89	*EXT1*	chr8	119122409	C|A	877	293	V>L	missense
LPS89	*EXT1*	chr8	119123090	C|A	196	66	V>F	missense
LPS89	*EXT1*	chr8	119123142	G|A	144	48	H>H	missense

LPS89	*EXT1*	chr8	119123210	A|C	76	26	L>V	missense
LPS89	*EIF5AL1*	chr10	81272731	C|G	326	109	P>R	missense
LPS89	*ZBED5*	chr11	10875123	G|C	1370	457	S>C	missense
LPS89	*NAV2*	chr11	20066820	T|C	3506	1169	M>T	missense
LPS89	*SF1*	chr11	64532974	C|T	1804	602	G>S	missense
LPS89	*CFL1*	chr11	65623512	C|T	154	52	A>T	missense
LPS89	*CRYAB*	chr11	111779508	T|C	307	103	T>A	missense
LPS89	*DAZAP2*	chr12	51636352	G|C	540	180	L>F	missense
LPS89	*SIX1*	chr14	61113150	C|T	187	63	G>S	missense
LPS89	*ACTN1*	chr14	69347592	C|A	2068	690	G>C	missense
LPS89	*SERF2*	chr15	44085973	T|C	316	106	F>L	missense
LPS89	*SERF2*	chr15	44086004	T|C	347	116	F>S	missense
LPS89	*SERF2*	chr15	44086034	C|T	377	126	T>M	missense
LPS89	*SERF2*	chr15	44086040	C|A	383	128	A>E	missense
LPS89	*SERF2*	chr15	44086046	C|T	389	130	P>L	missense
LPS89	*SERF2*	chr15	44086162	C|T	505	169	R>W	missense
LPS89	*TUBB3*	chr16	90001818	G|C	2000	667	R>P	missense
LPS89	*TUBB3*	chr16	90001863	G|A	2045	682	S>N	missense
LPS89	*RTN4RL1*	chr17	1841070	C|A	46	16	A>S	missense
LPS89	*PFN1*	chr17	4851661	T|C	29	10	N>S	missense
LPS89	*NR1D1,THRA*	chr17	38249442	A|G	1739	580	L>S	missense
LPS89	*MSL1*	chr17	38285619	T|A	1114	372	L>I	missense
LPS89	*COL1A1*	chr17	48272622	C|T	1270	424	G>S	missense
LPS89	*COL1A1*	chr17	48278834	G|C	41	14	A>G	missense
LPS89	*DDX5*	chr17	62496142	G|C	1744	582	P>A	missense
LPS89	*TIMP2*	chr17	76851867	T|C	545	182	N>S	missense
LPS89	*ACTG1*	chr17	79479066	C|T	226	76	V>I	missense
LPS89	*JUNB*	chr19	12903522	A|G	937	313	T>A	missense
LPS89	*ACTN4*	chr19	39216461	A|G	2108	703	N>S	missense
LPS89	*ZNF526*	chr19	42729027	C|T	472	158	H>Y	missense
LPS89	*MYH9*	chr22	36708183	T|C	1639	547	M>V	missense
LPS89	*H1F0*	chr22	38201955	C|G	404	135	T>S	missense
LPS89	*SLC35A2*	chrX	48762228	C|G	958	320	V>L	missense
LPS89	*MAGED1*	chrX	51644764	C|A	2243	748	T>N	missense
LPS89	*MSN*	chrX	64958927	G|T	1440	480	Q>H	missense
LPS89	*FHL1*	chrX	135290664	T|G	600	200	D>E	missense
LPS89	*TUBA1B*	chr12	49523175	T|G				splice site
LPS90	*EPHB2*	chr1	23234569	G|A	2260	754	V>I	missense
LPS90	*OBSCN*	chr1	228404205	C|G	2179	727	L>V	missense
LPS90	*TFCP2L1*	chr2	121995220	G|A	982	328	R>W	missense
LPS90	*TWF2*	chr3	52263763	T|A	377	126	H>L	missense
LPS90	*PARP15*	chr3	122340429	C|T	824	275	T>I	missense
LPS90	*CPEB2*	chr4	15005408	G|A	1111	371	A>T	missense
LPS90	*SCD5*	chr4	83626456	C|T	343	115	A>T	missense
LPS90	*ELFN1*	chr7	1786659	C|G	2427	809	D>E	missense
LPS90	*PCLO*	chr7	82580755	C|T	9149	3050	R>Q	missense
LPS90	*UBR5*	chr8	103305986	G|C	4418	1473	A>G	missense
LPS90	*OR52B4*	chr11	4389065	C|A	461	154	S>I	missense
LPS90	*PTGDR2*	chr11	61503043	C|T	440	147	A>V	missense
LPS90	*DAGLA*	chr12	9847451	A|T	1139	380	T>M	missense
LPS90	*CLEC2D*	chr12	49227113	C|T	557	186	K>I	missense
LPS90	*DDX23*	chr12	49227116	C|T	1750	584	D>N	missense
LPS90	*DDX23*	chr12	49418470	G|A	1747	583	E>K	missense

LPS90	*MLL2*	chr12	49420064	G|A	15943	5315	Q>*	stopgain
LPS90	*MLL2*	chr12	57865027	C|T	15685	5229	R>C	missense
LPS90	*GLI1*	chr12	58025065	C|T	2504	835	A>V	missense
LPS90	*PITPNM2*	chr14	105609195	C|A	1634	545	S>F	missense
LPS90	*JAG2*	chr15	28377338	G|A	3554	1185	R>L	missense
LPS90	*HERC2*	chr16	336886	G|A	12478	4160	L>F	missense
LPS90	*PDIA2*	chr16	31392245	G|T	1474	492	D>N	missense
LPS90	*ITGAX*	chr19	39001336	C|A	3304	1102	V>F	missense
LPS90	*RYR1*	chr19	54544231	G|A	9037	3013	H>N	missense
LPS90	*VSTM1*	chr22	45127612	C|A	695	232	A>V	missense
LPS90	*PRR5*	chr22	50845269	A|T	394	132	Q132K	missense

False call validated by Sanger sequencing
True call validated by Sanger Sequencing

Supplementary Table S6: List of genes for targeted capture in Prevalence cohort study

Gene symbol		Gene symbol		Gene symbol		Gene symbol		Gene symbol
53BP1		*COG5*		*FMR1NB*		*NRAS*		*ST8SIA3*
ABCC3		*COL2A1*		*FOLR2*		*OR2A14*		*STAC2*
ADAMTS4		*CPM*		*FRG1*		*OR5M3*		*STARD3*
ADAMTSL3		*CSMD2*		*GLI1*		*OR6B1*		*STAT1*
ADH6		*CSMD3*		*GOLIM4*		*OR8H2*		*STAT3*
AGAP2		*CYP2C19*		*GPR109A*		*OTOP3*		*STAT5*
AKT		*CYTIP*		*HDAC1*		*PADI2*		*STAT6*
ANKS3		*DARS2*		*HDGFRP3*		*PARP1*		*STOX1*
AP-1		*DAZAP2*		*HEG1*		*PCDH15*		*STXBP3*
AP-1		*DEFB125*		*HEPHL1*		*PDE1C*		*SVIL*
AP-1		*DEFB136*		*HER2*		*PDE3A*		*SYT6*
APC		*DLG2*		*HER3*		*PGF*		*TECTB*
APC		*DLG2*		*HOXA5*		*PI3K*		*TEL2*
ARAF		*DLG4*		*HSD17B2*		*PI3K*		*TEL2*
ASAM		*DNAAF1*		*HTR1F*		*PI3K*		*TEP1*
ASB9		*DNAH14*		*INTS2*		*PKC*		*TEP1*
ATF1		*DNAH17*		*INTS6*		*PLBD1*		*THSD7A*
ATF1		*DNAJB3*		*IQGAP1*		*PLCB3*		*TP53*
ATM		*DNAJC14*		*IRF2*		*PLCB4*		*TP53TG5*
ATR		*DOCK4*		*ITSN2*		*PLEC*		*TPP2*
ATR		*DOCK7*		*KCNK10*		*PLIN2*		*TRIM32*
ATR		*DOPEY1*		*KDM5B*		*POLE*		*TSN*
ATR		*DPP6*		*KIAA1033*		*PPARG*		*TSR2*
B2M		*DRGX*		*KIT*		*PPFIA2*		*TTI1*
BRAF		*DST*		*KRAS*		*PPP2R1B*		*TTI2*
BRCA1		*E2F1*		*KRTAP10-11*		*PRKD3*		*UBQLNL*
BRCA2		*EBF3*		*LEP*		*PTEN*		*USP34*
BTBD7		*EBF3*		*LIPE*		*PTPN9*		*USP48*
C4ORF41		*EGF*		*LPL*		*PTPRD*		*VEGFA*
C6ORF138		*EGFR*		*LPL*		*PUM1*		*VEGFB*
CACNA1D		*EI24*		*LY75*		*RAD51*		*VEPH1*
CADM1		*ERBB4*		*MAGEB6*		*RAF*		*WDR66*
CADM2		*ERCC1*		*MAPKAP1*		*RB*		*WDR96*
CAMKK2		*ERCC4*		*MCM6*		*RCBTB1*		*WDSUB1*
CCDC93		*ERCC5*		*MCTS1*		*RNF182*		*WFS1*
CDH1		*ERK1*		*MDC1*		*RNF32*		*XIRP2*
CDH1		*ERK2*		*MGAM*		*ROBO1*		*XRCC1*
CDH11		*ERK3*		*MGAT3*		*ROS1*		*ZBTB16*
CDH12		*ERK3*		*MGAT3*		*RPUSD2*		*ZBTB38*
CDKN2A		*ERK3*		*MIA2*		*RYR2*		*ZDHHC4*
CDKN2B		*ERK5*		*MTDH*		*SACS*		*ZMYM4*
CEBPA		*FAM47C*		*MTOR*		*SEC63*		*ZNF135*
CEBPB		*FANCA*		*MUC12*		*SEMA3A*		*ZNF169*

CFD		*FANCB*		*MUC4*		*SEMA3G*		*ZNF280C*
CFD		*FANCB*		*MXRA5*		*SEZ6L*		*ZNF611*
CGNL1		*FANCD2*		*MYH3*		*SLC5A3*		*ZNF75D*
CHD6		*FAT3*		*MYO3A*		*SLITRK2*		
CHEK1		*FCAMR*		*NDUFA9*		*SOX5*		
CHEK2		*FER1L6*		*NEUROD4*		*SPHKAP*		
COG1		*FLT1*		*NF1*		*SPOCK3*		

Supplementary Table S7. Coverage analysis of targeted exome sequencing of LPS patients and LPS cell lines in Prevalence cohort

Sample ID	Sample type	Bait set	Genome size	Bait size	Mean target coverage	Target coverage >20x
LPS1	Tumor	SureSelect customized beads	3095693983	2027742	106	90%
LPS11	Germline	SureSelect customized beads	3095693983	2027742	113	86%
LPS11	Tumor	SureSelect customized beads	3095693983	2027742	113	86%
LPS12	Germline	SureSelect customized beads	3095693983	2027742	75	67%
LPS12	Tumor	SureSelect customized beads	3095693983	2027742	141	82%
LPS14	Tumor	SureSelect customized beads	3095693983	2027742	156	91%
LPS15	Tumor	SureSelect customized beads	3095693983	2027742	163	90%
LPS16	Tumor	SureSelect customized beads	3095693983	2027742	89	78%
LPS19	Germline	SureSelect customized beads	3095693983	2027742	143	94%
LPS19	Tumor	SureSelect customized beads	3095693983	2027742	48	53%
LPS2	Tumor	SureSelect customized beads	3095693983	2027742	111	87%
LPS20	Tumor	SureSelect customized beads	3095693983	2027742	104	78%
LPS21	Tumor	SureSelect customized beads	3095693983	2027742	248	97%
LPS22	Germline	SureSelect customized beads	3095693983	2027742	147	94%
LPS22	Tumor	SureSelect customized beads	3095693983	2027742	87	60%
LPS23	Tumor	SureSelect customized beads	3095693983	2027742	124	86%
LPS24	Tumor	SureSelect customized beads	3095693983	2027742	228	96%
LPS25	Tumor	SureSelect customized beads	3095693983	2027742	31	38%
LPS26	Tumor	SureSelect customized beads	3095693983	2027742	105	88%
LPS27	Tumor	SureSelect customized beads	3095693983	2027742	60	55%
LPS28	Tumor	SureSelect customized beads	3095693983	2027742	63	69%
LPS29	Tumor	SureSelect customized beads	3095693983	2027742	130	86%
LPS3	Tumor	SureSelect customized beads	3095693983	2027742	134	92%
LPS30	Tumor	SureSelect customized beads	3095693983	2027742	77	64%
LPS31	Tumor	SureSelect customized beads	3095693983	2027742	45	50%
LPS32	Tumor	SureSelect customized beads	3095693983	2027742	24	31%
LPS33	Tumor	SureSelect customized beads	3095693983	2027742	87	78%
LPS34	Tumor	SureSelect customized beads	3095693983	2027742	30	40%
LPS35	Tumor	SureSelect customized beads	3095693983	2027742	86	84%
LPS36	Tumor	SureSelect customized beads	3095693983	2027742	20	26%
LPS37	Tumor	SureSelect customized beads	3095693983	2027742	304	95%
LPS38	Tumor	SureSelect customized beads	3095693983	2027742	119	89%
LPS39	Tumor	SureSelect customized beads	3095693983	2027742	52	59%
LPS4	Tumor	SureSelect customized beads	3095693983	2027742	59	71%
LPS40	Tumor	SureSelect customized beads	3095693983	2027742	161	91%
LPS41	Tumor	SureSelect customized beads	3095693983	2027742	141	93%
LPS42	Tumor	SureSelect customized beads	3095693983	2027742	107	91%
LPS43	Tumor	SureSelect customized beads	3095693983	2027742	151	95%
LPS44	Tumor	SureSelect customized beads	3095693983	2027742	167	94%
LPS45	Tumor	SureSelect customized beads	3095693983	2027742	118	85%
LPS46	Tumor	SureSelect customized beads	3095693983	2027742	145	89%
LPS47	Tumor	SureSelect customized beads	3095693983	2027742	93	82%
LPS48	Tumor	SureSelect customized beads	3095693983	2027742	84	76%
LPS49	Tumor	SureSelect customized beads	3095693983	2027742	149	89%
LPS5	Tumor	SureSelect customized beads	3095693983	2027742	104	90%
LPS50	Tumor	SureSelect customized beads	3095693983	2027742	126	85%
LPS51	Tumor	SureSelect customized beads	3095693983	2027742	69	73%
LPS52	Tumor	SureSelect customized beads	3095693983	2027742	101	82%
LPS53	Tumor	SureSelect customized beads	3095693983	2027742	200	92%
LPS54	Tumor	SureSelect customized beads	3095693983	2027742	11	4%
LPS55	Germline	SureSelect customized beads	3095693983	2027742	132	92%
LPS55	Tumor	SureSelect customized beads	3095693983	2027742	104	80%
LPS57	Tumor	SureSelect customized beads	3095693983	2027742	14	10%
LPS58	Tumor	SureSelect customized beads	3095693983	2027742	27	44%
LPS59	Germline	SureSelect customized beads	3095693983	2027742	111	87%
LPS59	Tumor	SureSelect customized beads	3095693983	2027742	26	34%
LPS6	Tumor	SureSelect customized beads	3095693983	2027742	197	94%
LPS61	Tumor	SureSelect customized beads	3095693983	2027742	20	20%

LPS62	Tumor	SureSelect customized beads	3095693983	2027742	45	54%
LPS63	Tumor	SureSelect customized beads	3095693983	2027742	116	84%
LPS64	Tumor	SureSelect customized beads	3095693983	2027742	118	83%
LPS65	Tumor	SureSelect customized beads	3095693983	2027742	93	85%
LPS66	Tumor	SureSelect customized beads	3095693983	2027742	56	71%
LPS67	Germline	SureSelect customized beads	3095693983	2027742	196	94%
LPS67	Tumor	SureSelect customized beads	3095693983	2027742	66	87%
LPS68	Tumor	SureSelect customized beads	3095693983	2027742	111	85%
LPS69	Tumor	SureSelect customized beads	3095693983	2027742	141	91%
LPS7	Tumor	SureSelect customized beads	3095693983	2027742	101	89%
LPS70	Tumor	SureSelect customized beads	3095693983	2027742	114	88%
LPS71	Tumor	SureSelect customized beads	3095693983	2027742	149	94%
LPS72	Tumor	SureSelect customized beads	3095693983	2027742	184	96%
LPS73	Tumor	SureSelect customized beads	3095693983	2027742	117	94%
LPS74	Tumor	SureSelect customized beads	3095693983	2027742	210	95%
LPS75	Tumor	SureSelect customized beads	3095693983	2027742	54	64%
LPS76	Tumor	SureSelect customized beads	3095693983	2027742	33	50%
LPS77	Tumor	SureSelect customized beads	3095693983	2027742	117	91%
LPS78	Tumor	SureSelect customized beads	3095693983	2027742	92	85%
LPS79	Tumor	SureSelect customized beads	3095693983	2027742	80	77%
LPS8	Tumor	SureSelect customized beads	3095693983	2027742	61	73%
LPS80	Tumor	SureSelect customized beads	3095693983	2027742	195	94%
LPS80	Germline	SureSelect customized beads	3095693983	2027742	174	95%
LPS81	Tumor	SureSelect customized beads	3095693983	2027742	88	84%
LPS82	Tumor	SureSelect customized beads	3095693983	2027742	127	95%
LPS83	Tumor	SureSelect customized beads	3095693983	2027742	187	93%
LPS84	Tumor	SureSelect customized beads	3095693983	2027742	191	95%
LPS85	Tumor	SureSelect customized beads	3095693983	2027742	196	94%
LPS86	Tumor	SureSelect customized beads	3095693983	2027742	230	95%
LPS86	Germline	SureSelect customized beads	3095693983	2027742	208	96%
LPS87	Tumor	SureSelect customized beads	3095693983	2027742	252	95%
LPS87	Germline	SureSelect customized beads	3095693983	2027742	162	94%
LPS88	Germline	SureSelect customized beads	3095693983	2027742	226	96%
LPS88	Tumor	SureSelect customized beads	3095693983	2027742	139	92%
LPS89	Tumor	SureSelect customized beads	3095693983	2027742	281	96%
LPS89	Germline	SureSelect customized beads	3095693983	2027742	140	92%
LPS9	Tumor	SureSelect customized beads	3095693983	2027742	103	77%
LPS90	Tumor	SureSelect customized beads	3095693983	2027742	273	96%
LPS90	Germline	SureSelect customized beads	3095693983	2027742	173	94%
LPS91-T1	Tumor	SureSelect customized beads	3095693983	2027742	1246	98%
LPS91-T2	Tumor	SureSelect customized beads	3095693983	2027742	1045	98%
LPS91-T3	Tumor	SureSelect customized beads	3095693983	2027742	1032	98%
LPS91	Germline	SureSelect customized beads	3095693983	2027742	833	97%
LPS141	Cell Line	SureSelect customized beads	3095693983	2027742	224	94%
LP6	Cell Line	SureSelect customized beads	3095693983	2027742	178	93%
SW872	Cell Line	SureSelect customized beads	3095693983	2027742	166	93%
LP1	Cell Line	SureSelect customized beads	3095693983	2027742	135	86%
LP2	Cell Line	SureSelect customized beads	3095693983	2027742	437	94%
LP3	Cell Line	SureSelect customized beads	3095693983	2027742	491	95%
SA-4	Cell Line	SureSelect customized beads	3095693983	2027742	710	96%
MLS-402	Cell Line	SureSelect customized beads	3095693983	2027742	783	97%
GOT-3	Cell Line	SureSelect customized beads	3095693983	2027742	1010	97%
FU-DDLS-1	Cell Line	SureSelect customized beads	3095693983	2027742	175	88%
LiSa-2	Cell Line	SureSelect customized beads	3095693983	2027742	298	93%
T1000	Cell Line	SureSelect customized beads	3095693983	2027742	351	93%
T1778	Cell Line	SureSelect customized beads	3095693983	2027742	641	96%

Supplementary Table S8. Summary of mutations in the LPS Prevalence Cohort and LPS cell lines

LPS patients

<table>
<tr><th>Sample ID</th><th>Gene Symbol</th><th>Chromosome</th><th>Genomic position</th><th>Nucleotide change (reference|variant)</th><th>cDNA position</th><th>Amino acid position</th><th>Amino acid change</th><th>Mutation type</th></tr>
<tr><td>LPS1</td><td>ADAMTS4</td><td>chr1</td><td>161163466</td><td>C|T</td><td>1699</td><td>567</td><td>R>C</td><td>missense</td></tr>
<tr><td>LPS1</td><td>MUC4</td><td>chr3</td><td>195489792</td><td>G|C</td><td>1417</td><td>473</td><td>D>H</td><td>missense</td></tr>
<tr><td>LPS1</td><td>MUC4</td><td>chr3</td><td>195507790</td><td>C|T</td><td>10661</td><td>3554</td><td>P>L</td><td>missense</td></tr>
<tr><td>LPS1</td><td>MUC4</td><td>chr3</td><td>195516350</td><td>G|A</td><td>2101</td><td>701</td><td>G>R</td><td>missense</td></tr>
<tr><td>LPS1</td><td>MUC4</td><td>chr3</td><td>195509423</td><td>G|A</td><td>9028</td><td>3010</td><td>P>S</td><td>missense</td></tr>
<tr><td>LPS1</td><td>MUC4</td><td>chr3</td><td>195511217</td><td>C|T</td><td>7234</td><td>2412</td><td>G>S</td><td>missense</td></tr>
<tr><td>LPS1</td><td>MUC4</td><td>chr3</td><td>195511889</td><td>C|T</td><td>6562</td><td>2188</td><td>G>S</td><td>missense</td></tr>
<tr><td>LPS1</td><td>MUC4</td><td>chr3</td><td>195511985</td><td>C|T</td><td>6466</td><td>2156</td><td>G>S</td><td>missense</td></tr>
<tr><td>LPS1</td><td>MUC4</td><td>chr3</td><td>195507424</td><td>C|G</td><td>11027</td><td>3676</td><td>G>A</td><td>missense</td></tr>
<tr><td>LPS1</td><td>MUC4</td><td>chr3</td><td>195510785</td><td>C|T</td><td>7666</td><td>2556</td><td>G>S</td><td>missense</td></tr>
<tr><td>LPS1</td><td>MUC4</td><td>chr3</td><td>195508111</td><td>G|A</td><td>10340</td><td>3447</td><td>S>F</td><td>missense</td></tr>
<tr><td>LPS1</td><td>MUC4</td><td>chr3</td><td>195508324</td><td>C|T</td><td>10127</td><td>3376</td><td>R>H</td><td>missense</td></tr>
<tr><td>LPS1</td><td>DST</td><td>chr6</td><td>56462690</td><td>A|T</td><td>4174</td><td>1392</td><td>L>F</td><td>missense</td></tr>
<tr><td>LPS1</td><td>MUC12</td><td>chr7</td><td>100635210</td><td>T|C</td><td>1366</td><td>456</td><td>S>P</td><td>missense</td></tr>
<tr><td>LPS1</td><td>CSMD3</td><td>chr8</td><td>113988320</td><td>C|G</td><td>1088</td><td>363</td><td>T>S</td><td>missense</td></tr>
<tr><td>LPS1</td><td>HEPHL1</td><td>chr11</td><td>93806252</td><td>C|T</td><td>1294</td><td>432</td><td>R>W</td><td>missense</td></tr>
<tr><td>LPS1</td><td>CGNL1</td><td>chr15</td><td>57809168</td><td>C|T</td><td>2594</td><td>865</td><td>T>M</td><td>missense</td></tr>
<tr><td>LPS1</td><td>DNAAF1</td><td>chr16</td><td>84203737</td><td>G|A</td><td>1301</td><td>435</td><td>D>N</td><td>missense</td></tr>
<tr><td>LPS1</td><td>PLCB4</td><td>chr20</td><td>9424868</td><td>C|A</td><td>2822</td><td>941</td><td>S>Y</td><td>missense</td></tr>
<tr><td>LPS2</td><td>PIKFYVE</td><td>chr2</td><td>209190458</td><td>G|A</td><td>2932</td><td>975</td><td>V>I</td><td>missense</td></tr>
<tr><td>LPS2</td><td>MUC4</td><td>chr3</td><td>195516350</td><td>G|A</td><td>2101</td><td>701</td><td>G>R</td><td>missense</td></tr>
<tr><td>LPS2</td><td>LPL</td><td>chr8</td><td>19809344</td><td>A|C</td><td>314</td><td>105</td><td>D>A</td><td>missense</td></tr>
<tr><td>LPS2</td><td>CSMD3</td><td>chr8</td><td>113299353</td><td>T|C</td><td>9271</td><td>3091</td><td>S>P</td><td>missense</td></tr>
<tr><td>LPS2</td><td>UBQLNL</td><td>chr11</td><td>5536386</td><td>T|C</td><td>1286</td><td>429</td><td>M>T</td><td>missense</td></tr>
<tr><td>LPS2</td><td>ATM</td><td>chr11</td><td>108201015</td><td>G|A</td><td>7382</td><td>2461</td><td>R>H</td><td>missense</td></tr>
<tr><td>LPS2</td><td>POLE</td><td>chr12</td><td>133226360</td><td>G|A</td><td>3698</td><td>1233</td><td>R>Q</td><td>missense</td></tr>
<tr><td>LPS2</td><td>MAPK6</td><td>chr15</td><td>52339208</td><td>A|G</td><td>551</td><td>184</td><td>H>R</td><td>missense</td></tr>
<tr><td>LPS2</td><td>FANCA</td><td>chr16</td><td>89805600</td><td>G|T</td><td>4108</td><td>1370</td><td>A>S</td><td>missense</td></tr>
<tr><td>LPS2</td><td>NF1</td><td>chr17</td><td>29653037</td><td>A|G</td><td>5035</td><td>1679</td><td>I>V</td><td>missense</td></tr>
<tr><td>LPS2</td><td>STARD3</td><td>chr17</td><td>37818557</td><td>G|A</td><td>1139</td><td>380</td><td>R>H</td><td>missense</td></tr>
<tr><td>LPS2</td><td>ABCC3</td><td>chr17</td><td>48753731</td><td>C|T</td><td>3160</td><td>1054</td><td>R>C</td><td>missense</td></tr>
<tr><td>LPS2</td><td>PPP2R1B</td><td>chr11</td><td>111624198</td><td>AGA|□</td><td>750_752</td><td>250_251</td><td>del</td><td>nonframeshift deletion</td></tr>
<tr><td>LPS2</td><td>SOX5</td><td>chr12</td><td>23757462</td><td>T|A</td><td>1023</td><td>341</td><td>L>F</td><td>missense</td></tr>
<tr><td>LPS2</td><td>AGAP2</td><td>chr12</td><td>58120773</td><td>G|A</td><td>2252</td><td>751</td><td>T>M</td><td>missense</td></tr>
<tr><td>LPS2</td><td>IQGAP1</td><td>chr15</td><td>90972822</td><td>C|T</td><td>314</td><td>105</td><td>A>V</td><td>missense</td></tr>
<tr><td>LPS2</td><td>STARD3</td><td>chr17</td><td>37815324</td><td>GAA|-</td><td>613_615</td><td>205_205</td><td>del</td><td>nonframeshift deletion</td></tr>
<tr><td>LPS2</td><td>STARD3</td><td>chr17</td><td>37815326</td><td>A|T</td><td>615</td><td>205</td><td>E>D</td><td>missense</td></tr>
<tr><td>LPS2</td><td>STARD3</td><td>chr17</td><td>37815328</td><td>A|T</td><td>617</td><td>206</td><td>E>V</td><td>missense</td></tr>
<tr><td>LPS2</td><td>STARD3</td><td>chr17</td><td>37815329</td><td>A|T</td><td>618</td><td>206</td><td>E>D</td><td>missense</td></tr>
<tr><td>LPS3</td><td>MUC4</td><td>chr3</td><td>195513169</td><td>T|C</td><td>5282</td><td>1761</td><td>L>P</td><td>missense</td></tr>
<tr><td>LPS3</td><td>FRG1</td><td>chr4</td><td>190876263</td><td>A|T</td><td>389</td><td>130</td><td>D>V</td><td>missense</td></tr>
<tr><td>LPS3</td><td>PCDH15</td><td>chr10</td><td>55892652</td><td>G|A</td><td>1900</td><td>634</td><td>V>I</td><td>missense</td></tr>
<tr><td>LPS3</td><td>HEPHL1</td><td>chr11</td><td>93806252</td><td>C|T</td><td>1294</td><td>432</td><td>R>W</td><td>missense</td></tr>
<tr><td>LPS3</td><td>SACS</td><td>chr13</td><td>23928845</td><td>C|T</td><td>1906</td><td>636</td><td>R>W</td><td>missense</td></tr>
<tr><td>LPS3</td><td>TP53BP1</td><td>chr15</td><td>43748962</td><td>C|T</td><td>1844</td><td>615</td><td>T>M</td><td>missense</td></tr>
<tr><td>LPS3</td><td>TP53BP1</td><td>chr15</td><td>43784487</td><td>G|T</td><td>187</td><td>63</td><td>V>L</td><td>missense</td></tr>
<tr><td>LPS3</td><td>DNAAF1</td><td>chr16</td><td>84203737</td><td>G|A</td><td>1303</td><td>435</td><td>D>N</td><td>missense</td></tr>
<tr><td>LPS3</td><td>FANCA</td><td>chr16</td><td>89865593</td><td>C|G</td><td>874</td><td>292</td><td>H>D</td><td>missense</td></tr>
<tr><td>LPS3</td><td>INTS2</td><td>chr17</td><td>60002371</td><td>C|T</td><td>407</td><td>136</td><td>P>L</td><td>missense</td></tr>
<tr><td>LPS3</td><td>PCDH15</td><td>chr10</td><td>55892652</td><td>C|T</td><td>1789</td><td>597</td><td>V>I</td><td>missense</td></tr>
<tr><td>LPS3</td><td>SACS</td><td>chr13</td><td>23906592</td><td>G|A</td><td>11423</td><td>3808</td><td>P>L</td><td>missense</td></tr>
<tr><td>LPS3</td><td>FAT3</td><td>chr11</td><td>92087318</td><td>C|A</td><td>2040</td><td>680</td><td>S>R</td><td>missense</td></tr>
</table>

LPS3	*MUC4*	chr3	195509423	G|A	9028	3010	P>S	missense
LPS3	*ADAMTSL3*	chr15	84506946	T|G	706	236	S>A	missense
LPS3	*MXRA5*	chrX	3240529	G|A	3197	1066	T>I	missense
LPS3	*KIT*	chr4	55602911	C|T	2621	874	P>L	missense
LPS3	*EGF*	chr4	110920885	G|C	2933	978	W>S	missense
LPS3	*EGFR*	chr7	55225397	C|T	1249	417	L>F	missense
LPS3	*MUC4*	chr3	195508324	C|T	10127	3376	R>H	missense
LPS3	*ZNF280C*	chrX	129377604	G|A	314	105	S>L	missense
LPS3	*NF1*	chr17	29553477	|C	2026_2027	676	T>fs	frameshift insertion
LPS4	*APC*	chr5	112174509	C|T	3164	1055	T>I	missense
LPS4	*APC*	chr5	112178555	A|T	7210	2404	T>S	missense
LPS4	*MOGAT3*	chr7	100839291	A|T	962	321	Q>L	missense
LPS4	*MXRA5*	chrX	3248104	G|A	664	222	D>N	missense
LPS4	*AKT1*	chr14	105239269	G|A	1118	373	G>D	missense
LPS4	*TP53BP1*	chr15	43748426	G|A	2380	794	D>N	missense
LPS4	*DNAAF1*	chr16	84193330	C|A	792	264	Y>X	stopgain
LPS4	*CHD6*	chr20	40068692	T|A	3955	1319	S>T	missense
LPS4	*DOCK4*	chr7	111644135	C|G	89	30	G>A	missense
LPS4	*COL2A1*	chr12	48378375	G|T	1634	545	P>H	missense
LPS5	*XIRP2*	chr2	168106976	C|T	8404	2803	S>F	missense
LPS5	*MUC4*	chr3	195498671	A|C	233	78	Q>P	missense
LPS5	*MUC4*	chr3	195510914	C|G	7537	2513	L>V	missense
LPS5	*CDH12*	chr5	21975353	T|C	373	125	Y>H	missense
LPS5	*MXRA5*	chrX	3228382	C|T	7862	2621	A>V	missense
LPS5	*MXRA5*	chrX	3240898	G|A	2828	943	G>D	missense
LPS5	*FAT3*	chr11	92257962	T|C	3455	1152	I>T	missense
LPS5	*MUC12*	chr7	100643362	C|G	9518	3173	T>R	missense
LPS5	*STAT6*	chr12	57500533	C|T	91	31	V>I	missense
LPS5	*GLI1*	chr12	57861822	G|A	739	247	D>N	missense
LPS5	*MYH3*	chr17	10547954	G|A	1207	403	P>S	missense
LPS5	*TTI1*	chr20	36642026	G|C	193	65	P>A	missense
LPS6	*PIK3CD*	chr1	9775972	T|A	436	146	F>I	missense
LPS6	*MUC4*	chr3	195506159	C|G	12292	4098	P>A	missense
LPS6	*MUC12*	chr7	100634887	G|A	1043	348	R>H	missense
LPS6	*MUC12*	chr7	100638976	A|G	5132	1711	E>G	missense
LPS6	*SVIL*	chr10	29773588	C|G	3674	1225	P>R	missense
LPS6	*GLI1*	chr12	57863433	C|T	1528	510	R>W	missense
LPS6	*TP53BP1*	chr15	43739624	G|A	2776	926	A>T	missense
LPS6	*DNAAF1*	chr16	84199444	C|G	919	307	Q>E	missense
LPS6	*HEPHL1*	chr11	93822135	G|T		splicing	splicing	splicing
LPS6	*XIRP2*	chr2	168100497	G|-	1929	643	E>fs	frameshift deletion
LPS7	*PIKFYVE*	chr2	209190458	G|A	2923	975	V>I	missense
LPS7	*MUC4*	chr3	195516350	G|A	2101	701	G>R	missense
LPS7	*LPL*	chr8	19809344	A|C	314	105	D>A	missense
LPS7	*CSMD3*	chr8	113299353	T|C	9271	3091	S>P	missense
LPS7	*MXRA5*	chrX	3238612	A|G	5114	1705	N>S	missense
LPS7	*UBQLNL*	chr11	5536386	T|C	1286	429	M>T	missense
LPS7	*ATM*	chr11	108201015	G|A	7382	2461	R>H	missense
LPS7	*MAPK6*	chr15	52339208	A|G	551	184	H>R	missense
LPS7	*FANCA*	chr16	89805600	G|T	4108	1370	A>S	missense
LPS7	*NF1*	chr17	29653037	A|G	5035	1679	I>V	missense
LPS7	*STARD3*	chr17	37818557	G|A	1139	380	R>H	missense
LPS7	*ABCC3*	chr17	48746804	G|A	2156	719	R>H	missense
LPS7	*ABCC3*	chr17	48753731	C|T	3160	1054	R>C	missense
LPS7	*ZBTB38*	chr3	141161560	A|-	330	110	A>fs	frameshift deletion
LPS7	*PPP2R1B*	chr11	111624198	AGA|-	750_752	250_251	del	nonframeshift deletion
LPS7	*STARD3*	chr17	37815324	GAA|-	613_615	205_205	del	nonframeshift deletion
LPS7	*CACNA1D*	chr3	53778823	T|C	2975	992	L>P	missense
LPS7	*ZBTB38*	chr3	141161558	G|A	328	110	A>T	missense
LPS7	*ZBTB38*	chr3	141161565	A|G	335	112	K>R	missense
LPS7	*RNF182*	chr6	13977472	A|T	122	41	H>L	missense
LPS7	*PLCB3*	chr11	64029920	G|T	1879	627	G>W	missense

LPS7	*STARD3*	chr17	37815326	A|T	615	205	E>D	missense
LPS7	*STARD3*	chr17	37815328	A|T	617	206	E>V	missense
LPS7	*ZNF280C*	chrX	129349808	G|A	1795	599	R>C	missense
LPS8	*USP34*	chr2	61447507	A|G	7985	2662	Q>R	missense
LPS8	*MUC4*	chr3	195508228	C|G	10223	3408	P>R	missense
LPS8	*MUC4*	chr3	195508300	C|T	10151	53384	S>L	missense
LPS8	*MUC12*	chr7	100636285	C|T	2441	814	T>I	missense
LPS8	*ANKS3*	chr16	4777017	G|T	332	5111	S>I	missense
LPS8	*MUC12*	chr7	100635082	A|T	1238	413	H>L	missense
LPS8	*MUC12*	chr7	100645780	C|T	11936	3979	A>V	missense
LPS8	*FLT1*	chr13	28886138	C|A	3484	1162	V>L	missense
LPS8	*ANKS3*	chr16	4777017	C|A	332	111	S>I	missense
LPS9	*KDM5B*	chr1	202698950	G|A	4382	1461	R>H	missense
LPS9	*XIRP2*	chr2	168106631	T|C	8063	2688	F>S	missense
LPS9	*MUC4*	chr3	195506060	G|C	12391	4131	V>L	missense
LPS9	*MUC4*	chr3	195506303	C|A	12148	4050	P>T	missense
LPS9	*MUC4*	chr3	195508453	G|A	9998	3333	S>N	missense
LPS9	*MUC4*	chr3	195510073	C|T	8378	2793	A>V	missense
LPS9	*MUC4*	chr3	195510107	G|A	8344	2782	A>T	missense
LPS9	*MUC4*	chr3	195512480	T|C	5971	1991	S>P	missense
LPS9	*PLEC*	chr8	145009036	G|A	899	300	R>Q	missense
LPS9	*ZNF75D*	chrX	134421668	C|T	649	217	Q>X	missense
LPS9	*TP53*	chr17	7579698					
LPS11	*PADI2*	chr1	17409049	C|A	1149	383	K>N	missense
LPS11	*WDR96*	chr10	105923977	C|T	3121	1041	V>M	missense
LPS11	*FOLR2*	chr11	71932639	G|-	601	201	G>fs	frameshift deletion
LPS11	*PLBD1*	chr12	14706266	C|T	196	66	A>T	missense
LPS11	*DNAJC14*	chr12	56221343	G|C	1100	367	S>C	missense
LPS11	*MYH3*	chr17	10552985	C|T	551	184	G>E	missense
LPS11	*INTS2*	chr17	59955334	G|C	2394	798	S>R	missense
LPS11	*DNAH17*	chr17	76497874	T|G	5272	1758	T>P	missense
LPS11	*SPHKAP*	chr2	228890137	A|T	414	138	N>K	missense
LPS11	*ZBTB38*	chr3	141164779	G|T	3549	1183	K>N	missense
LPS11	*MDC1*	chr6	30673497	C|G	3463	1155	V>L	missense
LPS11	*MUC12*	chr7	100647851	C|G	14007	4669	H>Q	missense
LPS11	*OR6B1*	chr7	143701831	A|G	742	248	I>V	missense
LPS11	*PRKD3*	chr2	37543624	A|C	44	15	L>X	stopgain SNV
LPS11	*STXBP3*	chr1	109350028	G|A	1541	514	R>H	missense
LPS11	*VEPH1*	chr3	157178123	T|-	376	126	M>fs	frameshift deletion
LPS11	*OTOP3*	chr17	72937649	C|T	235	79	R>W	missense
LPS11	*EBF3*	chr10	131676052	G|C	616	206	R>G	missense
LPS11	*MXRA5*	chrX	3242785	G|A	941	314	P>L	missense
LPS11	*FRG1*	chr4	190878646	G|T	526	176	E>X	stopgain SNV
LPS11	*DOCK4*	chr7	111508192	|A	2128_2129	710_711	K>delinsX	stopgain SNV
LPS11	*ERCC4*	chr16	14038581	|A	1906_1907	636	E>fs	frameshift insertion
LPS11	*INTS6*	chr13	51957533	C|A	1112	371	S>I	missense
LPS11	*EGF*	chr4	110895945	C|A	1685	562	A>D	missense
LPS11	*BRCA2*	chr13	32913920	G|C	5428	1810	V>L	missense
LPS11	*USP34*	chr2	61510584	T|A	4833	1611	R>S	missense
LPS11	*DST*	chr6	56392316	G|A	10028	3343	A>V	missense
LPS11	*KDM5B*	chr1	202704682	C|T	3298	1100	G>R	missense
LPS11	*FOLR2*	chr11	71932290	G|T	410	137	W>L	missense
LPS11	*TP53BP1*	chr15	43762174	T|G	1271	424	N>T	missense
LPS14	*DOCK7*	chr1	63084396	G|A	1663	555	V>I	missense
LPS14	*APC*	chr5	112175363	G|A	4018	1340	A>T	missense
LPS14	*CYP2C18*	chr10	96447920	C|T	370	124	R>W	missense
LPS14	*OR8H2*	chr11	55872943	T|C	425	142	L>P	missense
LPS14	*POLE*	chr12	133254193	C|T	691	231	R>C	missense
LPS14	*MAPK6*	chr15	52339069	C|T	412	138	R>W	missense
LPS14	*ADAMTSL3*	chr15	84651057	G|A	2677	893	E>K	missense
LPS14	*HDAC10*	chr22	50687800	G|A	647	216	R>Q	missense
LPS15	*CSMD2*	chr1	34034977	C|T	8134	2712	L>F	missense
LPS15	*RYR2*	chr1	237619982	G|T	1559	520	R>L	missense

LPS15	*MUC4*	chr3	195508078	C|T	10373	3458	P>L	missense
LPS15	*MUC4*	chr3	195508111	C|T	10340	3447	S>F	missense
LPS15	*MUC4*	chr3	195508307	A|G	10144	3382	I>V	missense
LPS15	*MUC4*	chr3	195509737	T|C	8714	2905	V>A	missense
LPS15	*MUC4*	chr3	195513539	G|C	4912	1638	A>P	missense
LPS15	*MUC4*	chr3	195513541	A|G	4910	1637	N>S	missense
LPS15	*MUC4*	chr3	195513806	C|T	4645	1549	D>H	missense
LPS15	*MUC4*	chr3	195513817	C|T	4634	1545	A>V	missense
LPS15	*MUC4*	chr3	195515510	T|G	2941	981	Y>D	missense
LPS15	*FRG1*	chr4	190876263	A|T	389	130	D>V	missense
LPS15	*DST*	chr6	56499300	G|A	1843	615	V>I	missense
LPS15	*SEMA3A*	chr7	83634712	G|A	1301	435	V>I	missense
LPS15	*MGAM*	chr7	141752757	C|G	3132	1044	Y>X	missense
LPS15	*MGAM*	chr7	141763311	C|A	4270	1424	P>T	missense
LPS15	*MTDH*	chr8	98731338	G|A	1442	481	R>H	missense
LPS15	*MXRA5*	chrX	3229345	G|A	6899	2300	R>H	missense
LPS15	*DLG2*	chr11	83641521	C|T	1148	383	P>L	missense
LPS15	*FAT3*	chr11	92086828	T|C	1550	517	L>S	missense
LPS15	*STAT6*	chr12	57499995	G|A	659	220	S>N	missense
LPS15	*RPUSD2*	chr15	40866186	A|C	1364	455	E>A	missense
LPS15	*DNAAF1*	chr16	84203737	G|A	1303	435	D>N	missense
LPS15	*MYH3*	chr17	10541497	G|A	3592	1198	A>T	missense
LPS15	*DNAH17*	chr17	76437187	A|G	11623	3875	I>V	missense
LPS15	*CHD6*	chr20	40049450	A|T	5825	1942	H>L	missense
LPS16	*DARS2*	chr1	173826791	A|G	1886	629	Y>C	missense
LPS16	*MUC4*	chr3	195508867	C|T	9584	3195	T>I	missense
LPS16	*MUC4*	chr3	195515297	G|C	3154	1052	G>R	missense
LPS16	*GDNF*	chr5	37816042	G|A	398	133	R>Q	missense
LPS16	*MGAM*	chr7	141752215	A|C	2927	976	E>A	missense
LPS16	*ZNF169*	chr9	97063449	T|A	1609	537	S>T	missense
LPS16	*MXRA5*	chrX	3261745	C|T	130	44	R>X	stopgain
LPS16	*OR8H2*	chr11	55872943	T|C	425	142	L>P	missense
LPS16	*FAT3*	chr11	92620184	C|T	12956	4319	P>L	missense
LPS20	*ROBO1*	chr3	78663873	G|T	4060	1354	V>L	missense
LPS20	*MUC4*	chr3	195507221	C|T	11230	3744	P>S	missense
LPS20	*MUC4*	chr3	195508226	C|G	10225	3409	L>V	missense
LPS20	*MUC4*	chr3	195508228	C|G	10223	3408	P>R	missense
LPS20	*MUC4*	chr3	195508777	T|C	9672	3225	V>A	missense
LPS20	*MUC4*	chr3	195508786	T|C	9665	3222	L>P	missense
LPS20	*MUC4*	chr3	195508787	C|A	9664	3222	L>I	missense
LPS20	*SPOCK3*	chr4	168155233	G|A	92	31	G>E	missense
LPS20	*MUC12*	chr7	100634145	G|A	301	101	A>T	missense
LPS20	*PLEC*	chr8	145007513	C|T	1282	428	R>W	missense
LPS20	*MYO3A*	chr10	26482160	A|G	4465	1489	I>V	missense
LPS20	*SVIL*	chr10	29777566	G|A	3034	1012	V>M	missense
LPS20	*PLCB3*	chr11	64027666	A|C	1691	564	E>A	missense
LPS20	*POLE*	chr12	133226017	C|T	3880	1294	R>C	missense
LPS20	*CGNL1*	chr15	57731293	C|T	1096	366	L>F	missense
LPS21	*DNAH14*	chr1	225380461	G|C	5668	180	E>Q	missense
LPS21	*ROBO1*	chr3	78663873	G|T	4060	1354	V>L	missense
LPS21	*PIK3CA*	chr3	178937447	G|A	1835	612	R>Q	missense
LPS21	*MUC4*	chr3	195508385	G|T	10066	3356	G>C	missense
LPS21	*MUC4*	chr3	195508786	T|C	9665	3222	L>P	missense
LPS21	*MUC4*	chr3	195508787	C|A	9664	3222	L>I	missense
LPS21	*MUC4*	chr3	195512761	C|A	5690	1897	A>E	missense
LPS21	*OR8H2*	chr11	55872943	T|C	425	142	L>P	missense
LPS21	*PDE3A*	chr12	20782958	A|G	691	231	T>A	missense
LPS21	*AKT1*	chr14	105246551	G|A	49	17	E>K	missense
LPS21	*LLGL1*	chr17	18145527	C|T	2930	977	P>L	missense
LPS21	*ABCC3*	chr17	48753391	A|G	3007	1003	S>G	missense
LPS21	*LIPE*	chr19	42930480	C|G	822	274	Y>X	stopgain
LPS21	*HDAC10*	chr22	50687800	G|A	647	216	R>Q	missense
LPS21	*MUC4*	chr3	195509423	G|A	9028	3010	P>S	missense
LPS22	*STOX1*	chr10	70645656	G|T	2104	702	D>Y	missense
LPS22	*OR5M3*	chr11	56237775	C|A	199	67	V>F	missense
LPS22	*FAT3*	chr11	92532456	G|A	6277	2093	V>I	missense

LPS22	*MIA2*	chr14	39716604	C|T	826	276	H>Y	missense
LPS22	*ANKS3*	chr16	4777074	C|T	275	92	G>E	missense
LPS22	*STAC2*	chr17	37369376	C|G	1003	335	G>R	missense
LPS22	*ST8SIA3*	chr18	55024338	G|A	497	166	G>E	missense
LPS22	*ZNF135*	chr19	58578553	G|A	737	246	R>Q	missense
LPS22	*PIK3CA*	chr3	178952072	A|G	3127	1043	M>V	missense
LPS22	*DEFB136*	chr8	11831558	A|T	125	42	F>Y	missense
LPS22	*ITSN2*	chr2	24426580	C|G	4928	1643	R>P	missense
LPS22	*HEG1*	chr3	124728625	C|T	3117	1039	M>I	missense
LPS22	*FAT3*	chr11	92531611	TT|-	5432_5433	1811_1811	>del	frameshift deletion
LPS22	*STOX1*	chr10	70644696	|A	1144_1145	382	Q>fs	frameshift insertion
LPS22	*SPOCK3*	chr4	167921546	C|T	37	13	D>N	missense
LPS22	*ITSN2*	chr2	24533386	C|A	528	176	Q>6H	missense
LPS22	*EGFR*	chr7	55273243	G|A	3566	1189	G>D	missense
LPS22	*TRIM32*	chr9	119460427	C|A	406	136	L>I	missense
LPS22	*XRCC1*	chr19	44057791	|G	459_460	153	P>fs	frameshift insertion
LPS22	*HEPHL1*	chr11	93754648	G|T	114	38	W>C	missense
LPS22	*STAT6*	chr12	57490894	|G	1852_1853	618	S>fs	frameshift insertion
LPS22	*MUC4*	chr3	195512434	GTGTCG|-	6012_6017	2004_2006	>del	nonframeshift deletion
LPS22	*FAT3*	chr11	92533398	|C	7219_7220	2407	A>fs	frameshift insertion
LPS22	*MUC12*	chr7	100638976	A|T	5132	1711	E>V	missense
LPS22	*STAT5A*	chr17	40453398	|C	1095_1096	365	N>fs	frameshift insertion
LPS22	*SPHKAP*	chr2	229046289	A|G	26	9	V>A	missense
LPS23	*XIRP2*	chr2	168103916	G|T	5348	1783	C>F	missense
LPS23	*MUC4*	chr3	195506740	C|G	11711	3904	P>R	missense
LPS23	*CDH12*	chr5	22078635	C|A	151	51	R>S	missense
LPS23	*PLEC*	chr8	145001637	A|G	3709	1237	I>V	missense
LPS23	*PLIN2*	chr9	19126124	C|G	214	72	L>V	missense
LPS23	*OR8H2*	chr11	55872943	T|C	425	142	L>P	missense
LPS23	*ADAMTSL3*	chr15	84561467	G|C	1294	432	V>L	missense
LPS23	*OTOP3*	chr17	72942840	G|A	890	297	S>N	missense
LPS23	*ZNF135*	chr19	58579679	C|A	1863	621	H>Q	missense
LPS23	*CHEK2*	chr22	29090026	G|T	1455	485	W>C	missense
LPS23	*DLG4*	chr17	7096376	G|A	1745	582	S>F	missense
LPS23	*CHD6*	chr20	40162053	C|G	190	64	A>P	missense
LPS23	*CHD6*	chr20	40162055	G|A	188	63	T>I	missense
LPS23	*MAGEB6*	chrX	26212887	G|A	924	308	W>X	stopgain SNV
LPS23	*MGAM*	chr7	141752153	C|A	2865	955	S>R	missense
LPS23	*PLEC*	chr8	145001637	T|C	3655	1219	I>V	missense
LPS23	*FAT3*	chr11	92087262	G|A	1984	662	A>T	missense
LPS24	*PIK3CD*	chr1	9776589	C|T	692	231	P>L	missense
LPS24	*CSMD2*	chr1	34631386	C|A	29	10	P>H	missense
LPS24	*KDM5B*	chr1	202702626	C|T	3812	1271	S>L	missense
LPS24	*XIRP2*	chr2	168114435	C|T	1478	493	S>F	missense
LPS24	*SEMA3G*	chr3	52469941	C|A	2027	676	A>D	missense
LPS24	*MUC4*	chr3	195508211	C|A	10240	3414	P>T	missense
LPS24	*MUC4*	chr3	195510131	A|C	8320	2774	T>P	missense
LPS24	*MUC4*	chr3	195510146	C|G	8305	2769	L>V	missense
LPS24	*PLCB3*	chr11	64034960	G|A	3637	1213	A>T	missense
LPS24	*TELO2*	chr16	1545589	T|C	578	193	V>A	missense
LPS24	*DNAAF1*	chr16	84203737	G|A	1303	435	D>N	missense
LPS24	*ERBB2*	chr17	37871547	C|A	1067	356	A>D	missense
LPS24	*ABCC3*	chr17	48753065	G|A	2788	930	E>K	missense
LPS24	*ROS1*	chr6	117638307	G|T	6134	2045	T>K	missense
LPS24	*USP48*	chr1	22050420	C|A	1619	540	S>I	missense
LPS24	*PDE1C*	chr7	31867950	G|A	1241	414	P>L	missense
LPS24	*STXBP3*	chr1	109350042	G|T	555	519	A>S	missense
LPS24	*XIRP2*	chr2	168114435	C|T	713	238	S>F	missense
LPS24	*ERCC4*	chr16	14041971	G|A	2518	840	E>K	missense
LPS24	*TP53*	chr17	7576849	GTACCTGAAGG|-	591_597	197_199	del	frameshift

								deletion
LPS25	*SPHKAP*	chr2	228884010	A|T	1560	520	K>N	missense
LPS25	*MUC4*	chr3	195509771	G|A	8680	2894	A>T	missense
LPS25	*MUC4*	chr3	195512549	T|C	5920	1968	S>P	missense
LPS25	*FRG1*	chr4	190876263	A|T	389	130	D>V	missense
LPS25	*CSMD3*	chr8	113318376	T|A	7931	2644	L>Q	missense
LPS25	*ZHX2*	chr8	123964783	A|G	1033	345	I>V	missense
LPS25	*WDR96*	chr10	105912391	G|A	3634	1212	V>M	missense
LPS25	*EBF3*	chr10	131639180	G|A	1462	488	A>T	missense
LPS25	*FAT3*	chr11	92086345	G|A	1067	356	C>Y	missense
LPS25	*FAT3*	chr11	92577553	C|T	11020	3674	R>W	missense
LPS25	*ZNF135*	chr19	58579719	C|T	1903	635	Q>X	stopgain
LPS25	*PCDH15*	chr10	56423952	C|A	71	24	C>F	missense
LPS25	*ATM*	chr11	108100002	C|A	283	95	Q>K	missense
LPS26	*HEG1*	chr3	124738297	C|T	1397	466	S>F	missense
LPS26	*KCNMB3*	chr3	178957810	A|G	496	166	T>A	missense
LPS26	*SVIL*	chr10	29779914	G|A	2776	926	V>I	missense
LPS26	*BRCA1*	chr17	41226387	G|A	1324	442	D>N	missense
LPS26	*DNAH17*	chr17	76421626	T|G	12942	4314	F>L	missense
LPS27	*MUC4*	chr3	195510844	C|T	7607	2536	S>L	missense
LPS27	*MUC4*	chr3	195515123	A|G	3328	1110	T>A	missense
LPS27	*FRG1*	chr4	190876263	A|T	389	130	D>V	missense
LPS27	*MUC12*	chr7	100635229	T|G	1385	462	V>G	missense
LPS27	*CSMD3*	chr8	113299353	T|C	9271	3091	S>P	missense
LPS27	*MYO3A*	chr10	26462901	G|A	3708	1236	M>I	missense
LPS27	*PPP2R1B*	chr11	111625809	G|A	472	158	A>T	missense
LPS27	*TP53*	chr17	7579902	C|T	11	4	P>L	missense
LPS27	*NF1*	chr17	29556214	G|C	2581	861	A>P	missense
LPS27	*DNAH17*	chr17	76481728	G|A	7387	2463	V>M	missense
LPS27	*GLI1*	chr12	57865315	|G	2408_2409	803	L>	frameshift insertion
LPS27	*STAT5A*	chr17	40462642	|C	2340_2341	780	S>fs	frameshift insertion
LPS27	*EGF*	chr4	110884455	G|A		splicing	splicing	splicing
LPS27	*MUC12*	chr7	100635048	A|T	1204	402	S>C	missense
LPS27	*KIAA1033*	chr12	105521028	G|T	1160	387	R>M	missense
LPS27	*CDH1*	chr16	68863597	G|A	2336	779	R>Q	missense
LPS27	*RYR2*	chr1	237753227	A|G	3733	1245	R>G	missense
LPS27	*DNAH17*	chr17	76492074	G|A	5786	1929	A>V	missense
LPS28	*PROC*	chr2	128186155	C|T	1019	340	T>M	missense
LPS28	*ROBO1*	chr3	79639046	G|A	16	6	V>I	missense
LPS28	*MUC4*	chr3	195511804	C|T	6647	2216	S>L	missense
LPS28	*MUC4*	chr3	195512215	C|A	6236	2079	T>N	missense
LPS28	*MUC4*	chr3	195512501	C|T	5950	1984	P>S	missense
LPS28	*WDR66*	chr12	122399909	A|G	2333	778	Y>C	missense
LPS28	*CHEK2*	chr22	29083914	C|T	1603	535	R>C	missense
LPS28	*PARP1*	chr1	226579913	T|C	389	E130	E>G	missense
LPS28	*DAZAP2*	chr12	51636349	G|C	537	179	M>I	missense
LPS28	*DAZAP2*	chr12	51636350	T|A	538	180	L>M	missense
LPS28	*DAZAP2*	chr12	51636354	T|G	542	181	L>W	missense
LPS29	*VEPH1*	chr3	157081338	A|G	1550	517	D>G	missense
LPS29	*MUC4*	chr3	195506740	C|G	11711	3904	P>R	missense
LPS29	*MGAM*	chr7	141750579	C|T	2720	907	T>M	missense
LPS29	*WDR66*	chr12	122404930	G|A	2562	854	M>I	missense
LPS29	*DNAH17*	chr17	76482395	G|A	7012	2338	V>M	missense
LPS29	*PCDH15*	chr10	55581883	GTT|-	5481_5483	1827_1828	del	nonframeshift deletion
LPS29	*RCBTB1*	chr13	50141292	C|A	124	42	E>X	stopgain SNV
LPS30	*MUC4*	chr3	195506060	G|C	12391	4131	V>L	missense
LPS30	*MUC4*	chr3	195506101	A|T	12350	4117	D>V	missense
LPS30	*MUC4*	chr3	195506102	G|A	12349	4117	D>N	missense
LPS30	*MUC4*	chr3	195506303	C|A	12148	4050	P>T	missense
LPS30	*MUC4*	chr3	195506558	C|G	11893	3965	H>D	missense
LPS30	*MUC4*	chr3	195506581	A|G	11870	3957	D>G	missense
LPS30	*MUC4*	chr3	195507241	C|T	11210	3737	A>V	missense
LPS30	*MUC4*	chr3	195508490	G|T	9961	3321	A>S	missense

LPS30	*MUC4*	chr3	195508571	A|G	9880	3294	T>A	missense
LPS30	*MUC4*	chr3	195508574	G|C	9877	3293	D>H	missense
LPS30	*MUC4*	chr3	195510005	T|C	8446	2816	S>P	missense
LPS30	*MUC4*	chr3	195510073	C|T	8378	2793	A>V	missense
LPS30	*MUC4*	chr3	195510821	T|C	7630	2544	S>P	missense
LPS30	*MUC4*	chr3	195512480	T|C	5971	1991	S>P	missense
LPS30	*MUC4*	chr3	195513755	A|T	4696	1566	T>S	missense
LPS30	*MUC4*	chr3	195515383	C|A	3068	1023	T>N	missense
LPS30	*ZDHHC4*	chr7	6623017	T|C	451	151	C>R	missense
LPS30	*MUC12*	chr7	100637017	C|A	3173	1058	T>K	missense
LPS30	*PLEC*	chr8	144992660	G|A	11341	3781	E>K	missense
LPS30	*PLEC*	chr8	144998654	C|T	5455	1819	R>W	missense
LPS30	*MXRA5*	chrX	3239099	C|A	4627	1543	P>T	missense
LPS30	*NEUROD4*	chr12	55420785	G|A	562	188	D>N	missense
LPS30	*ZNF611*	chr19	53209226	A|G	1082	361	H>R	missense
LPS30	*TTI1*	chr20	36640810	G|A	1409	470	R>H	missense
LPS30	*CDH12*	chr5	22212753	C|T		splicing	splicing	splicing
LPS30	*DNAJC14*	chr12	56221695	C|T	748	250	A>T	missense
LPS30	*SACS*	chr13	23906349	T|A	11666	3889	Q>L	missense
LPS30	*BRCA2*	chr13	32929075	T|A	7085	2362	L>X	stopgain SNV
LPS30	*IQGAP1*	chr15	90997783	G|T	1611	537	E>D	missense
LPS30	*TTI1*	chr20	36640810	C|T	1409	470	R>H	missense
LPS30	*SLC5A3*	chr21	35468558	T|C	1061	354	L>P	missense
LPS30	*MUC4*	chr3	195507876	G|C	10575	3525	D>E	missense
LPS30	*MUC4*	chr3	195512455	G|T	5996	1999	T>N	missense
LPS30	*MUC4*	chr3	195508324	C|T	10127	3376	R>H	missense
LPS31	*CSMD2*	chr1	34011669	G|A	8683	2879	G>D	missense
LPS31	*FCAMR*	chr1	207134235	G|A	986	329	G>D	missense
LPS31	*RYR2*	chr1	237619939	C|T	1516	506	H>Y	missense
LPS31	*PIK3CA*	chr3	178952085	A|T	3140	1047	H>L	missense
LPS31	*MUC4*	chr3	195513605	C|T	4846	1616	P>S	missense
LPS31	*COG5*	chr7	107053002	G|A	707	236	R>Q	missense
LPS31	*PTEN*	chr10	89720852	C|T	1003	335	R>X	stopgain
LPS31	*SACS*	chr13	23905131	C|T	12884	4295	P>L	missense
LPS31	*DLG4*	chr17	7106253	T|G	885	295	S>R	missense
LPS31	*TP53*	chr17	7579362	T|G	325	109	F>V	missense
LPS31	*TP53*	chr17	7579705	G|A	91	31	V>I	missense
LPS31	*MYH3*	chr17	10533687	A|G	5375	1792	K>R	missense
LPS31	*DNAH17*	chr17	76497339	G|A	5410	1804	D>N	missense
LPS31	*STAT5A*	chr17	40459727	G|A	1892	631	W>X	stopgain SNV
LPS31	*MUC4*	chr3	195511325	A|C	7126	2376	S>A	missense
LPS31	*EGFR*	chr7	55273141	C|A	3464	1155	A>D	missense
LPS31	*DPP6*	chr7	153749970	C|T	65	22	P>L	missense
LPS31	*GLI1*	chr12	57864987	G|C	2080	694	V>L	missense
LPS31	*POLE*	chr12	133252700	C|T	1000	334	V>I	missense
LPS32	*MUC4*	chr3	195506362	C|G	12089	4030	A|G	missense
LPS32	*FRG1*	chr4	190873398	C|T	215	72	A|V	missense
LPS32	*FRG1*	chr4	190873412	C|A	229	77	L|I	missense
LPS32	*FRG1*	chr4	190876263	A|T	389	130	D|V	missense
LPS32	*ZNF75D*	chrX	134427902	C|G	165	55	F|L	missense
LPS32	*SACS*	chr13	23929778	G|A	973	325	G|R	missense
LPS32	*DST*	chr6	56481130	C|T	7135	2379	A>T	missense
LPS32	*MUC12*	chr7	100634583	G|A	739	247	E>K	missense
LPS32	*MUC4*	chr3	195509423	G|A	9028	3010	P>S	missense
LPS32	*MYO3A*	chr10	26312989	C|T	770	257	S>L	missense
LPS33	*MUC4*	chr3	195512724	C|G	5727	1909	C|G	missense
LPS33	*SEC63*	chr6	108279107	C|G	107	36	P|R	missense
LPS33	*ZHX2*	chr8	123965756	G|A	2006	669	R|Q	missense
LPS33	*PTPRD*	chr9	8486278	G|T	2539	847	G|T	missense
LPS33	*FAM47C*	chrX	37028425	A|G	1942	648	A|G	missense
LPS33	*TEP1*	chr14	20848429	G|T	4968	1656	G|T	missense
LPS33	*HSD17B2*	chr16	82124538	T|G	696	232	T|G	stopgain
LPS33	*DNAAF1*	chr16	84203675	G|T	1241	414	G|T	missense
LPS33	*MYH3*	chr17	10533717	G|A	5345	1782	G|A	missense
LPS33	*DNAH17*	chr17	76430258	T|C	12077	4026	T|C	missense
LPS33	*MTOR*	chr1	11204749	|G	4828_4829	1610	E>fs	frameshift

								insertion
LPS33	*SYT6*	chr1	114680173	C|T	1015	339	A>T	missense
LPS33	*WFS1*	chr4	6293079	C|A	616	206	Q>K	missense
LPS33	*MUC12*	chr7	100634272	G|A	428	143	S>N	missense
LPS33	*KIAA1033*	chr12	105557916	A|C	3185	1062	D>A	missense
LPS34	*MUC4*	chr3	195506014	C|T	12437	4146	P>L	missense
LPS34	*FRG1*	chr4	190883009	T|C	662	221	L>P	missense
LPS34	*MUC12*	chr7	100636285	C|T	2441	814	T>I	missense
LPS34	*FANCB*	chrX	14861817	A|G	2452	818	R>G	missense
LPS34	*PCDH15*	chr10	55582636	A|G	4643	1548	N>S	missense
LPS34	*STAT6*	chr12	57492585	C|A	2056	686	P>T	missense
LPS34	*PPFIA2*	chr12	81660770	T|A	3201	1067	N>K	missense
LPS34	*PRRT2*	chr16	29824442	E|K	67	23	E>K	missense
LPS34	*CYTIP*	chr2	158291218	C|A	216	72	W>C	missense
LPS34	*XIRP2*	chr2	168105193	A|G	6625	2209	K>E	missense
LPS34	*MUC12*	chr7	100645780	C|T	11936	3979	A>V	missense
LPS34	*DNAH17*	chr17	76481724	A|G	7391	2464	L>P	missense
LPS35	*ZDHHC4*	chr7	6623017	T|C	451	151	T>C	missense
LPS35	*SACS*	chr13	23909676	T|G	8339	2780	T>G	missense
LPS35	*CGNL1*	chr15	57816923	C|A	3031	1005	L>M	missense
LPS35	*MAPK7*	chr17	19285352	C|T	1736	579	A>V	missense
LPS35	*DNAH17*	chr17	76533473	G|A	2767	923	G>S	missense
LPS35	*PLCB4*	chr20	9374275	A|G	1364	455	K>R	missense
LPS35	*HDAC10*	chr22	50687098	T|C	899	300	V>A	missense
LPS35	*ZNF135*	chr19	58574825	C|T	208	70	P>S	missense
LPS35	*MXRA5*	chrX	3229597	G|A	6647	2216	A>V	missense
LPS35	*APC*	chr5	112177017	A|T	5672	1891	K>M	missense
LPS35	*NF1*	chr17	29663677	A|G	6109	2037	I>V	missense
LPS35	*SEMA3G*	chr3	52469744	G|A	2224	742	R>W	missense
LPS35	*PTEN*	chr10	89653835	G|-	133	45	V>fs	frameshift deletion
LPS35	*MUC12*	chr7	100643574	A|G	9730	3244	T>A	missense
LPS36	*USP48*	chr1	22033028	G|A	2143	715	E>K	missense
LPS36	*FRG1*	chr4	190873398	C|T	215	72	A>V	missense
LPS36	*FER1L6*	chr8	125103704	G|A	4432	1478	E>K	missense
LPS36	*ZNF135*	chr19	58578555	A|C	739	247	T>P	missense
LPS36	*MDC1*	chr6	30672734	G|A	4226	1409	P>L	missense
LPS36	*MGAM*	chr7	141750046	A|G	2599	867	N>D	missense
LPS37	*ADAMTS4*	chr1	161168120	T|C	298	100	S>P	missense
LPS37	*KDM5B*	chr1	202710733	G|C	2707	903	E>Q	missense
LPS37	*MUC4*	chr3	195513601	T|G	4850	1617	L>R	missense
LPS37	*FRG1*	chr4	190873370	G|A	187	63	E>K	missense
LPS37	*FRG1*	chr4	190883009	T|C	662	221	L>P	missense
LPS37	*MUC12*	chr7	100637253	G|A	3409	1137	G>S	missense
LPS37	*KIAA1033*	chr12	105527595	T|C	1247	416	M>T	missense
LPS37	*CGNL1*	chr15	57816882	G|A	2972	991	R>K	missense
LPS37	*LLGL1*	chr17	18145527	C|T	2930	977	P>L	missense
LPS37	*ERBB2*	chr17	37871547	C|A	1067	356	A>D	missense
LPS37	*DNAH17*	chr17	76446894	T|C	10769	13590	I>T	missense
LPS37	*DNAH17*	chr17	76510927	C|T	4042	1348	R>C	missense
LPS37	*DNAH17*	chr17	76548866	G|A	2200	7341	V>I	missense
LPS37	*LIPE*	chr19	42912229	G|A	1555	519	D>N	missense
LPS37	*CHEK2*	chr22	29083914	C|T	1603	535	R>C	missense
LPS37	*MUC4*	chr3	195507796	G|A	10655	3552	P>L	missense
LPS37	*MDC1*	chr6	30679999	G|A	1720	574	H>Y	missense
LPS37	*MUC4*	chr3	195509998	G|A	8453	2818	P>L	missense
LPS37	*MUC12*	chr7	100651918	C|T	15062	5021	T>I	missense
LPS37	*DOCK4*	chr7	111555870	T|C	1156	386	T>A	missense
LPS37	*RPUSD2*	chr15	40866327	G|A	1505	502	C>Y	missense
LPS37	*TP53BP1*	chr15	43748588	C|T	2218	740	D>N	missense
LPS37	*CHD6*	chr20	40033369	G|A	8012	2671	P>L	missense
LPS37	*ZNF75D*	chrX	134424999	A|C	474	158	N>K	missense
LPS37	*MUC4*	chr3	195508228	G|C	10223	3408	P>R	missense
LPS37	*NF1*	chr17	29553477	|C	2026_2027	676	T>fs	frameshift insertion
LPS38	*DNAH14*	chr1	225268106	C|A	2792	931	A>D	missense

LPS38	*SEMA3G*	chr3	52469941	C|A	2027	676	A>D	missense
LPS38	*MUC4*	chr3	195510100	C|G	8351	2784	P>R	missense
LPS38	*ABCC3*	chr17	48742513					missense
LPS39	*USP34*	chr2	61441439	C|G	8438	2813	P>R	missense
LPS39	*MUC4*	chr3	195511804	C|T	6647	2216	S>L	missense
LPS39	*MUC4*	chr3	195512215	C|A	6236	2079	T>N	missense
LPS39	*FRG1*	chr4	190876263	A|T	389	130	D>V	missense
LPS39	*MUC12*	chr7	100634700	G|C	856	286	D>H	missense
LPS39	*CSMD3*	chr8	113662518	A|G	3065	1022	Y>C	missense
LPS39	*PLEC*	chr8	145049380	T|C	158	53	L>P	missense
LPS39	*EGFR*	chr7	55238022	G|T	1903	635	A>S	missense
LPS39	*MIA2*	chr14	39709847	C|T	332	111	T>M	missense
LPS39	*COG1*	chr17	71193106	G|C	628	210	E>Q	missense
LPS39	*ZNF135*	chr19	58578735	G|T	919	307	E>X	stopgain SNV
LPS39	*SEZ6L*	chr22	26688743	CTC|-	466_468	156_156	del	nonframeshift deletion
LPS40	*ERBB4*	chr2	212652807	G|A	499	1671	V>I	missense
LPS40	*MUC4*	chr3	195511076	A|C	7375	2459	T>P	missense
LPS40	*MUC4*	chr3	195516350	G|A	2101	701	G>R	missense
LPS40	*MUC12*	chr7	100644430	C|T	10586	3529	C>T	missense
LPS40	*MGAM*	chr7	141795404	A|G	4810	1604	I>V	missense
LPS40	*CSMD3*	chr8	113299353	T|C	9271	3091	S>P	missense
LPS40	*FER1L6*	chr8	125083826	C|T	4046	1349	P>L	missense
LPS40	*UBQLNL*	chr11	5536386	T|C	1286	429	M>T	missense
LPS40	*OR5M3*	chr11	56237441	C|T	533	178	A>V	missense
LPS40	*OR5M3*	chr11	56237454	C|A	520	174	H>N	missense
LPS40	*OR5M3*	chr11	56237805	A|G	169	57	M>V	missense
LPS40	*TELO2*	chr16	1551750	C|T	1448	483	A>V	missense
LPS40	*DNAH17*	chr17	76420015	C|G	13361	4454	A>G	missense
LPS40	*DNAH17*	chr17	76565322	G|A	1210	404	V>M	missense
LPS40	*LIPE*	chr19	42914964	G|A	914	305	R>H	missense
LPS40	*WFS1*	chr4	6296874	G|T	819	273	E>D	missense
LPS40	*XIRP2*	chr2	168104762	|C	6194_6195	2065	C>fs	frameshift insertion
LPS41	*ZMYM4*	chr1	35846970	C|T	1292	431	P>L	missense
LPS41	*MUC4*	chr3	195508067	A|C	10384	3462	T>P	missense
LPS41	*MUC4*	chr3	195509401	T|C	9050	3017	I>T	missense
LPS41	*MUC4*	chr3	195511012	C|A	7439	2480	P>H	missense
LPS41	*PLEC*	chr8	145004388	G|A	2548	850	G>S	missense
LPS41	*SVIL*	chr10	29779914	G|A	2776	926	V>I	missense
LPS41	*OR8H2*	chr11	55872943	T|C	425	142	L>P	missense
LPS41	*NEUROD4*	chr12	55420891	A|G	668	223	H>R	missense
LPS41	*MYH3*	chr17	10541497	G|A	3592	1198	A>T	missense
LPS41	*KRTAP10-11*	chr21	46066833	A|T	458	153	K>M	missense
LPS41	*PCDH15*	chr10	55721636	C|G	2672	891	R>P	missense
LPS42	*SPHKAP*	chr2	228883123	G|A	2447	816	R>H	missense
LPS42	*ROBO1*	chr3	78688908	C|G	2888	963	C>G	missense
LPS42	*PIK3CA*	chr3	178952085	A|G	3140	1047	H>R	missense
LPS42	*DPP6*	chr7	154564589	T|C	1073	358	V>A	missense
LPS42	*MXRA5*	chrX	3240238	G|A	3488	1163	R>H	missense
LPS42	*BTBD7*	chr14	93760935	T|C	431	144	V>A	missense
LPS42	*BTBD7*	chr14	93760936	G|A	430	144	V>I	missense
LPS42	*NF1*	chr17	29553477	|C	2026_2027	676	T>fs	frameshift insertion
LPS43	*KDM5B*	chr1	202700156	G|A	4057	1353	A>T	missense
LPS43	*DNAH14*	chr1	225268106	C|A	2792	931	A>D	missense
LPS43	*MGAM*	chr7	141803124	T|C	5381	1794	L>S	missense
LPS43	*PLEC*	chr8	144996830	G|A	7279	2427	A>T	missense
LPS43	*FAT3*	chr11	92086828	T|C	1550	517	L>S	missense
LPS43	*KIAA1033*	chr12	105538204	G|A	2150	717	R>Q	missense
LPS43	*CHEK2*	chr22	29083914	C|T	1603	535	R>C	missense
LPS43	*DNAH17*	chr17	76480963	C|A	7636	2546	D>Y	missense
LPS44	*PRKD3*	chr2	37513364	A|G	866	289	K>R	missense
LPS44	*PIKFYVE*	chr2	209200079	T|C	4192	1398	F>L	missense
LPS44	*MUC4*	chr3	195486102	G|A	2018	673	R>H	missense
LPS44	*MUC4*	chr3	195510005	T|C	8446	2816	S>P	missense

LPS44	*MUC4*	chr3	195510011	G|A	8440	2814	A>T	missense
LPS44	*MUC4*	chr3	195513532	C|T	4919	1640	S>L	missense
LPS44	*MUC4*	chr3	195517076	G|A	1375	459	A>T	missense
LPS44	*MUC4*	chr3	195518120	A|C	331	111	M>L	missense
LPS44	*APC*	chr5	112043485	C|T	71	24	S>F	missense
LPS44	*PLEC*	chr8	144994514	G|A	9487	3163	A>T	missense
LPS44	*MXRA5*	chrX	3239362	C|T	4364	1455	A>V	missense
LPS44	*MXRA5*	chrX	3240106	A|T	3620	1207	D>V	missense
LPS44	*MXRA5*	chrX	3240107	G|A	3619	1207	D>N	missense
LPS44	*MXRA5*	chrX	3240140	G|A	3586	1196	V>M	missense
LPS44	*MXRA5*	chrX	3240145	G|A	3581	1194	S>N	missense
LPS44	*MXRA5*	chrX	3240238	G|A	3488	1163	R>H	missense
LPS44	*MXRA5*	chrX	3240253	G|A	3473	1158	R>H	missense
LPS44	*MXRA5*	chrX	3241032	A|G	2694	898	I>M	missense
LPS44	*OR8H2*	chr11	55872943	T|C	425	142	L>P	missense
LPS44	*FAT3*	chr11	92570856	G|T	10252	3418	A>S	missense
LPS44	*TELO2*	chr16	1545589	T|C	578	193	V>A	missense
LPS44	*TP53*	chr17	7578208	A|G	245	82	H>R	missense
LPS44	*OTOP3*	chr17	72937649	C|T	235	79	R>W	missense
LPS44	*MUC4*	chr3	195511957	T|A	6494	2165	D>V	missense
LPS44	*BTBD7*	chr14	93760476	C|T	890	297	R>Q	missense
LPS44	*MUC4*	chr3	195508700	C|T	9751	3251	V>I	missense
LPS44	*MUC4*	chr3	195507876	G|C	10575	3525	D>E	missense
LPS44	*NF1*	chr17	29553477	|C	2026_2027	676	T>fs	frameshift insertion
LPS45	*MUC4*	chr3	195508544	T|A	9907	3303	S>T	missense
LPS45	*MUC4*	chr3	195510146	C|G	8305	2769	L>V	missense
LPS45	*TRAPPC11*	chr4	184585165	G|C	145	49	V>L	missense
LPS45	*APC*	chr5	112178027	G|A	6682	2228	V>I	missense
LPS45	*FER1L6*	chr8	125047645	C|T	2414	805	C>T	missense
LPS45	*OR8H2*	chr11	55872943	T|C	425	142	T>C	missense
LPS45	*TEP1*	chr14	20874411	A|G	716	239	H>R	missense
LPS45	*TTI1*	chr20	36640975	T|C	1244	415	I>T	missense
LPS46	*XIRP2*	chr2	168074745	G|A	127	43	E>K	missense
LPS46	*XIRP2*	chr2	168103244	T|C	4674	1559	I>T	missense
LPS46	*PIK3CA*	chr3	178938787	A|G	2029	677	N>D	missense
LPS46	*MUC4*	chr3	195506399	C|A	12052	4018	P>T	missense
LPS46	*MUC4*	chr3	195509345	G|A	9106	3036	G>S	missense
LPS46	*MUC4*	chr3	195511156	G|C	7295	2432	R>P	missense
LPS46	*MUC4*	chr3	195511186	T|C	7265	2422	L>P	missense
LPS46	*MUC4*	chr3	195512219	G|A	6232	2078	A>T	missense
LPS46	*MUC4*	chr3	195512339	A|G	6112	2038	T>A	missense
LPS46	*MUC4*	chr3	195512734	A|C	5717	1906	H>P	missense
LPS46	*MUC4*	chr3	195512750	G|C	5701	1901	D>H	missense
LPS46	*MUC4*	chr3	195515198	G|C	3253	1085	D>H	missense
LPS46	*MUC4*	chr3	195515209	C|T	3242	1081	A>V	missense
LPS46	*MUC4*	chr3	195515257	C|A	3194	1065	A>E	missense
LPS46	*WFS1*	chr4	6303017	C|T	1495	499	L>F	missense
LPS46	*WFS1*	chr4	6303857	G|A	2335	779	V>M	missense
LPS46	*DST*	chr6	56418399	A|G	7322	2441	Q>R	missense
LPS46	*MUC12*	chr7	100635844	C|T	2000	667	S>L	missense
LPS46	*MUC12*	chr7	100658913	C|T	15818	5273	P>L	missense
LPS46	*MGAM*	chr7	141738362	C|T	2263	755	H>Y	missense
LPS46	*PLEC*	chr8	145013572	A|G	58	20	S>G	missense
LPS46	*PTPRD*	chr9	8460508	A|G	3778	1260	I>V	missense
LPS46	*MXRA5*	chrX	3240445	C|T	3281	1094	T>I	missense
LPS46	*DRGX*	chr10	50574234	C|T	734	245	P>L	missense
LPS46	*OR5M3*	chr11	56237441	C|T	533	178	A>V	missense
LPS46	*OR5M3*	chr11	56237454	C|A	520	174	H>N	missense
LPS46	*OR5M3*	chr11	56237805	A|G	169	57	M>V	missense
LPS46	*HEPHL1*	chr11	93796782	C|T	524	175	T>I	missense
LPS46	*ATM*	chr11	108138010	A|G	2579	860	D>G	missense
LPS46	*TEP1*	chr14	20873695	T|C	785	262	M>T	missense
LPS46	*TP53BP1*	chr15	43749149	A|G	1657	553	T>A	missense
LPS46	*FANCA*	chr16	89811469	C|T	3524	1175	P>L	missense
LPS46	*MYH3*	chr17	10534960	G|A	5254	1752	A>T	missense

LPS46	*DNAH17*	chr17	76472713	G|A	8095	2699	E>K	missense
LPS46	*DNAH17*	chr17	76501475	G|A	4856	1619	R>Q	missense
LPS46	*DNAH17*	chr17	76502928	C|A	4677	1559	S>R	missense
LPS46	*ZNF611*	chr19	53209906	G|C	402	134	K>N	missense
LPS46	*ZNF611*	chr19	53219129	G|C	19	7	A>P	missense
LPS46	*ZNF135*	chr19	58578703	C|T	887	296	P>L	missense
LPS46	*TTI1*	chr20	36640619	G|T	1600	534	A>S	missense
LPS46	*KRTAP10-11*	chr21	46066823	G|A	448	150	V>I	missense
LPS47	*ZMYM4*	chr1	35853140	A|G	2198	733	K>R	missense
LPS47	*CYTIP*	chr2	158272436	C|T	833	278	T>M	missense
LPS47	*FANCD2*	chr3	10128928	C|T	3446	1149	A>V	missense
LPS47	*MUC4*	chr3	195509383	C|A	9068	3023	T>N	missense
LPS47	*MUC4*	chr3	195510770	C|G	7681	2561	L>V	missense
LPS47	*MUC4*	chr3	195511258	C|T	7193	2398	T>I	missense
LPS47	*MUC4*	chr3	195511879	C|T	6572	2191	T>I	missense
LPS47	*MUC4*	chr3	195515263	C|T	3188	1063	S>F	missense
LPS47	*DST*	chr6	56494193	G|A	2719	907	E>K	missense
LPS47	*MUC12*	chr7	100636543	C|G	2699	900	T>S	missense
LPS47	*MGAM*	chr7	141763330	G|A	4289	1430	G>E	missense
LPS47	*MGAM*	chr7	141803214	C|T	5471	1824	S>F	missense
LPS47	*PLEC*	chr8	145001159	A|G	3943	1315	S>G	missense
LPS47	*HEPHL1*	chr11	93803554	C|A	1078	360	Q>K	missense
LPS47	*TEP1*	chr14	20837573	C|T	7586	2529	A>V	missense
LPS47	*XRCC1*	chr19	44050759	G|T	1414	472	G>W	missense
LPS48	*DNAH14*	chr1	225490865	A|G	8360	2787	Y>C	missense
LPS48	*MUC4*	chr3	195509401	T|C	9050	3017	A>T	missense
LPS48	*MUC4*	chr3	195516350	G|A	2101	701	G>R	missense
LPS48	*MUC12*	chr7	100647091	C|T	13247	4416	P>L	missense
LPS48	*DPP6*	chr7	153749952	G|C	47	16	R>T	missense
LPS48	*MXRA5*	chrX	3240238	G|A	3488	1163	R>H	missense
LPS48	*MXRA5*	chrX	3240253	G|A	3473	1158	R>H	missense
LPS48	*MXRA5*	chrX	3261745	C|T	130	44	R>X	missense
LPS48	*ZNF280C*	chrX	129370463	C|T	644	215	S>F	missense
LPS48	*SLITRK2*	chrX	144904813	T|G	870	290	N>K	missense
LPS48	*FAT3*	chr11	92624267	G|T	13662	4554	Q>H	missense
LPS48	*KIAA1033*	chr12	105512284	G|A	496	166	A>T	missense
LPS48	*XRCC1*	chr19	44055777	G|A	1145	382	R>H	missense
LPS49	*PUM1*	chr1	31465377	A|C	1018	340	M>L	missense
LPS49	*DNAH14*	chr1	225268106	C|A	2792	931	A>D	missense
LPS49	*ZBTB38*	chr3	141162449	C|T	1219	407	R>C	missense
LPS49	*MUC4*	chr3	195506233	C|T	12218	4073	A>V	missense
LPS49	*MUC4*	chr3	195512099	C|A	6352	2118	A>T	missense
LPS49	*WFS1*	chr4	6292945	G|A	482	161	R>Q	missense
LPS49	*PDE1C*	chr7	31855577	T|G	1774	592	S>A	missense
LPS49	*MGAM*	chr7	141732634	G|A	1594	532	E>K	missense
LPS49	*PLEC*	chr8	145002053	C|T	3380	1127	P>L	missense
LPS49	*ATM*	chr11	108160480	T|G	4388	1463	F>C	missense
LPS49	*NDUFA9*	chr12	4771733	C|T	587	196	P>L	missense
LPS49	*DAZAP2*	chr12	51636178	T|C	377	126	L>P	missense
LPS49	*SACS*	chr13	23929378	C|T	1373	458	T>I	missense
LPS49	*DNAAF1*	chr16	84203737	G|A	1303	435	D>N	missense
LPS49	*DNAH17*	chr17	76565533	C|A	1121	374	S>Y	missense
LPS50	*CSMD2*	chr1	34071533	G|C	6279	2093	E>D	missense
LPS50	*DNAH14*	chr1	225586807	G|A	13384	4462	A>T	missense
LPS50	*MUC4*	chr3	195507316	C|T	11135	3712	P>L	missense
LPS50	*MUC4*	chr3	195510976	G|T	7475	2492	G>V	missense
LPS50	*MUC4*	chr3	195511081	C|A	7370	2457	A>E	missense
LPS50	*DST*	chr6	56457044	A|G	4970	1657	K>R	missense
LPS50	*PDE1C*	chr7	31855577	T|G	1774	592	S>A	missense
LPS51	*XIRP2*	chr2	168099316	T|C	748	250	S>P	missense
LPS51	*XIRP2*	chr2	168101537	C|T	2969	990	I>T	missense
LPS51	*ROBO1*	chr3	79067604	C|T	8	3	A>V	missense
LPS51	*MUC4*	chr3	195506060	G|C	12391	4131	V>L	missense
LPS51	*MUC4*	chr3	195506303	C|A	12148	4050	P>T	missense
LPS51	*MUC4*	chr3	195509077	A|G	9374	3125	D>G	missense

LPS51	*MUC4*	chr3	195509078	G|A	9373	3125	D>N	missense
LPS51	*MUC4*	chr3	195512480	T|C	5971	1991	S>P	missense
LPS51	*MUC4*	chr3	195516350	G|A	2101	701	G>R	missense
LPS51	*KIT*	chr4	55595614	C|G	2092	698	L>V	missense
LPS51	*FRG1*	chr4	190876263	A|T	389	130	D>V	missense
LPS51	*MDC1*	chr6	30671807	C|T	5153	1718	P>L	missense
LPS51	*MDC1*	chr6	30671959	C|G	5001	1667	D>E	missense
LPS51	*MDC1*	chr6	30672028	G|T	4932	1644	R>S	missense
LPS51	*MDC1*	chr6	30680442	T|C	1277	426	I>T	missense
LPS51	*MAGEB6*	chrX	26212477	G|C	514	172	G>R	missense
LPS51	*NEUROD4*	chr12	55420785	G|A	562	188	D>N	missense
LPS51	*MAPK6*	chr15	52357148	C|T	2117	706	P>L	missense
LPS51	*FANCA*	chr16	89825107	C|G	2859	953	D>E	missense
LPS52	*SPHKAP*	chr2	228881332	C|T	4238	1413	P>L	missense
LPS52	*MUC4*	chr3	195507521	G|A	10930	3644	G>S	missense
LPS52	*MUC4*	chr3	195507876	C|G	10575	3525	D>E	missense
LPS52	*MUC4*	chr3	195508094	C|G	10357	3453	H>D	missense
LPS52	*MUC4*	chr3	195508117	G|A	10334	3445	S>N	missense
LPS52	*MUC4*	chr3	195508118	A|G	10333	3445	S>G	missense
LPS52	*MUC4*	chr3	195508418	G|C	10033	3345	V>L	missense
LPS52	*MUC4*	chr3	195508453	G|A	9998	3333	S>N	missense
LPS52	*MUC4*	chr3	195509249	G|A	9202	3068	G>S	missense
LPS52	*MUC4*	chr3	195510005	T|C	8446	2816	S>P	missense
LPS52	*MUC4*	chr3	195510073	C|T	8378	2793	A>V	missense
LPS52	*MUC4*	chr3	195510085	G|A	8366	2789	S>N	missense
LPS52	*MUC4*	chr3	195511404	C|G	7047	2349	H>Q	missense
LPS52	*MUC4*	chr3	195512117	G|T	6334	2112	A>S	missense
LPS52	*MUC4*	chr3	195515257	C|A	3194	1065	A>E	missense
LPS52	*MUC4*	chr3	195515383	C|A	3068	1023	T>N	missense
LPS52	*TRAPPC11*	chr4	184587424	G|C	219	73	E>D	missense
LPS52	*DST*	chr6	56400047	G|C	8945	2982	C>S	missense
LPS52	*MUC12*	chr7	100660897	T|C	15919	5307	Y>H	missense
LPS52	*DOCK4*	chr7	111379198	C|G	5197	1733	P>A	missense
LPS52	*MXRA5*	chrX	3240650	C|T	3076	1026	P>S	missense
LPS52	*MXRA5*	chrX	3241739	A|G	1987	663	T>A	missense
LPS52	*FAT3*	chr11	92088447	C|T	3169	1057	R>C	missense
LPS52	*HEPHL1*	chr11	93803598	G|C	1122	374	K>N	missense
LPS53	*ITSN2*	chr2	24432835	A|C	4325	1442	Y>S	missense
LPS53	*SPHKAP*	chr2	228883499	G|A	2071	691	D>N	missense
LPS53	*MUC4*	chr3	195508453	G|A	9998	3333	S>N	missense
LPS53	*MUC4*	chr3	195510155	G|A	8296	2766	A>T	missense
LPS53	*ETV7*	chr6	36334749	C|T	580	194	R>W	missense
LPS53	*DOPEY1*	chr6	83806760	G|A	64	22	D>N	missense
LPS53	*SVIL*	chr10	29839785	G|T	568	190	G>C	missense
LPS53	*FAT3*	chr11	92532626	G|T	6447	2149	L>F	missense
LPS53	*ERBB3*	chr12	56493492	G|A	2900	967	R>K	missense
LPS53	*BRCA2*	chr13	32907183	A|G	1568	523	H>R	missense
LPS53	*TELO2*	chr16	1547104	G|C	681	227	Q>H	missense
LPS53	*FANCA*	chr16	89851304	C|G	1428	476	F>L	missense
LPS53	*DNAH17*	chr17	76481692	G|A	7423	2475	D>N	missense
LPS53	*SEZ6L*	chr22	26688840	G|A	563	188	R>Q	missense
LPS54	*ZMYM4*	chr1	35870648	G|A	3553	1185	V>M	missense
LPS54	*SPHKAP*	chr2	228886543	C|T	581	194	T>I	missense
LPS54	*SPHKAP*	chr2	228886544	A|T	580	194	T>S	missense
LPS54	*ADH6*	chr4	100130064	G|A	589	197	A>T	missense
LPS54	*MDC1*	chr6	30675807	A|G	2549	850	E>G	missense
LPS54	*FER1L6*	chr8	125081694	T|C	3812	1271	I>T	missense
LPS54	*STOX1*	chr10	70645782	G|A	2230	744	E>K	missense
LPS54	*MIA2*	chr14	39716698	C|A	920	307	T>N	missense
LPS54	*ZNF611*	chr19	53208581	G|A	1727	576	S>N	missense
LPS54	*ZNF611*	chr19	53209990	T|G	318	106	I>M	missense
LPS54	*ZNF611*	chr19	53210025	A|C	283	95	S>R	missense
LPS57	*FRG1*	chr4	190883009	T|C	662	221	L>P	missense
LPS57	*MUC12*	chr7	100634892	G|A	1048	350	A>T	missense
LPS57	*BTBD7*	chr14	93712301	C|T	2453	818	P>L	missense
LPS57	*BRCA1*	chr17	41231356	T|C	4418	1473	I>T	missense

LPS57	*KRTAP10-11*	chr21	46066872	C|A	497	166	S>Y	missense
LPS57	*SEMA3G*	chr3	52475656	T|C	601	201	M>V	missense
LPS57	*CACNA1D*	chr3	53839069	G|A	5573	1858	R>Q	missense
LPS57	*ROS1*	chr6	117678977	G|A	3844	1282	P>S	missense
LPS57	*SVIL*	chr10	29773618	A|T	3644	1215	L>Q	missense
LPS58	*FRG1*	chr4	190873412	C|A	229	77	L>I	missense
LPS58	*FRG1*	chr4	190876263	A|T	389	130	D>V	missense
LPS58	*DST*	chr6	56394455	G|A	9517	3173	D>N	missense
LPS58	*THSD7A*	chr7	11676433	G|A	346	116	D>N	missense
LPS58	*MGAM*	chr7	141754571	G|C	3177	1059	K>N	missense
LPS58	*FER1L6*	chr8	125022902	A|G	1769	590	Q>R	missense
LPS58	*PCDH15*	chr10	55582674	G|T	4605	1535	R>S	missense
LPS58	*MIA2*	chr14	39716293	C|A	515	172	T>N	missense
LPS58	*LIPE*	chr19	42911903	G|A	1697	566	R>H	missense
LPS58	*ZNF611*	chr19	53210025	A|C	283	95	S>R	missense
LPS58	*ZNF135*	chr19	58579693	C|T	1913	638	S>F	missense
LPS58	*RYR2*	chr1	237713869	G|T	3092	1031	R>L	missense
LPS58	*SEC63*	chr6	108279193	C|G	21	7	Q>H	missense
LPS59	*FAT3*	chr11	92087559	G|A	2281	761	V>M	missense
LPS59	*SPOCK3*	chr4	168155231	|C	94_95	32	R>fs	frameshift insertion
LPS59	*PLBD1*	chr12	14664548	C|A	942	314	Q>H	missense
LPS59	*MDC1*	chr6	30670561	G|T	5959	1987	L>M	missense
LPS59	*PLBD1*	chr12	14656807	|A	1561_1562	521	R>fs	frameshift insertion
LPS59	*ATM*	chr11	108192089	A|C	6514	2172	T>P	missense
LPS59	*SPHKAP*	chr2	228884717	G|A	853	285	R>X	stopgain SNV
LPS59	*TTI1*	chr20	36641168	A|G	1051	351	C>R	missense
LPS59	*APC*	chr5	112177434	|A	6089_6090	2030	P>fs	frameshift insertion
LPS59	*STAT1*	chr2	191849061	T|A	1322	441	Q>L	missense
LPS59	*MCM6*	chr2	136622724	|C	937_938	313	K>fs	frameshift insertion
LPS59	*ADAMTS4*	chr1	161167945	|C	473_474	158	A>fs	frameshift insertion
LPS61	*SYT6*	chr1	114680207	G|A	726	242	M>I	missense
LPS61	*FRG1*	chr4	190873398	C|T	215	72	A>V	missense
LPS61	*FRG1*	chr4	190873412	C|A	229	77	L>I	missense
LPS61	*AGAP2*	chr12	58126703	G|A	601	201	A>T	missense
LPS61	*PPFIA2*	chr12	81769660	G|A	749	250	S>N	missense
LPS61	*POLE*	chr12	133218264	G|A	5347	1783	D>N	missense
LPS61	*CHEK2*	chr22	29090043	G|A	1438	480	A>T	missense
LPS62	*STXBP3*	chr1	109315383	G|A	535	179	D>N	missense
LPS62	*PARP1*	chr1	226568779	C|G	1290	430	I>M	missense
LPS62	*ZBTB38*	chr3	141161943	G|A	713	238	R>H	missense
LPS62	*MUC4*	chr3	195508228	C|G	10223	3408	P>R	missense
LPS62	*MUC4*	chr3	195509354	G|T	9097	3033	A>S	missense
LPS62	*MUC4*	chr3	195513601	T|G	4850	1617	L>R	missense
LPS62	*FRG1*	chr4	190873398	C|T	215	72	A>V	missense
LPS62	*DOPEY1*	chr6	83841940	C|T	2662	888	Q>X	stopgain
LPS62	*MUC12*	chr7	100648363	G|A	14519	4840	S>N	missense
LPS62	*LPL*	chr8	19818567	G|C	1295	432	R>T	missense
LPS62	*TTI2*	chr8	33360956	T|G	1250	417	L>R	missense
LPS62	*HEPHL1*	chr11	93779033	G|A	365	122	R>Q	missense
LPS62	*DNAJC14*	chr12	56221791	C|T	652	218	P>S	missense
LPS62	*LLGL1*	chr17	18144825	T|C	2563	855	C>R	missense
LPS62	*ABCC3*	chr17	48753897	C|T	3326	1109	P>L	missense
LPS62	*ABCC3*	chr17	48755222	C|G	3496	1166	R>G	missense
LPS62	*ZNF611*	chr19	53208943	A|T	1365	455	K>N	missense
LPS62	*ZNF611*	chr19	53209226	A|G	1082	361	H>R	missense
LPS62	*ZNF611*	chr19	53209365	T|C	943	315	Y>H	missense
LPS62	*ZNF611*	chr19	53209368	C|T	940	314	R>C	missense
LPS63	*DST*	chr6	56481427	C|T	6838	2280	Q>X	stopgain
LPS63	*MXRA5*	chrX	3241318	C|G	2408	803	P>R	missense
LPS63	*MYO3A*	chr10	26359125	T|C	1256	419	I>T	missense
LPS63	*WDR66*	chr12	122399945	C|T	2369	790	T>I	missense

LPS63	*POLE*	chr12	133209337	C|T	6049	2017	R>C	missense
LPS63	*NF1*	chr17	29552200	A|G	1933	645	M>V	missense
LPS63	*ABCC3*	chr17	48750483	G|T	2393	798	G>V	missense
LPS63	*DNAH17*	chr17	76522985	C|T	3601	1201	R>W	missense
LPS64	*ZMYM4*	chr1	35824720	G|A	280	94	D>N	missense
LPS64	*ADAMTS4*	chr1	161161275	C|T	2167	723	R>W	missense
LPS64	*RYR2*	chr1	237972294	G|A	14392	4798	D>N	missense
LPS64	*PIKFYVE*	chr2	209190719	T|C	3184	1062	F>L	missense
LPS64	*SPHKAP*	chr2	228884362	C|A	1208	403	A>E	missense
LPS64	*MUC4*	chr3	195507205	G|A	11246	3749	S>N	missense
LPS64	*MUC4*	chr3	195507271	C|G	11180	3727	T>S	missense
LPS64	*MUC4*	chr3	195508415	C|A	10036	3346	P>T	missense
LPS64	*MUC4*	chr3	195516451	C|T	2000	667	P>L	missense
LPS64	*DST*	chr6	56483760	C|T	5072	1691	S>F	missense
LPS64	*BRAF*	chr7	140439659	G|A	2080	694	A>T	missense
LPS64	*PLEC*	chr8	144991205	G|A	12796	4266	V>I	missense
LPS64	*PCDH15*	chr10	55663053	G|A	3238	1080	G>R	missense
LPS64	*TELO2*	chr16	1557677	C|G	2367	789	D>E	missense
LPS64	*NF1*	chr17	29553610	G|A	2159	720	R>Q	missense
LPS64	*ERBB2*	chr17	37866641	G|T	718	240	A>S	missense
LPS64	*ABCC3*	chr17	48753036	C|T	2759	920	C>T	missense
LPS64	*DNAH17*	chr17	76446386	A|G	10990	3664	I>V	missense
LPS64	*DNAH17*	chr17	76487632	C|T	6562	2188	R>X	stopgain
LPS64	*ZNF611*	chr19	53209909	A|T	399	133	K>N	missense
LPS65	*USP34*	chr2	61433170	C|G	9136	3046	L>V	missense
LPS65	*MUC4*	chr3	195510757	A|G	7694	2565	D>G	missense
LPS65	*MUC4*	chr3	195510758	G|A	7693	2565	D>N	missense
LPS65	*MUC4*	chr3	195513068	G|T	5383	1795	V>F	missense
LPS65	*EGF*	chr4	110885619	A|G	1501	501	T>A	missense
LPS65	*DOPEY1*	chr6	83841983	A|C	2705	902	N>T	missense
LPS65	*MUC12*	chr7	100635289	G|A	1445	482	G>D	missense
LPS65	*MUC12*	chr7	100644430	C|T	10586	3529	T>M	missense
LPS65	*OR2A14*	chr7	143826464	C|A	259	87	Q>K	missense
LPS65	*RNF32*	chr7	156447325	G|C	330	110	W>C	missense
LPS65	*FER1L6*	chr8	125072864	T|C	3061	1021	C>R	missense
LPS65	*DRGX*	chr10	50574204	C|T	764	255	T>I	missense
LPS65	*BRCA2*	chr13	32971071	C|T	9538	3180	L>F	missense
LPS65	*TP53BP1*	chr15	43749236	A|G	1570	524	T>A	missense
LPS65	*CGNL1*	chr15	57731245	A|G	1048	350	I>V	missense
LPS65	*STAT5A*	chr17	40451868	G|A	650	217	R>H	missense
LPS65	*DNAH17*	chr17	76455237	C|T	9707	3236	T>M	missense
LPS65	*ZSCAN18*	chr19	58596077	C|G	1676	559	P>R	missense
LPS66	*CSMD2*	chr1	33999455	C|T	9500	3167	S>F	missense
LPS66	*MUC4*	chr3	195510959	C|T	7492	2498	P>S	missense
LPS66	*WFS1*	chr4	6303549	G|A	2027	676	R>H	missense
LPS66	*WDR96*	chr10	105967431	C|T	887	296	S>L	missense
LPS66	*FAT3*	chr11	92532370	G|T	6191	2964	R>L	missense
LPS66	*TP53BP1*	chr15	43705467	G|T	5149	1717	A>S	missense
LPS66	*CDH1*	chr16	68846047	A|G	1018	340	T>A	missense
LPS66	*ZNF611*	chr19	53208684	G|A	1624	542	V>I	missense
LPS67	*FAT3*	chr11	92565111	G|T	9805	3269	A>S	missense
LPS67	*DNAH17*	chr17	76562718	C|A	1547	516	S>I	missense
LPS67	*PIK3CG*	chr7	106508826	G|A	820	274	V>I	missense
LPS67	*ROS1*	chr6	117709142	C|T	1815	605	W>X	stopgain
LPS68	*TRAPPC11*	chr4	184585165	G|C	145	49	V>L	missense
LPS68	*MDC1*	chr6	30671206	A|G	5671	1891	T>A	missense
LPS68	*DST*	chr6	56483865	G|C	4967	1656	S>T	missense
LPS68	*PLEC*	chr8	144996868	C|T	7241	2414	A>V	missense
LPS68	*OR5M3*	chr11	56237572	G|A	402	134	M>I	missense
LPS69	*DARS2*	chr1	173802601	G|A	580	194	V>I	missense
LPS69	*ITSN2*	chr2	24432835	A|C	4325	1442	Y>S	missense
LPS69	*CYTIP*	chr2	158300469	C|G	64	22	P>A	missense
LPS69	*MUC4*	chr3	195507271	C|G	11180	3727	T>S	missense
LPS69	*MUC4*	chr3	195508021	G|A	10430	477	S>N	missense
LPS69	*MUC4*	chr3	195508022	A|G	10429	3477	S>G	missense
LPS69	*MUC4*	chr3	195511061	C|T	7390	2464	P>S	missense

LPS69	*MUC4*	chr3	195511177	C|G	7274	2425	A>G	missense
LPS69	*TRAPPC11*	chr4	184622952	C|A	2954	985	S>Y	missense
LPS69	*MUC12*	chr7	100634146	C|A	302	101	A>E	missense
LPS69	*MUC12*	chr7	100634901	G|A	1057	353	G>S	missense
LPS69	*MUC12*	chr7	100635127	C|A	1283	428	S>Y	missense
LPS69	*MUC12*	chr7	100635267	A|G	1423	475	M>V	missense
LPS69	*MUC12*	chr7	100635288	G|A	1444	482	G>S	missense
LPS69	*MUC12*	chr7	100638844	C|G	5000	1667	T>S	missense
LPS69	*MUC12*	chr7	100646796	G|A	12952	4318	G>S	missense
LPS69	*MUC12*	chr7	100648048	G|A	14204	4735	S>N	missense
LPS69	*MUC12*	chr7	100648078	C|G	14234	4745	S>C	missense
LPS69	*MOGAT3*	chr7	100841509	A|T	631	211	K>X	stopgain
LPS69	*PIK3CG*	chr7	106515148	T|C	2291	764	I>T	missense
LPS69	*WDR96*	chr10	105923874	C|A	3224	1075	T>K	missense
LPS69	*PPP2R1B*	chr11	111631612	C|A	470	157	T>K	missense
LPS69	*ERBB3*	chr12	56487560	A|T	1493	498	K>I	missense
LPS69	*BTBD7*	chr14	93760935	T|C	431	144	V>A	missense
LPS69	*BTBD7*	chr14	93760936	G|A	430	144	V>I	missense
LPS69	*DNAH17*	chr17	76472697	C|T	8111	2704	T>I	missense
LPS69	*SEZ6L*	chr22	26688840	G|A	563	188	R>Q	missense
LPS70	*ITSN2*	chr2	24432835	A|C	4325	1442	Y>S	missense
LPS70	*SPHKAP*	chr2	228884308	G|A	1262	421	S>N	missense
LPS70	*FANCD2*	chr3	10107104	C|T	2195	732	P>L	missense
LPS70	*MUC4*	chr3	195512278	C|G	6173	2058	S>C	missense
LPS70	*SVIL*	chr10	29821947	A|T	1349	450	E>V	missense
LPS70	*PCDH15*	chr10	55582674	G|T	4605	1535	R>S	missense
LPS70	*HEPHL1*	chr11	93844989	C|T	3409	1137	L>F	missense
LPS70	*KIAA1033*	chr12	105531781	C|T	1444	482	L>F	missense
LPS70	*POLE*	chr12	133237641	G|A	2974	992	A>T	missense
LPS70	*ERCC4*	chr16	14042187	G|A	2734	912	G>R	missense
LPS70	*DNAAF1*	chr16	84203734	G|A	1300	434	G>R	missense
LPS71	*XIRP2*	chr2	168103475	C|T	4907	1636	C>T	missense
LPS71	*XIRP2*	chr2	168114527	A|T	1570	524	A>T	missense
LPS71	*MUC4*	chr3	195512608	G|T	5843	1948	G>V	missense
LPS71	*CDH12*	chr5	21755787	T|A	1798	600	S>T	missense
LPS71	*MOGAT3*	chr7	100843714	T|A	192	64	Y>X	stopgain
LPS71	*MXRA5*	chrX	3240977	C|T	2749	917	P>S	missense
LPS71	*COL2A1*	chr12	48372421	C|A	2647	883	P>T	missense
LPS71	*INTS6*	chr13	51943248	G|A	2303	768	G>D	missense
LPS71	*TEP1*	chr14	20836615	A|T	7865	2622	Y>F	missense
LPS71	*MAPK7*	chr17	19285598	C|T	1982	661	T>I	missense
LPS71	*DEFB125*	chr20	76700	G|A	113	38	R>Q	missense
LPS71	*DEFB125*	chr20	77044	C|T	457	153	L>F	missense
LPS71	*MUC12*	chr7	100643388	G|C	9544	3182	A>P	missense
LPS71	*HEPHL1*	chr11	93837772	G|A	2761	921	V>I	missense
LPS71	*XIRP2*	chr2	168104863	|A	6295_6296	2099	E>fs	frameshift insertion
LPS71	*PARP1*	chr1	226551692	C|T	2738	913	G>E	missense
LPS71	*NF1*	chr17	29553477	|C	2026_2027	676	T>fs	frameshift insertion
LPS72	*CSMD2*	chr1	34554603	C|G	259	87	P>A	missense
LPS72	*XIRP2*	chr2	168106406	T|C	7838	2613	L>S	missense
LPS72	*ROBO1*	chr3	78656114	C|T	4213	1405	P>S	missense
LPS72	*MUC4*	chr3	195506762	G|C	11689	3897	A>P	missense
LPS72	*MUC12*	chr7	100635849	C|G	2005	669	P>A	missense
LPS72	*PLEC*	chr8	144998515	A|V	5594	1865	A>V	missense
LPS72	*FAT3*	chr11	92531397	A|T	5218	1740	I>F	missense
LPS72	*ADAMTSL3*	chr15	84651796	G|A	3416	1139	R>Q	missense
LPS72	*SEZ6L*	chr22	26688852	C|T	575	192	A>V	missense
LPS72	*MUC4*	chr3	195511327	G|A	7124	2375	S>F	missense
LPS72	*MUC4*	chr3	195511567	G|A	6884	2295	S>F	missense
LPS72	*KRTAP10-11*	chr21	46066437	A|C	62	21	D>A	missense
LPS72	*MUC4*	chr3	195515258	C|G	3193	1065	A>P	missense
LPS72	*NF1*	chr17	29553477	|C	2026_2027	676	T>fs	frameshift insertion
LPS73	*USP48*	chr1	22055168	G|C	1345	449	E>Q	missense

LPS73	*DARS2*	chr1	173802601	G|A	580	194	V>I	missense
LPS73	*XIRP2*	chr2	168103129	G|A	4561	1521	V>I	missense
LPS73	*MUC4*	chr3	195513493	G|T	4958	1653	S>I	missense
LPS73	*MUC4*	chr3	195518119	T|A	332	111	M>K	missense
LPS73	*APC*	chr5	112103050	G|C	415	139	E>Q	missense
LPS73	*MDC1*	chr6	30672479	C|A	4481	1494	P>H	missense
LPS73	*MUC12*	chr7	100657309	A|T	15754	5252	I>F	missense
LPS73	*PLEC*	chr8	144991205	G|A	12796	4266	V>I	missense
LPS73	*FAT3*	chr11	92086345	G|A	1067	356	C>Y	missense
LPS73	*HEPHL1*	chr11	93844989	C|T	3409	1137	L>F	missense
LPS73	*DNAH17*	chr17	76503831	C|A	4293	1431	H>Q	missense
LPS73	*CHD6*	chr20	40049349	G|A	5926	1976	A>T	missense
LPS73	*HEPHL1*	chr11	93808430	A|T	1595	532	D>V	missense
LPS73	*PLBD1*	chr12	14689639	C|G	564	188	M>I	missense
LPS74	*CSMD2*	chr1	34037197	A|G	7898	2633	Q>R	missense
LPS74	*CSMD2*	chr1	34090660	A|G	5371	1791	T>A	missense
LPS74	*FANCD2*	chr3	10135980	G|A	3896	1299	G>A	missense
LPS74	*CACNA1D*	chr3	53835116	A|T	5132	1711	N>I	missense
LPS74	*MUC4*	chr3	195506186	G|T	12265	4089	A>S	missense
LPS74	*MUC4*	chr3	195506252	G|C	12199	4067	V>L	missense
LPS74	*DST*	chr6	56391265	G|A	10154	3385	G>E	missense
LPS74	*DST*	chr6	56457152	A|G	4862	1621	N>S	missense
LPS74	*ROS1*	chr6	117639393	A|G	5963	1988	D>G	missense
LPS74	*OR6B1*	chr7	143701601	C|T	512	171	P>L	missense
LPS74	*PLEC*	chr8	144993467	G|A	10534	3512	D>N	missense
LPS74	*ASB9*	chrX	15270435	G|A	374	125	G>E	missense
LPS74	*MYO3A*	chr10	26414398	G|A	1975	659	G>R	missense
LPS74	*TEP1*	chr14	20852298	C|T	3433	1145	R>W	missense
LPS74	*ADAMTSL3*	chr15	84506866	G|A	626	209	G>E	missense
LPS74	*NF1*	chr17	29552168	T|C	1901	634	I>T	missense
LPS74	*DNAH17*	chr17	76451830	G|A	10066	3356	V>I	missense
LPS74	*ZNF611*	chr19	53219129	G|C	19	7	G>C	missense
LPS74	*DNAH14*	chr1	225273310	A|-	3392	1131	E>fs	frameshift deletion
LPS74	*PCDH15*	chr10	55583015	|AATAGTATT	4351_4352	1451	delinsNTIE	nonframeshift insertion
LPS74	*SPOCK3*	chr4	167713323	C|T	422	141	S>N	missense
LPS75	*HEG1*	chr3	124731800	A|T	2623	875	T>S	missense
LPS75	*THSD7A*	chr7	11582681	A|G	1517	506	H>R	missense
LPS75	*PCDH15*	chr10	55582674	G|T	4605	1535	R>S	missense
LPS75	*NF1*	chr17	29541509	A|C	1433	478	K>T	missense
LPS75	*NF1*	chr17	29552200	A|G	1933	645	M>V	missense
LPS75	*BRCA1*	chr17	41231357	A|G	4417	473	I>V	missense
LPS75	*BRCA1*	chr17	41243886	A|C	3662	1221	E>A	missense
LPS75	*DNAH17*	chr17	76511061	A|G	3908	1303	D>G	missense
LPS75	*DNAH17*	chr17	76567763	A|G	641	214	Q>R	missense
LPS75	*LIPE*	chr19	42914568	G|A	1310	437	R>Q	missense
LPS75	*DNAH14*	chr1	225548560	C|T	11675	3892	S>L	missense
LPS75	*NF1*	chr17	29483133	G|A	193	65	V>I	missense
LPS75	*DST*	chr6	56336912	A|G	14234	4745	L>P	missense
LPS75	*ROS1*	chr6	117704637	T|C	2339	780	D>G	missense
LPS75	*ERCC1*	chr19	45924491	T|C	266	89	N>S	missense
LPS75	*MXRA5*	chrX	3238963	C|T	4763	1588	R>H	missense
LPS75	*SLITRK2*	chrX	144905516	G|A	1573	525	D>N	missense
LPS75	*MUC12*	chr7	100634331	|A	487_488	163	E>fs	frameshift insertion
LPS75	*DNAJC14*	chr12	56222203	|C	240_241	80	G>fs	frameshift insertion
LPS76	*MUC4*	chr3	195506003	C|A	12448	4150	P>T	missense
LPS76	*FRG1*	chr4	190876263	A|T	389	130	D>V	missense
LPS76	*CDH12*	chr5	22078686	G|C	100	34	E>Q	missense
LPS76	*MUC12*	chr7	100634484	C|G	640	214	P>A	missense
LPS76	*TEP1*	chr14	20847140	G|A	5252	1751	R>Q	missense
LPS76	*TEP1*	chr14	20849515	G|A	4604	1535	R>Q	missense
LPS76	*DNAJC14*	chr12	56221395	C|G	1048	350	V>L	missense
LPS77	*DNAH14*	chr1	225268106	C|A	2792	931	A>D	missense
LPS77	*SEMA3A*	chr7	83634712	G|A	1303	435	V>I	missense

LPS77T	*DEFB135*	chr8	11839887	C|T	58	20	P>S	missense
LPS77T	*INTS6*	chr13	51943288	C|G	2263	755	L>V	missense
LPS77	*FRG1*	chr4	190878646	G|T	526	176	E>X	stopgain SNV
LPS77	*FOLR2*	chr11	71929727	T|A	99	33	D>E	missense
LPS77	*FANCA*	chr16	89807226	G|A	3814	1272	H>Y	missense
LPS77	*ZNF611*	chr19	53208257	C|A	2051	684	G>V	missense
LPS77	*TP53*	chr17	7577018	C|T		splicing	splicing	splicing
LPS78	*RYR2*	chr1	237802485	G|A	7099	2367	G>R	missense
LPS78	*MUC4*	chr3	195507214	C|A	11237	3746	P>H	missense
LPS78	*MUC4*	chr3	195508451	C|A	10000	3334	P>T	missense
LPS78	*MUC4*	chr3	195508453	G|A	9998	3333	S>N	missense
LPS78	*SEC63*	chr6	108279199	G|C	15	5	Q>H	missense
LPS78	*MGAM*	chr7	141750579	C|T	2720	907	T>M	missense
LPS78	*MGAM*	chr7	141803124	T|C	5381	1794	L>S	missense
LPS78	*CSMD3*	chr8	114448951	A|G	133	45	T>A	missense
LPS78	*PLEC*	chr8	145009036	G|A	899	300	R>Q	missense
LPS78	*DNAAF1*	chr16	84199444	C|G	919	307	Q>E	missense
LPS78	*NF1*	chr17	29552168	T|C	1901	634	I>T	missense
LPS78	*MUC4*	chr3	195510785	C|T	7666	2556	G>S	missense
LPS78	*CSMD3*	chr8	114448951	T|C	133	45	T>A	missense
LPS78	*ZBTB16*	chr11	113935229	G|A	1207	403	G>R	missense
LPS78	*MUC4*	chr3	195508324	C|T	10127	3376	R>H	missense
LPS78	*STAT6*	chr12	57493136	T|A	1502	501	Q>L	missense
LPS78	*MUC4*	chr3	195511889	C|T	6562	2188	G>S	missense
LPS78	*MUC4*	chr3	195511985	C|T	6466	2156	G>S	missense
LPS78	*MUC4*	chr3	195509423	G|A	9028	3010	P>S	missense
LPS78	*CSMD2*	chr1	34181993	C|T	2990	997	R>Q	missense
LPS78	*MUC4*	chr3	195507185	C|T	11266	3756	G>S	missense
LPS79	*VEPH1*	chr3	157221258					missense
LPS79	*DST*	chr6	56480510	A|G	7755	2585	I>M	missense
LPS79	*DST*	chr6	56481130	G|A	7135	2379	A>T	missense
LPS79	*PTPRD*	chr9	8500931	G|C	1951	651	A>P	missense
LPS79	*STOX1*	chr10	70644912	A|G	1360	454	K>E	missense
LPS79	*TP53BP1*	chr15	43713320	C|A	4153	1385	D>Y	missense
LPS79	*EGFR*	chr7	55238221	CCT|-	2102_2104	701_702	del	nonframeshift deletion
LPS79	*MUC12*	chr7	100635316	A|G	1472	491	E>G	missense
LPS79	*DST*	chr6	56481130	C|T	7135	2379	A>T	missense
LPS79	*MUC12*	chr7	100635318	A|C	1474	492	K>Q	missense
LPS79	*PLEC*	chr8	145012372	G|A	169	57	R>C	missense
LPS79	*FAT3*	chr11	92086371	G|A	1093	365	G>S	missense
LPS80	*DOCK7*	chr1	63099204	G|A	1081	361	P>S	missense
LPS80	*SYT6*	chr1	114680443	G|C	745	249	R>G	missense
LPS80	*WDR66*	chr12	122439498	G|A	3331	1111	V>I	missense
LPS80	*TPP2*	chr13	103301494	G|C	2866	956	D>H	missense
LPS80	*KCNK10*	chr14	88658663	A|G	758	253	V>A	missense
LPS80	*B2M*	chr15	45003745	A|G	1	1	M>V	missense
LPS80	*HDGFRP3*	chr15	83820013	T|C	560	187	N>S	missense
LPS80	*IQGAP1*	chr15	91026781	A|C	3744	1248	L>F	missense
LPS80	*DNAAF1*	chr16	84182654	C|T	167	56	T>I	missense
LPS80	*STARD3*	chr17	37815053	C|T	572	191	S>L	missense
LPS80	*COG1*	chr17	71193530	C|G	908	303	P>R	missense
LPS80	*TSN*	chr2	122522834	A|G	578	193	K>R	missense
LPS80	*KRTAP10-11*	chr21	46066767	G|T	392	131	S>I	missense
LPS80	*SEMA3G*	chr3	52474021	G|A	1237	413	L>F	missense
LPS80	*CDH12*	chr5	21752012	G|A	2219	740	A>V	missense
LPS80	*MGAM*	chr7	141727465	G|A	1151	384	R>H	missense
LPS80	*PLEC*	chr8	144995168	G|C	8779	2927	L>V	missense
LPS80	*ASB9*	chrX	15268665	G|A	455	152	S>F	missense
LPS80	*FAM47C*	chrX	37027222	C|T	739	247	R>C	missense
LPS80	*FMR1NB*	chrX	147084825	T|G	382	128	F>V	missense
LPS80	*NDUFA9*	chr12	4778952	G|A	769	257	D>N	missense
LPS80	*COL2A1*	chr12	48372514	C|T	2554	852	G>S	missense
LPS80	*MCTS1*	chrX	119740017	G|C	251	84	R>T	missense
LPS80	*CYTIP*	chr2	158300390	G|A	143	48	T>M	missense

LPS80	*DNAH14*	chr1	225452889	C|T	7210	2404	P>S	missense
LPS80	*ZNF280C*	chrX	129370191	C|T	768	256	M>I	missense
LPS80	*PPFIA2*	chr12	81693145	C|T	1360	454	E>K	missense
LPS80	*XIRP2*	chr2	168104657	A|G	6089	2030	D>G	missense
LPS80	*RYR2*	chr1	237617829	C|-	1431	477	N>fs	frameshift deletion
LPS80	*SVIL*	chr10	29762864	C|T	4154	1385	C>Y	missense
LPS80	*MUC4*	chr3	195509353	G|A	9098	3033	A>V	missense
LPS80	*CSMD2*	chr1	34003051	G|T	9358	3120	Q>K	missense
LPS80	*MUC4*	chr3	195509354	C|T	9097	3033	A>T	missense
LPS81	*MUC4*	chr3	195508283	G|A	10168	3390	A>T	missense
LPS81	*MUC4*	chr3	195511012	C|G	7439	2480	P>R	missense
LPS81	*MUC4*	chr3	195513650	C|G	4801	1601	L>V	missense
LPS81	*THSD7A*	chr7	11419307	G|A	4540	1514	D>N	missense
LPS81	*SEMA3A*	chr7	83590938	A|G	2065	689	K>E	missense
LPS81	*PLEC*	chr8	144991208	G|A	12793	4265	A>T	missense
LPS81	*PLEC*	chr8	145008167	C|T	1274	425	S>L	missense
LPS81	*PCDH15*	chr10	55582674	G|T	4605	153	R>S	missense
LPS81	*PDE3A*	chr12	20783043	C|T	776	259	P>L	missense
LPS81	*STAT6*	chr12	57499258	G|A	805	269	V>I	missense
LPS81	*TP53BP1*	chr15	43769812	T|G	934	312	F>V	missense
LPS81	*MYH3*	chr17	10554922	G|A	412	138	E>K	missense
LPS81	*FLT1*	chr13	28979920	A|C	1548	516	N>K	missense
LPS81	*MUC4*	chr3	195513650	G|C	4801	1601	L>V	missense
LPS81	*TP53BP1*	chr15	43769812	A|C	934	312	F>V	missense
LPS81	*PLEC*	chr8	144997673	C|T	6382	2128	A>T	missense
LPS81	*PLEC*	chr8	145008167	G|A	1220	407	S>L	missense
LPS81	*PDE3A*	chr12	20803518	A|G	1943	648	N>S	missense
LPS82	*DNAH14*	chr1	225440018	C|T	6746	2249	T>I	missense
LPS82	*USP34*	chr2	61633098	T|A	297	99	N>K	missense
LPS82	*MUC4*	chr3	195507876	C|G	10575	3525	D>E	missense
LPS82	*MUC4*	chr3	195509401	T|C	9050	3017	I>T	missense
LPS82	*MUC4*	chr3	195510821	T|C	7630	2544	S>P	missense
LPS82	*MUC4*	chr3	195513755	A|T	4696	1566	T>S	missense
LPS82	*MUC4*	chr3	195515383	T|C	3068	1023	T>N	missense
LPS82	*DST*	chr6	56482923	T|C	5909	1970	F>S	missense
LPS82	*DOPEY1*	chr6	83877848	G|A	7360	2454	V>I	missense
LPS82	*BRAF*	chr7	140434542	G|A	2156	719	R>H	missense
LPS82	*DPP6*	chr7	154561192	G|A	949	317	A>T	missense
LPS82	*PLEC*	chr8	145004447	A|G	2489	830	D>G	missense
LPS82	*MYO3A*	chr10	26491987	C|T	4681	1561	R>X	stopgain
LPS82	*SVIL*	chr10	29821683	C|T	1613	538	S>L	missense
LPS82	*FAT3*	chr11	92086345	G|A	1067	356	C>Y	missense
LPS82	*ADAMTSL3*	chr15	84488678	C|T	479	160	P>L	missense
LPS82	*ANKS3*	chr16	4755206	A|G	437	146	Q>R	missense
LPS82	*OTOP3*	chr17	72939759	A|C	745	249	N>H	missense
LPS82	*ST8SIA3*	chr18	55024205	C|T	364	122	R>W	missense
LPS82	*E2F1*	chr20	32268137	G|T	347	116	G>V	missense
LPS82	*MUC4*	chr3	195509989	T|A	8462	2821	D>V	missense
LPS82	*MUC4*	chr3	195513304	|TGACAGGAA	5147_5148	1716	delins	nonframeshift insertion
LPS82	*GLI1*	chr12	57864118	G|A	1211	404	R>H	missense
LPS82	*SACS*	chr13	23929088	C|A	1663	555	E>X	stopgain SNV
LPS82	*E2F1*	chr20	32268137	C|A	347	116	G>V	missense
LPS83	*SYT6*	chr1	114640467	G|A	1142	381	R>H	missense
LPS83	*FCAMR*	chr1	207133867	C|A	1354	452	L>I	missense
LPS83	*HTR1F*	chr3	88040035	G|A	136	46	A>T	missense
LPS83	*EGF*	chr4	110866425	G|A	934	312	E>K	missense
LPS83	*FER1L6*	chr8	125072527	T|G	2981	994	V>G	missense
LPS83	*NEUROD4*	chr12	55420891	A|G	668	223	H>R	missense
LPS83	*ADAMTSL3*	chr15	84651057	G|A	2677	893	E>K	missense
LPS83	*DNAH17*	chr17	76451830	G|A	10066	3356	V>I	missense
LPS83	*PCDH15*	chr10	55582230	AGG|-	5134_5136	1712_1712	del	nonframeshift deletion
LPS83	*ZNF169*	chr9	97062740	|TA	900_901	300	R>fs	frameshift insertion
LPS84	*STAT1*	chr2	191848450	T|C	1364	455	V>A	missense

LPS84	*MUC4*	chr3	195506014	C|T	12437	4146	P>L	missense
LPS84	*FRG1*	chr4	190873398	C|T	215	72	A>V	missense
LPS84	*FRG1*	chr4	190873412	C|A	229	77	L>I	missense
LPS84	*FRG1*	chr4	190876263	A|T	389	130	D>V	missense
LPS84	*FRG1*	chr4	190878625	A|G	505	169	S>G	missense
LPS84	*MGAM*	chr7	141803090	A|G	54347	1783	A>V	missense
LPS84	*PCDH15*	chr10	55582636	A|G	4643	1548	N>S	missense
LPS84	*OR8H2*	chr11	55872943	T|C	425	142	L>P	missense
LPS84	*MMAB*	chr12	109999612	T|C	394	132	C>R	missense
LPS84	*LLGL1*	chr17	18133277	A|G	104	35	N>S	missense
LPS85	*MUC4*	chr3	195509378	C|G	9073	3025	L>V	missense
LPS85	*MUC4*	chr3	195512515	C|A	5936	1979	T>K	missense
LPS85	*MUC4*	chr3	195518120	A|C	331	111	M>L	missense
LPS85	*DNAH17*	chr17	76451830	G|A	10066	3356	V>I	missense
LPS85	*PARP1*	chr1	226578123	A|G	605	202	V>A	missense
LPS85	*PTEN*	chr10	89711993	C|A	611	204	P>Q	missense
LPS85	*PTEN*	chr10	89711992	C|-	610	204	P>fs	frameshift deletion
LPS86	*NF1*	chr17	29664385	G|A				
LPS86	*ANKS3*	chr16	4764124	C|A	316	106	G>X	stopgain SNV
LPS86	*MUC4*	chr3	195509287	T|G	9164	3055	N>T	missense
LPS86	*MUC4*	chr3	195509401	A|G	9050	3017	I>T	missense
LPS86	*CFD*	chr19	863213	C|A	737	246	A>D	missense
LPS87	*XIRP2*	chr2	168115133	G|-	1411	471	D>fs	frameshift deletion
LPS87	*PCDH15*	chr10	55782929	C|A	2036	679	G>V	missense
LPS87	*NF1*	chr17	29490391	C|A	476	159	T>N	missense
LPS87	*WDR96*	chr10	105893514	A|G	4460	1487	M>T	missense
LPS87	*MXRA5*	chrX	3235780	|G	5942_5943	1981	Q>fs	frameshift insertion
LPS88	*DOCK7*	chr1	63113391	T|G	789	263	Q>H	missense
LPS88	*DOCK4*	chr7	111448904	C|A	3139	1047	E>X	stopgain SNV
LPS88	*ATR*	chr3	142178188	C|A	7230	2410	M>I	missense
LPS88	*CGNL1*	chr15	57836768	T|G	3473	1158	L>R	missense
LPS88	*ATM*	chr11	108203547	T|C	7847	2616	M>T	missense
LPS88	*POLE*	chr12	133201355	C|A	6789	2263	Q>H	missense
LPS88	*PLCB4*	chr20	9318662	A|T	173	58	Q>L	missense
LPS88	*MUC4*	chr3	195518120	T|G	331	111	M>L	missense
LPS89	*DNAH17*	chr17	76566356	C|A	1017	339	Q>H	missense
LPS89	*FRG1*	chr4	190876269	T|C	395	132	I>T	missense
LPS89	*DOCK7*	chr1	62971414	C|A	4457	1486	C>F	missense
LPS89	*DOCK7*	chr1	62971415	A|T	4456	1486	C>S	missense
LPS89	*DNAH17*	chr17	76450596	|T	10362_10363	3454	N>fs	frameshift insertion
LPS89	*MUC12*	chr7	100643388	G|A	9544	3182	A>T	missense
LPS90	*GLI1*	chr12	57865027	C|T	2120	707	A>V	missense
LPS90	*FRG1*	chr4	190876269	T|C	395	132	I>T	missense

LPS cell lines

Sample ID	Gene Symbol	Chromosome	Genomic position	Nucleotide change (reference|variant)	cDNA position	Amino acid position	Amino acid change	Mutation type
LPS141	*MUC4*	chr3	195515510	A|C	2941	981	Y>D	missense
LPS141	*PTEN*	chr10	89720683	C|G	834	278	F>L	missense
LPS141	*HEPHL1*	chr11	93806252	C|T	1294	432	R>W	missense
LPS141	*RB1*	chr13	49050886	G|A	2570	857	R>H	missense
LPS141	*TP53BP1*	chr15	43748890	C|T	1916	639	R>Q	missense
LPS141	*DNAH17*	chr17	76491162	A|C	5921	1974	V>G	missense
LP6	*FANCD2*	chr3	10094159	A|G	1634	545	N>S	missense
LP6	*FRG1*	chr4	190876269	T|C	395	132	I>T	missense
LP6	*FRG1*	chr4	190883030	G|T	683	228	S>I	missense
LP6	*PLEC*	chr8	144993062	C|T	10939	3647	V>M	missense

LP6	*OR8H2*	chr11	55872943	T|C	425	142	L>P	missense
LP6	*AGAP2*	chr12	58120879	G|T	2146	716	L>M	missense
LP6	*HCAR2*	chr12	123187079	C|T	752	251	R>Q	missense
LP6	*SACS*	chr13	23910328	A|C	7687	2563	F>V	missense
LP6	*ROBO1*	chr3	78676694	C□T	3352	1118	V>M	missense
LP6	*WFS1*	chr4	6303405	C|T	1883	628	T>M	missense
LP6	*AGAP2*	chr12	58127878	G|A	1480	494	H>Y	missense
LP6	*OR5M3*	chr11	56237609	A|G	365	122	M>T	missense
SW872	*PPARG*	chr3	12458293	G|A	826	276	E>K	missense
SW872	*ATR*	chr3	142178118	G|A	7300	2434	P>S	missense
SW872	*EGF*	chr4	110895876	G|A	1616	539	R>K	missense
SW872	*DST*	chr6	56376091	C|T	10815	3605	E>D	missense
SW872	*THSD7A*	chr7	11871406	G|C	167	56	P>R	missense
SW872	*MUC12*	chr7	100634887	G|A	1043	348	R>H	missense
SW872	*BRAF*	chr7	140453136	A|T	1799	600	V>E	missense
SW872	*MGAM*	chr7	141708471	G|A	293	98	R>Q	missense
SW872	*CDKN2A*	chr9	21971120	G|A	238	80	R>X	stopgain
SW872	*CDKN2A*	chr9	21971121	G|A	280	94	P>S	missense
SW872	*FAT3*	chr11	92534809	C|T	8630	2877	T>I	missense
SW872	*FAT3*	chr11	92577352	G|A	10819	3607	A>T	missense
SW872	*TP53*	chr17	7577529	A|T	356	119	I>N	missense
SW872	*DNAH17*	chr17	76482548	C|T	6859	2287	E>K	missense
SW872	*ZNF135*	chr19	58578642	C|T	826	276	H>Y	missense
SW872	*RNF32*	chr7	156468392	C|G	687	229	F>L	missense
SW872	*PLEC*	chr8	144991082	C|T	12865	4289	V>M	missense
SW872	*HEG1*	chr3	124739948	T|C	940	314	N>D	missense
SW872	*SEMA3A*	chr7	83634712	C|T	1303	435	V>I	missense
SW872	*MOGAT3*	chr7	100843532	C|T	271	91	D>N	missense
SW872	*LIPE*	chr19	42930734	G|T	568	190	P>T	missense
SW872	*ZNF611*	chr19	53209906	C|G	402	134	K>N	missense
T778	*DNAH14*	chr1	225270424	A|T	3310	1104	N>Y	missense
T778	*XIRP2*	chr2	168099316	T|C	1414	472	S>A	missense
T778	*MUC4*	chr3	195506674	A|G	11777	3926	V>A	missense
T778	*EGF*	chr4	110885680	C|G	1436	479	P>H	missense
T778	*MAGEB6*	chrX	26212695	G|T	732	244	L>F	missense
T778	*ERBB2*	chr17	37864647	G|A	209	70	R>Q	missense
T778	*CHEK2*	chr22	29130456	G|C	254	85	P>R	missense
T1000	*DNAH14*	chr1	225492710	C|T	8645	2882	T>I	missense
T1000	*ROBO1*	chr3	78710202	C|T	2298	766	K>N	missense
T1000	*MUC4*	chr3	195512412	C|A	6039	2013	Q>H	missense
T1000	*APC*	chr5	112174456	A|T	3111	1037	I>M	missense
T1000	*MUC12*	chr7	100635210	T|C	1366	456	S>P	missense
T1000	*MUC12*	chr7	100648360	C|A	14516	4839	A>D	missense
T1000	*DOCK4;DOCK4*	chr7	111580166	T|C	976	326	M>V	missense
T1000	*PLEC*	chr8	145005769	C|T	2249	750	R>H	missense
T1000	*MXRA5*	chrX	3248104	C|	664	222	D>N	missense
T1000	*PCDH15*	chr10	55700673	G|T	2972	991	A>V	missense
T1000	*ERBB3*	chr12	56493677	A|G	2993	998	K>T	missense
T1000	*STAT6*	chr12	57490504	C|T	2395	799	E>K	missense
SA4	*FCAMR*	chr1	207135723	G|A	487	163	H>Y	missense
SA4	*DNAH14*	chr1	225267125	A|T	2641	881	S>C	missense
SA4	*MUC4*	chr3	195506674	A|G	11777	3926	V>A	missense
SA4	*MUC4*	chr3	195506692	C|G	11759	3920	R>P	missense
SA4	*MUC4*	chr3	195506725	T|A	11726	3909	D>G	missense
SA4	*SPOCK3*	chr4	167656203	C|T	904	302	D>N	missense
SA4	*DST;DST*	chr6	56350110	C|A	13338	4446	K>N	missense
SA4	*BRAF*	chr7	140453136	A|T	1799	600	V>E	missense
SA4	*MXRA5*	chrX	3229524	C|T	6720	2240	M>I	missense
SA4	*MYO3A*	chr10	26436473	C|T	2620	874	P>S	missense
SA4	*STAT6*	chr12	57500533	C|T	421	141	V>I	missense
SA4	*FLT1*	chr13	29007999	G|A	770	257	P>L	missense
SA4	*DNAAF1*	chr16	84203788	C|A	1354	452	P>T	missense
LP1	*CSMD2*	chr1	34066566	C|T	6761	2254	R>Q	missense
LP1	*MUC4*	chr3	195506911	G|C	11540	3847	S>F	missense
LP1	*MUC12*	chr7	100636722	G|C	2878	960	V>L	missense
LP1	*MGAM*	chr7	141763311	C|A	4270	1424	P>T	missense

<table>
<tr><td>LP1</td><td>PTEN</td><td>chr10</td><td>89692790</td><td>G|A</td><td>274</td><td>92</td><td>D>N</td><td>missense</td></tr>
<tr><td>LP1</td><td>OR5M3</td><td>chr11</td><td>56237502</td><td>T|A</td><td>472</td><td>158</td><td>T>S</td><td>missense</td></tr>
<tr><td>LP1</td><td>DLG2</td><td>chr11</td><td>84822734</td><td>G|C</td><td>328</td><td>110</td><td>P>A</td><td>missense</td></tr>
<tr><td>LP1</td><td>ROBO1</td><td>chr3</td><td>78655944</td><td>T|G</td><td>4683</td><td>1561</td><td>K>N</td><td>missense</td></tr>
<tr><td>LP2</td><td>CSMD2</td><td>chr1</td><td>33985463</td><td>G|A</td><td>10253</td><td>3418</td><td>P>L</td><td>missense</td></tr>
<tr><td>LP2</td><td>CSMD2</td><td>chr1</td><td>34066566</td><td>C|T</td><td>6761</td><td>2254</td><td>R>Q</td><td>missense</td></tr>
<tr><td>LP2</td><td>MUC12</td><td>chr7</td><td>100636722</td><td>G|C</td><td>2878</td><td>960</td><td>V>L</td><td>missense</td></tr>
<tr><td>LP2</td><td>MGAM</td><td>chr7</td><td>141763311</td><td>C|A</td><td>4270</td><td>1424</td><td>P>T</td><td>missense</td></tr>
<tr><td>LP2</td><td>OR6B1</td><td>chr7</td><td>143701522</td><td>G|A</td><td>433</td><td>145</td><td>A>T</td><td>missense</td></tr>
<tr><td>LP2</td><td>OR5M3</td><td>chr11</td><td>56237502</td><td>T|A</td><td>472</td><td>158</td><td>T>S</td><td>missense</td></tr>
<tr><td>LP2</td><td>DLG2</td><td>chr11</td><td>84822734</td><td>G|C</td><td>328</td><td>110</td><td>P>A</td><td>missense</td></tr>
<tr><td>LP3</td><td>ROBO1</td><td>chr3</td><td>78655944</td><td>T|G</td><td>4683</td><td>1561</td><td>K>N</td><td>missense</td></tr>
<tr><td>LP3</td><td>CSMD2</td><td>chr1</td><td>33985463</td><td>G|A</td><td>10253</td><td>3418</td><td>P>L</td><td>missense</td></tr>
<tr><td>LP3</td><td>CSMD2</td><td>chr1</td><td>34066566</td><td>C|T</td><td>6761</td><td>2254</td><td>R>Q</td><td>missense</td></tr>
<tr><td>LP3</td><td>MUC4</td><td>chr3</td><td>195506674</td><td>A|G</td><td>11777</td><td>3926</td><td>V>A</td><td>missense</td></tr>
<tr><td>LP3</td><td>MUC4</td><td>chr3</td><td>195506692</td><td>C|G</td><td>11759</td><td>3920</td><td>R>P</td><td>missense</td></tr>
<tr><td>LP3</td><td>MUC4</td><td>chr3</td><td>195506911</td><td>G|C</td><td>11540</td><td>3847</td><td>S>F</td><td>missense</td></tr>
<tr><td>LP3</td><td>MUC12</td><td>chr7</td><td>100636722</td><td>G|C</td><td>2878</td><td>960</td><td>V>L</td><td>missense</td></tr>
<tr><td>LP3</td><td>MGAM</td><td>chr7</td><td>141763311</td><td>C|A</td><td>4270</td><td>1424</td><td>P>T</td><td>missense</td></tr>
<tr><td>LP3</td><td>PTEN</td><td>chr10</td><td>89692790</td><td>G|A</td><td>274</td><td>92</td><td>D>N</td><td>missense</td></tr>
<tr><td>LP3</td><td>OR5M3</td><td>chr11</td><td>56237502</td><td>T|A</td><td>472</td><td>158</td><td>T>S</td><td>missense</td></tr>
<tr><td>LP3</td><td>DLG2</td><td>chr11</td><td>84822734</td><td>G|C</td><td>328</td><td>110</td><td>P>A</td><td>missense</td></tr>
<tr><td>MLS402</td><td>HEG1</td><td>chr3</td><td>124732563</td><td>C|A</td><td>1860</td><td>620</td><td>Q>H</td><td>missense</td></tr>
<tr><td>MLS402</td><td>MUC12</td><td>chr7</td><td>100634386</td><td>C|T</td><td>542</td><td>181</td><td>P>L</td><td>missense</td></tr>
<tr><td>MLS402</td><td>INTS6</td><td>chr13</td><td>51943320</td><td>A|G</td><td>2231</td><td>744</td><td>L>S</td><td>missense</td></tr>
<tr><td>FUDDLS1</td><td>ERBB4</td><td>chr2</td><td>212288969</td><td>G|A</td><td>2777</td><td>926</td><td>T>M</td><td>missense</td></tr>
<tr><td>FUDDLS1</td><td>WFS1</td><td>chr4</td><td>6303731</td><td>G|A</td><td>2209</td><td>737</td><td>E>K</td><td>missense</td></tr>
<tr><td>FUDDLS1</td><td>MDC1</td><td>chr6</td><td>30673725</td><td>G|A</td><td>3235</td><td>1079</td><td>R>S</td><td>missense</td></tr>
<tr><td>FUDDLS1</td><td>DST</td><td>chr6</td><td>56347491</td><td>G|C</td><td>13523</td><td>4508</td><td>A>G</td><td>missense</td></tr>
<tr><td>FUDDLS1</td><td>CSMD3</td><td>chr8</td><td>113275939</td><td>T|G</td><td>9284</td><td>3095</td><td>E>A</td><td>missense</td></tr>
<tr><td>FUDDLS1</td><td>MXRA5</td><td>chrX</td><td>3229104</td><td>C|T</td><td>7140</td><td>2380</td><td>W>X</td><td>stopgain</td></tr>
<tr><td>FUDDLS1</td><td>GLI1</td><td>chr12</td><td>57859693</td><td>A|G</td><td>747</td><td>249</td><td>Q>H</td><td>missense</td></tr>
<tr><td>GOT3</td><td>MDC1</td><td>chr6</td><td>30672567</td><td>C|G</td><td>4393</td><td>1465</td><td>V>L</td><td>missense</td></tr>
<tr><td>GOT3</td><td>PTCHD4</td><td>chr6</td><td>47976616</td><td>T|C</td><td>661</td><td>221</td><td>S>G</td><td>missense</td></tr>
<tr><td>GOT3</td><td>MUC12</td><td>chr7</td><td>100636543</td><td>C|G</td><td>2699</td><td>900</td><td>T>S</td><td>missense</td></tr>
<tr><td>GOT3</td><td>PLEC</td><td>chr8</td><td>144990784</td><td>G|A</td><td>13217</td><td>4406</td><td>T>M</td><td>missense</td></tr>
<tr><td>GOT3</td><td>PLEC</td><td>chr8</td><td>145009036</td><td>C|T</td><td>899</td><td>300</td><td>R>Q</td><td>missense</td></tr>
<tr><td>GOT3</td><td>SACS</td><td>chr13</td><td>23929138</td><td>G|A</td><td>1613</td><td>538</td><td>A>V</td><td>missense</td></tr>
<tr><td>GOT3</td><td>BTBD7</td><td>chr14</td><td>93760826</td><td>C|G</td><td>540</td><td>180</td><td>E>D</td><td>missense</td></tr>
<tr><td>GOT3</td><td>DNAH17</td><td>chr17</td><td>76490147</td><td>C|T</td><td>6379</td><td>2127</td><td>V>I</td><td>missense</td></tr>
<tr><td>LiSa-2</td><td>PADI2</td><td>chr1</td><td>17413152</td><td>C|T</td><td>698</td><td>233</td><td>R>L</td><td>missense</td></tr>
<tr><td>LiSa-2</td><td>ADAMTS4</td><td>chr1</td><td>161160941</td><td>G|C</td><td>2501</td><td>834</td><td>A>G</td><td>missense</td></tr>
<tr><td>LiSa-2</td><td>MUC4</td><td>chr3</td><td>195513215</td><td>G|A</td><td>5236</td><td>1746</td><td>L>V</td><td>missense</td></tr>
<tr><td>LiSa-2</td><td>MUC4</td><td>chr3</td><td>195518002</td><td>T|C</td><td>449</td><td>150</td><td>N>S</td><td>missense</td></tr>
<tr><td>LiSa-2</td><td>DOPEY1</td><td>chr6</td><td>83841919</td><td>C|T</td><td>2614</td><td>872</td><td>Q>X</td><td>stopgain</td></tr>
<tr><td>LiSa-2</td><td>MUC12</td><td>chr7</td><td>100635210</td><td>T|C</td><td>1366</td><td>456</td><td>S>P</td><td>missense</td></tr>
<tr><td>LiSa-2</td><td>TP53</td><td>chr17</td><td>7573984</td><td>A|G</td><td>926</td><td>309</td><td>L>S</td><td>missense</td></tr>
</table>

True call validated by Sanger Sequencing
False call validated by Sanger Sequencing
Sanger Sequencing not done

Supplementary Table S9: List of significantly mutated genes identified by MuSiC analysis

Gene	Indels	SNVs	Total Muts	Covd Bps	Muts pMbp	P-value FCPT	P-value LRT	P-value CT	FDR FCPT	FDR LRT	FDR CT
MXRA5	0	26	26	756562	34.37	1.03E-12	1.47E-14	6.66E-17	2.33E-10	3.31E-12	1.51E-14
ZNF611	0	14	14	390420	35.86	2.33E-07	1.35E-08	1.97E-10	2.64E-05	1.02E-06	2.22E-08
PLEC	0	26	26	1362365	19.08	5.81E-06	5.01E-09	2.44E-09	0.000437856	5.66E-07	1.84E-07
XIRP2	3	15	18	1166449	15.43	1.95E-05	1.06E-05	8.09E-08	0.001102237	0.000399319	4.57E-06
STARD3	2	8	10	476260	21	4.94E-05	2.24E-07	1.62E-07	0.00223313	1.27E-05	6.12E-06
NF1	6	11	17	1746222	9.74	6.68E-05	8.30E-06	1.49E-07	0.002516164	0.000375227	6.12E-06
FAT3	2	19	21	1703804	12.33	0.000573899	0.00295259	7.68E-06	0.018528728	0.035120278	0.00024793
SPHKAP	0	11	11	653608	16.83	0.005531814	0.001146382	0.000129552	0.156273757	0.018505873	0.003659843
MDC1	0	11	11	759489	14.48	0.009455728	3.85E-05	0.000215403	0.234363448	0.001086464	0.005288276
HEPHL1	0	9	9	602151	14.95	0.011534201	5.60E-05	0.000233995	0.236975408	0.001405378	0.005288276
MGAM	0	17	17	1600972	10.62	0.010370064	0.002950477	0.000364352	0.234363448	0.035120278	0.007485777
TP53	1	6	7	344578	20.31	0.027307104	0.006070038	0.000527231	0.474723496	0.0552944	0.009165717
PCDH15	2	11	13	1530462	8.49	0.018530316	0.010433525	0.000522667	0.348987615	0.073686768	0.009165717
NEUROD4	0	4	4	118515	33.75	0.079929696	0.00016416	0.000758615	1	0.003372739	0.012246211
UBQLNL	0	3	3	122919	24.41	0.145750004	3.12E-05	0.001685372	1	0.001008792	0.025392937
ZNF135	0	8	8	498534	16.05	0.05374323	0.011672432	0.002005476	0.867569285	0.077587341	0.028327349
DNAAF1	0	6	6	336255	17.84	0.079931809	0.000108421	0.002194226	1	0.002450314	0.029170294
TTI1	0	6	6	371274	16.16	0.108401672	0.001972784	0.003418823	1	0.027865571	0.042925227
MIA2	0	4	4	251693	15.89	0.228335575	0.007010551	0.005995344	1	0.060937869	0.071313035
PLBD1	1	3	4	265606	15.06	0.255677525	0.007530141	0.008645107	1	0.061138611	0.097689705
ABCC3	0	10	10	853063	11.72	0.121849182	0.048782138	0.01047964	1	0.194122872	0.112780892
CSMD3	0	9	9	1854505	4.85	0.273033218	0.000568406	0.012632558	1	0.009881527	0.118673066
ZNF280C	0	4	4	371894	10.76	0.303102928	0.001596326	0.013127552	1	0.024051316	0.118673066
ADAMTSL3	0	7	7	767144	9.12	0.184079945	0.01286249	0.012660999	1	0.08278245	0.118673066
FRG1	0	3	3	211978	14.15	0.41294549	0.003708376	0.015272651	1	0.039909188	0.127837744
CHEK2	0	5	5	324864	15.39	0.279267999	0.010291809	0.014872484	1	0.073686768	0.127837744
SACS	0	8	8	1317756	6.07	0.23812534	0.010978076	0.017553228	1	0.075183185	0.141679626

Supplementary Table S10: KEGG pathway analysis

Genes altered in patients	KEGG pathway	KEGG ID	P value	FDR	Drugs
EGF(5); *EGFR*(3); *TP53* (6); *NF1* (14); *MAPK7* (2); *AKT1* (1); *CACNAID* (3); *BRAF* (2)	MAPK signaling pathway	hsa04010	1.7852E-14	2.22E-14	Cetuximab, Dasatinib , Erlotinib hydrochloride, Lapatinib tosilate hydrate, Golimumab, Pazopanib hydrochloride, Sorafenib tosilate, Sunitinib malate, Vandetanib, Motesanib, Afatinib maleate and more
PIK3CA (5); *TP53* (6); *PI3KCD* (2); *ATM* (6); *AKT1* (1); *PIK3CG* (2);	Apoptosis	hsa04210	3.6097E-13	5.15E-13	Etanercept, Adalimumab, Infliximab, Anakinra, Certolizumab pegol, Daniplestim, Leridistim, Rilonacept
STAT6 (6); *PI3KCA* (5); *STAT5A* (4); *PI3KCD* (2); *AKT1* (1); *STAT1* (2); *PI3KCG* (2);	Jak-STAT signaling pathway	hsa04630	2.4921E-11	4.49E-11	Thiamazole, Propylthiouracil, Interferons, Tocilizumab, Somatropin, Basiliximab, Epoetins, Filgrastim, Daclizumab, Daniplestim, Darbepoetin alfa, Lestaurtinib, Ruxolitinib phosphate, Tofacitinib and more
ERBB2 (3); *ERBB3* (2); *ERBB4* (1); *PIK3CA* (5); *PIK3CD* (2); *PIK3CG* (2); *AKT1* (1); *STAT5A* (4); *MTOR* (1); *BRAF* (2); *EGFR* (3); *EGF* (5)	ErbB signaling pathway	hsa04012	3.1497E-11	3.96E-11	Gefitinib, Bosutinib, Trastuzumab, Cetuximab, Dasatinib, Erlotinib hydrochloride, Lapatinib tosilate hydrate, Mubritinib, Panitumumab, Pelitinib, Pertuzumab, Temsirolimus and more
BRCA1 (3); *BRCA2* (4); *FANCA* (7); *ERCC1* (1); *ERCC4* (3); *FANCB* (1); *FANCD2* (3); *TELO2* (5)	Fanconi anemia pathway	hsa03460	1.8189E-07	2.24E-07	
APC (6); *PLCB4* (2); *PLCB3* (2); *TP53* (6); *PPP2R1B* (4)	Wnt signaling pathway	hsa04310	4.0793E-07	6.01E-07	
CHEK2 (5); *ATM* (6); *TP53* (6); *PTEN* (3)	p53 signaling pathway	hsa04115	0.00012323	0.000167154	Hydroxycarbamide
POLE (7); *XRCC1* (2); *PARP1* (4)	Base excision repair	hsa03410	0.00016032	0.000192665	Iniparib
SEMA3A (3); *ROBO1* (6); *SEMA3G* (5);	Axon guidance	hsa04360	0.00024075	0.000374804	Valategrast hydrochloride
CHEK2 (5); *TP53* (6); *ATM* (6); *MCM6* (1); *E2F1* (1)	Cell cycle	hsa04110	0.00044405	0.00059063	Nilotinib hydrochloride hydrate, Nilotinib
ERCC4 (3); *POLE* (7); *ERCC1* (1)	Nucleotide excision repair	hsa03420	0.003679	0.004460381	
POLE (7); *MCM6* (1)	DNA replication	hsa03030	0.0094512	0.01106679	

Supplementary Table S11: List of primer sequences

**q-RT-PCR primers**	**Sequence 5' to 3'**
hCPM-Forward	GTCCTCTCTGCAAACCTCCAT
hCPM-Reverse	CCCAGTTGCTTGAACACCAT
hGAPDH-Forward	CTTCAACAGCGACACCCACT
hGAPDH-Reverse	GTGGTCCAGGGGTCTTACTC
hLPL-Forward	CCGAGAGTGAGAACATCCCATTCA
hLPL-Reverse	CCTTTCTGCAAATGAGACACTTTCTC
hNF1-Forward	TGTCAGTGCATAACCTCTTGC
hNF1-Reverse	AGTGCCATCACTCTTTTCTGAAG
**shRNA cloning primers**	**Sequence 5' to 3'**
hCPM shRNA1	CCGGCCAGTATGACTTGAATCGAAACTCGAGTTTCGATTCAAGTCATACTGGTTTTTG
hCPM shRNA2	CCGGCCAACAAATATGGAGAGTATTCTCGAGAATACTCTCCATATTTGTTGGTTTTTG
hCPM shRNA3	CCGGCCCTGAAATCACAAATCTGATCTCGAGATCAGATTTGTGATTTCAGGGTTTTTG
hCPM shRNA4	CCGGCCAGAACTTCAGTGCTCTTAACTCGAGTTAAGAGCACTGAAGTTCTGGTTTTTG
hNontarget shRNA	CCGGGCGCGATAGCGCTAATAATTTCTCGAGAAATTATTAGCGCTATCGCGCTTTTTG
hNF1 shRNA1	CCGGTGCGCAGTTAGCAGTTATAAACTCGAGTTTATAACTGCTAACTGCGCATTTTTG
hNF1 shRNA2	CCGGTTTAGATAGTCTCCGTATATTCTCGAGAATATACGGAGACTATCTAAATTTTTG

Supplementary Table S12: List of antibodies used in the study

Antibody	Catalog No.	Company
CPM	HPA002657	Sigma
CPM	ab49278	Abcam
Phospho-EGF Receptor (Tyr1068)	2234	Cell Signalling
EGF Receptor Antibody	2232	Cell Signalling
Phospho-ERK (E-4)	sc-7383	Santa Cruz
ERK 1/2 (MK1)	sc-135900	Santa Cruz
Phospho-Akt (Thr308) (L32A4)	5106	Cell Signalling
Akt (pan) (C67E7)	4691	Cell Signalling
p21 Waf1/Cip1 (12D1)	2947	Cell Signalling
Neurofibromin (D)	sc-67	Santa Cruz
GAPDH (14C10)	2118	Cell Signalling
β-Actin	A5316	Sigma
